# Network Meta-Analysis of Chicken Microarray Data following Avian Influenza Challenge—A Comparison of Highly and Lowly Pathogenic Strains

**DOI:** 10.3390/genes13030435

**Published:** 2022-02-26

**Authors:** Azadeh Moradi Pirbaluty, Hossein Mehrban, Saeid Kadkhodaei, Rudabeh Ravash, Ahmad Oryan, Mostafa Ghaderi-Zefrehei, Jacqueline Smith

**Affiliations:** 1 Department of Genetics and Animal Breeding, Faculty of Agriculture, Shahrekord University, Shahrekord 88186-34141, Iran; azadeh.moradi1993@gmail.com (A.M.P.); hosseinmehrban@gmail.com (H.M.); 2 Agricultural Biotechnology Research Institute of Iran (ABRII), Center of Iran, Isfahan 14968-13151, Iran; s_kadkhodaei@yahoo.com; 3 Department of Plant Breeding and Biotechnology, Faculty of Agriculture, Shahrekord University, Shahrekord 88186-34141, Iran; r.ravash@gmail.com; 4 Department of Pathology, School of Veterinary Medicine, Shiraz University, Shiraz 71557-13876, Iran; oryan1215@gmail.com; 5 Department of Genetics and Animal Breeding, Faculty of Agriculture, Yasouj University, Yasouj 75918-74831, Iran; 6 The Roslin Institute, University of Edinburgh, Easter Bush Campus, Midlothian EH25 9RG, UK

**Keywords:** microarray, network, meta-analysis, Python, influenza, chicken, transcriptome, HPAI, LPAI

## Abstract

The current bioinformatics study was undertaken to analyze the transcriptome of chicken (*Gallus gallus*) after influenza A virus challenge. A meta-analysis was carried out to explore the host expression response after challenge with lowly pathogenic avian influenza (LPAI) (H1N1, H2N3, H5N2, H5N3 and H9N2) and with highly pathogenic avian influenza (HPAI) H5N1 strains. To do so, ten microarray datasets obtained from the Gene Expression Omnibus (GEO) database were normalized and meta-analyzed for the LPAI and HPAI host response individually. Different undirected networks were constructed and their metrics determined e.g., degree centrality, closeness centrality, harmonic centrality, subgraph centrality and eigenvector centrality. The results showed that, based on criteria of centrality, the *CMTR1*, *EPSTI1*, *RNF213*, *HERC4L*, *IFIT5* and *LY96* genes were the most significant during HPAI challenge, with *PARD6G*, *HMG20A*, *PEX14*, *RNF151* and *TLK1L* having the lowest values. However, for LPAI challenge, *ZDHHC9*, *IMMP2L*, *COX7C*, *RBM18*, *DCTN3*, and *NDUFB1* genes had the largest values for aforementioned criteria, with *GTF3C5*, *DROSHA*, *ATRX*, *RFWD2*, *MED23* and *SEC23B* genes having the lowest values. The results of this study can be used as a basis for future development of treatments/preventions of the effects of avian influenza in chicken.

## 1. Introduction

Global monitoring of influenza is crucial for improvements in disease management, rapid intervention and decreasing the potential impact of an influenza pandemic. Avian Influenza (AI) is caused by three types of viruses: types A, B and C. Influenza A viruses (IAVs) are potentially zoonotic viruses that can cause infection in birds and a small number of mammals [1]. Influenza A virus is the only species of the alpha influenza virus genus in the Orthomyxoviridae family. Most human influenza pandemics of the 20th century were caused by IAVs that originated, either wholly or in part, from avian influenza A viruses [2]. The virus can be transmitted from wild birds to native poultry, which provides the opportunity for a zoonotic influenza epidemic [3], and can significantly affect the evolution of influenza viruses that circulate within human populations [4].

All strains of influenza A subtypes have been isolated from wild birds. Some isolates of influenza A virus cause severe disease in both domestic poultry and occasionally humans. The various subtypes are named according to the type of hemagglutinin and neuraminidase molecules present on the viral surface. There are 18 distinct known H antigens (H1 to H18) and 11 distinct known N antigens (N1 to N11) [5]. Based on their pathogenicity, avian influenza A subtypes can be classified into two classes: lowly pathogenic avian influenza (LPAI) and highly pathogenic avian influenza (HPAI). Lowly pathogenic avian influenza (LPAI) viruses rarely cause human infections but could contribute to future pandemic outbreaks; however, little is known about inter-species differences in the host responses to these viruses [6]. Studies are being undertaken to try and understand differences in the host response after challenge with viral strains with different propensities for evolution to high pathogenicity [7].

Human infections with the swine-origin influenza virus A (H1N1) were first detected in April 2009, and then spread rapidly across the globe. Children and young adults are particularly susceptible to the 2009 H1N1 virus infection because they have no or low immunity to the novel 2009 H1N1 strains [8,9]. The widespread and rapid distribution of the 2009 H1N1 viruses in humans raises a concern about the evolution of more virulent strains during passage in the population. One fear is that mutant forms of the 2009 H1N1 viruses may exhibit significantly increased virulence [10,11].

Highly pathogenic avian influenza (HPAI) H5N1 viruses cause severe infection in chickens at near complete mortality, but corresponding infection in ducks is typically mild or asymptomatic [12]. In particular, the Eurasian lineage of HPAI H5N1 virus causes severe disease in humans with a fatality rate of about 60% [13]. Most human influenza pandemics of the 20th century were caused by influenza A viruses (IAVs) that originated, either wholly or in part, from avian influenza A viruses [2]. Ducks and other waterfowl are reservoirs for most IAVs, including the hemagglutinin (HA) and neuraminidase (NA) subtypes that have caused previous human pandemics [14]. Despite being susceptible to infection with a wide range of IAVs, such birds often show little or no clinical signs [15,16]. In contrast, most HPAI H5N1 virus strains produce very severe disease in chickens, turkeys and quails, often causing up to 100% mortality within 2–3 days [17,18]. With their natural resistance, ducks support genetic reassortment of influenza viruses, providing a mechanism of evolution of genetically diverse IAVs including HPAI H5N1 viruses [19,20].

Naturally, viral genome mutations play a big role in the severity of viral diseases. For instance, PA-X is a newly discovered protein that is known to affect viral replication and host gene expression [21]. Loss of PA-X expression increases the viral virulence in mice, chickens, and mallard ducks, as shown in the reductions of virulence in the 1918 H1N1 pandemic virus in mice when PA-X expression was decreased [22]. Two CK10-based PA-X deficient viruses were created as subtypes of the H5N1 virus to demonstrate the ability of PA-X to reduce the severity of the H5N1 virus in mice, chickens, and ducks [23]. The influenza A virus endoribonuclease PA-X usurps RNA splicing to selectively target host RNAs for destruction. Proximity-labeling proteomics reveals that PA-X interacts with cellular RNA processing proteins, some of which are partially required for host shutoff. Thus, PA-X taps into host nuclear pre-mRNA processing mechanisms to destroy nascent mRNAs shortly after their synthesis. This mechanism sets PA-X apart from other viral host shutoff proteins that target actively translating mRNAs in the cytoplasm [24].

**Figure 1:**
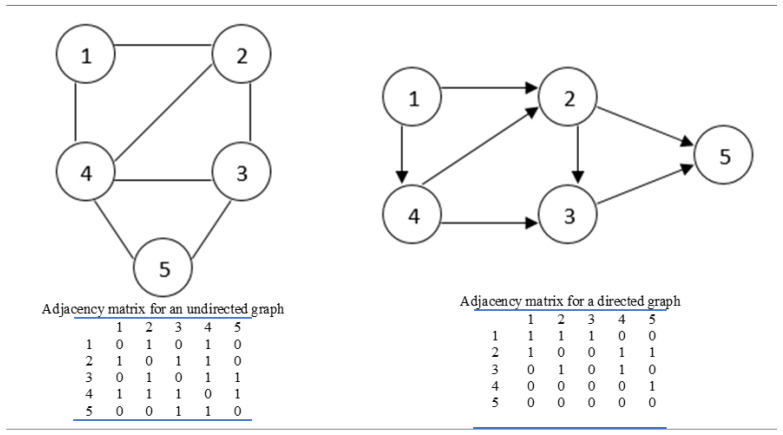
An indication of directed and undirected graphs showing their adjacency matrices.

Enhanced surveillance needs fast, robust and cheap analytical methods to provide a thorough analysis of influenza virus strains [25]. This is where sophisticated network-based tools can prove beneficial. The theory of intricate networks plays a main role in a wide variety of disciplines [26]. In general, the mathematical discipline which underpins the study of complex networks in biology is based on graph theory, where Graph G = (V, E) with V = vertices and E = edges. The edge may have direction (digraph) or no direction (multigraph). Figure 1 delineates both directed and undirected graphs with their adjacency matrices.

Potential applications within the field of biology include the identification of drug targets and the design of effective control strategies for infectious diseases [27], to name but two. A basic premise of designing a gene network is that knowledge regarding the structure of genetic relationships enriches existing knowledge of the function of each individual gene. Supplementary Table S1 summarizes some network-based studies that have been applied in poultry transcriptomic research. However, these network-based analyses have not been utilized for studying the host response to influenza infection in chicken. Gene network analysis goes beyond knowledge of single gene effects and shows relational interaction of numerous genes. This is therefore an effective way to construct gene modules to gain a deeper understating of the biological pathways and networks underpinning response to disease. In this study, for the first time, we use indirect graph analyses to look at the biological effects of influenza infection in chickens and identify the core genes involved in transcriptional change. We believe it is vital to understand the path and pace of the virus which culminates in the identified host–virus interactions, with a view to formulating decisions regarding mitigation strategies, virus containment, antiviral therapy and vaccination.

## 2. Materials and Methods

### 2.1. Datasets Used in This Study

**Figure 2:**
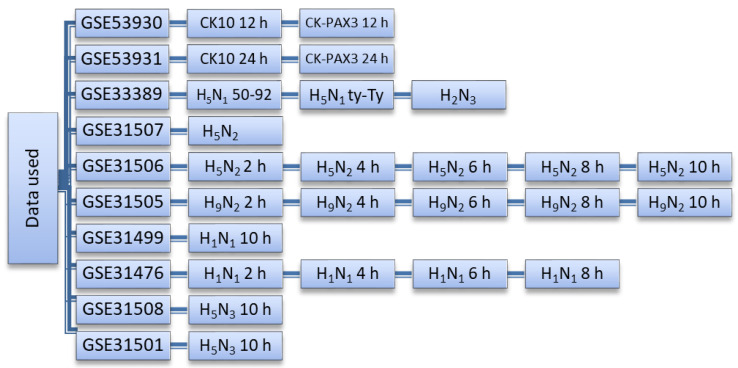
The datasets used and their corresponding viruses and sample collection times (post-infection). All samples are derived from lung tissue. H5N1-50-92 is a classical HPAI virus H5N1 strain (A/turkey/England/50-92/91) and H5N1-ty-Ty is a contemporary Eurasian lineage clade 2.2.1 H5N1 virus (A/turkey/Turkey/1/05).

Microarray expression profiles from chicken after influenza challenge were downloaded from the Gene Expression Omnibus (GEO) database (http://www.ncbi.nlm.nih.gov/geo (accessed on 22 October 2018). Data relating to LPAI studies included challenge with H2N3, H5N3, H9N2, H5N2 and H1N1 strains and data relating to HPAI involved H5N1 challenge. Each dataset consisted of infected and control groups. For HPAI, 24 samples were examined (consisting of 16 infected and 8 control samples) and for LPAI, 76 samples (consisting of 56 infected and 20 controls) were studied (Figure 2).

### 2.2. Normalization of Microarray Data

**Figure 3:**
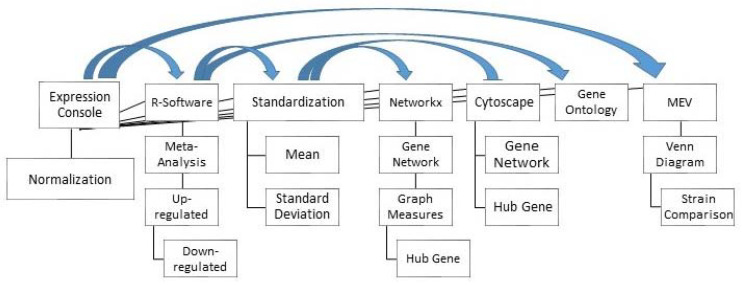
Analysis pipeline used in this study (Arrows indicate where results from one software were used as input for another program). MEV = multiple experiment viewer.

Figure 3 shows the bioinformatics pipeline used in this study. The CEL files from each dataset were normalized using the RMA normalization method in the Affymetrix Expression console software package (version: 1.1). RMA is a normalization algorithm for microarray data, correcting background, normalizing and summarizing the probe level information without the use of the information from the Mis-Match probe. Correct normalization is an absolute prerequisite for accurate evaluation of gene expression [28].

### 2.3. Network Construction

We used the rank product meta-analysis algorithm on normalized microarray data from the various avian influenza challenge experiments shown in Figure 2. The rank product is a non-parametric statistic that was originally aimed at identifying differentially expressed genes (DEGs) in a dataset [29]. Using the RankProd (RP) package (Version 1.1.383) [30], an add-on R package, a set of DEGs with FDR < 0.05 were obtained. The RP is a statistical approach that is widely used to detect differentially expressed features in -omics data e.g., transcriptomics, metabolomics and proteomics studies. Standardized expression values of meta-genes from all samples were used to rank and select the most important genes.

### 2.4. Derivation of Network Metrics

Up-regulated and down-regulated DEGs were used to train the network. Differentially expressed genes were obtained using meta-analysis by comparing fold change between disease and control groups from each data series. The gene expression data were then standardized. The Pearson correlation between the gene expression data was then obtained and genes with correlation values higher than 95% (ad-hoc) were used in Networkx (version 2.4 for python3.7 via anaconda) (https://networkx.github.io/ (accessed on 16 November 2018) to draw the undirected gene network and its metric measures cited in Table 1. NeworkX is a Python-based software package, used to extract the network metric measures, manipulate data, and study the structure, dynamics, and functions of complex networks (Supplementary Table S2). It has many standard graph algorithms for data structures for graphs, digraphs, and multigraphs. We developed individual networks based on both up-regulated and down-regulated genes. Investigation of complete sets of DEGs did not produce meaningful networks.

### 2.5. Downstream Analysis of Network Genes

The obtained results were used to construct a Venn diagram to identify genes overlapping between different strains. This was done using the Jvenn package (http://jvenn.toulouse.inra.fr/app/example.html (accessed on 20 May 2019). Gene IDs of DEGs were also uploaded to the PANTHER [31] website (http://www.pantherdb.org/ (accessed on 1 July 2019) to investigate the differences in gene expression between the samples after the viral challenge compared to the control samples. Network ranking algorithms created using Cytoscape software package ClueGo + Clue Pedia (version: 3.8.0.) (https://cytoscape.org/ (accessed on 28 September 2019) were then used to identify genes overlapping with the Python-based network results. Biological pathways, functions and networks relating to identified DEGs were investigated by means of Ingenuity Pathway Analysis (IPA) software (Qiagen: https://digitalinsights.qiagen.com/products-overview/discovery-insights-portfolio/analysis-and-visualization/qiagen-ipa/ (accessed on 12 April 2021). Differentially expressed genes with adjusted *p* < 0.05 and FC > 1.5 were used in the IPA analysis.

## 3. Results

### 3.1. Analysis of LPAI Datasets

**Figure 4:**
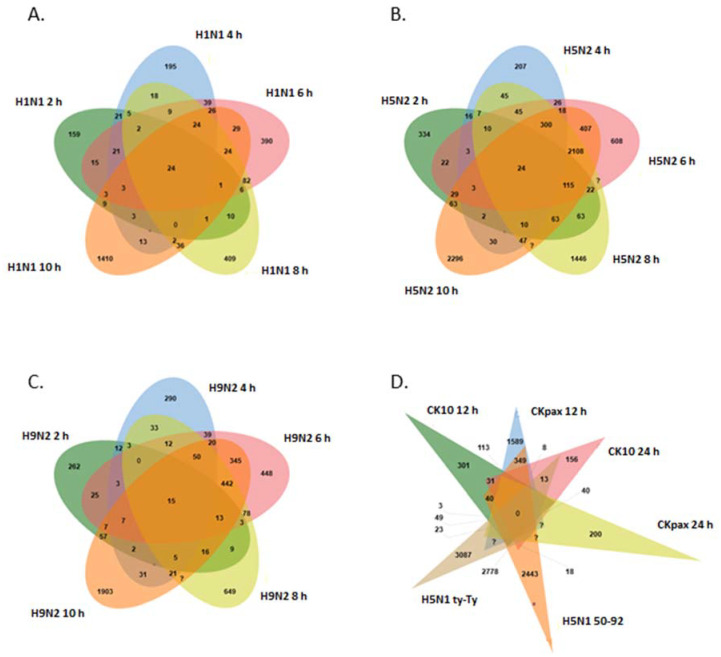
Venn diagram showing common genes across datasets and viral strains. (**A**) H1N1 across 2, 4, 6, 8 and 10 h, (**B**) H5N2 across 2, 4, 6, 8 and 10 h, (**C**) H9N2 across 2, 4, 6, 8 and 10 h, (**D**) H5N1.

In the LPAI datasets, 6004 probes (3535 genes) were differentially expressed (*p* < 0.05) with 31% of DEGs being up-regulated and 69% of DEGs down-regulated. For the H1N1 strain there were 9 common probes between 2 h and 10 h after viral infection (Figure 4A). For H5N2 there were 24 probes expressed at all times and there were 64 common probes expressed between 2 h and 10 h after viral infection (Figure 4B). For H9N2, there were 15 probes expressed across all times and there were 57 common probes between 2 h and 10 h after viral infection (Figure 4C). In terms of probes commonly expressed between different viral sub-types, we see 1520 common probes between H5N2 and H9N2, 271 probes between H5N2 and H1N1, 102 probes between H1N1 and H9N2, 294 probes between H5N2 and H5N3, 171 probes between H5N2, H9N2 and H1N1 and 210 probes between H5N2, H9N2 and H5N3. All LPAI DEGs (FC > +/−1.5) are shown in Supplementary Table S3.

### 3.2. Analysis of HPAI Datasets

The Venn diagram in Figure 4D shows that for H5N1 infections, there were 31 commonly expressed probes between Ck10 12 h and Ck10 24 h datasets; 23 between Ck-pax 12 h and Ck-pax 24 h and 2778 between H5N1 ty-Ty and H5N1 50–92. The largest set of common probes was found between Ck-pax 24 h, 50–92 and ty-Ty datasets, with 550 probes shared (Figure 4D). The result of the meta-analysis for chicken influenza microarray data showed that in the HPAI datasets examined, 390 probes (265 genes) were differentially expressed (*p* < 0.05). For DEGs, 29% were found to be up-regulated while 71% were down-regulated. A list of all significant differentially expressed genes is shown in Supplementary Table S4.

### 3.3. Comparison of LPAI and HPAI Datasets

**Figure 5:**
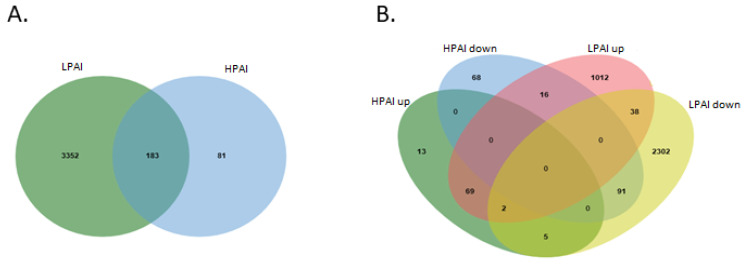
Intersected analysis was used for the identification of common DEGs from all ten datasets. (**A**) Total differentially expressed genes between HPAI and LPAI from across all possible group comparisons. (**B**) Venn diagram showing up- and down-regulated genes in HPAI and LPAI experiments. Different coloured regions represent different datasets, and the intersecting area denotes the common DEGs. (DEGs, differentially expressed genes; HPAI, highly pathogenic avian influenza; LPAI, lowly pathogenic avian influenza).

In order to shed some light on the differential gene expression between HPAI and LPAI challenge, Venn diagrams were used to identify overlapping DEGs between both the up-regulated and down-regulated gene sets (Figure 5). Collectively, this analysis, based on direct comparison of gene expression across the two influenza groups, demonstrated that there are 91 genes in common between down-regulated HPAI and LPAI datasets—the largest number of common genes. When up-regulated data are compared, there are seen to be 69 genes differentially expressed after both LPAI and HPAI challenge.

The purpose of this study was to identify the most important genes involved in the avian flu response, based on the location of these genes in a gene network. Meta-analysis was performed individually on LPAI and HPAI datasets for genes up-regulated and for those down-regulated. Table 1 shows the information relating to the Python networks generated for each challenge group. The nodes represent genes and edges represent interactions in the gene network. For LPAI data, the up-regulated network includes 1842 nodes and 60,606 edges, while the down-regulated network contains 4162 nodes and 243,604 edges. With the HPAI data, we see an up-regulated network including 305 nodes and 114 edges and a down-regulated network that includes 1813 nodes and 276 edges. Results from our network analyses are summarized in Table 2, which describes the hub genes identified in each of the up- or down-regulated groups.

As the centrality measure of the genes in the networks reduces, the proportion of the genes remaining in the network also reduces. Genes with high ‘centrality’, ‘harmonic’, ‘degree’, ‘closeness’, ‘Eigenvector’ and ‘Subgraph’ metrics achieve similar performance, whereas ‘betweenness centrality’ and ‘load centrality’ have a lower performance. Results from the ‘core number’ and ‘cluster’ criteria were not appropriate for finding modules. The ‘degree centrality’ metric in gene networks defines the importance of a gene in a graph as being measured based on its degree; the higher the degree of a gene, the more important it is in a graph. According to the results of the present study, the ‘degree centrality’ criterion obtained more accurate results than other criteria, as the results from this criterion are consistent with the results of the gene network based on the correlation between the genes. Centrality criteria for different DEGs are as shown in Table 2.

**Figure 6:**
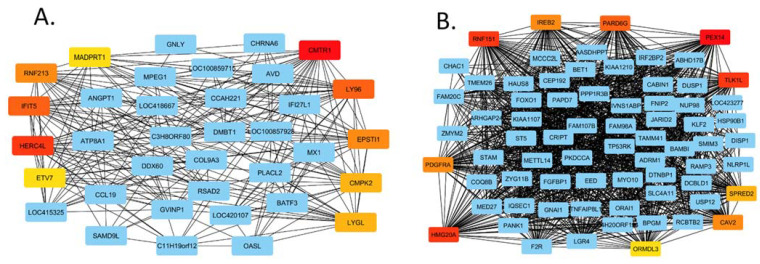
Cytoscape gene networks of the up-regulated HPAI dataset (**A**) and down-regulated HPAI dataset (**B**).

The networks created are too large to present pictorially, but as an example, we show a section of the HPAI networks as depicted using Cytoscape software. Figure 6A shows a section of the up-regulated network and Figure 6B part of the down-regulated gene network.

Examining genes differentially expressed between the HPAI and LPAI data sets identified the genes summarized in Table 3. *CMTR1*, *HERC4L*, *IFIT5*, *LY96*, *RNF213* and *EPSTI1* were all more highly expressed after HPAI challenge compared to LPAI challenge. It is interesting to note that several of these genes (*IFIT5*, *LY96*, *RNF213* and *EPST1*) were previously identified in a study examining LPAI and HPAI infection in ducks and chickens [32]. However, *RBM18*, *NDUFB1*, *DCTN3*, *COX7C*, *IMMP2* and *LZDHHC9* all showed higher expression during the host response to LPAI.

### 3.4. Analysis of All Up- and All Down-Regulated Genes

We also performed a general analysis across all up- (Table 4) and all down-regulated (Table 5) genes from both HPAI and LPAI datasets and identified significant genes. In general, we see a fairly unique response to LPAI and HPAI infections. However, from our network analysis, some genes were identified as important hub genes across both types of challenge. Amongst genes up-regulated, *SELENOK*, *NDUFA1*, *PPP1R7*, *SMDT1*, *COX7C*, *PRELID3B*, *CIB1*, *OST*4 and *NDUFB2* were highlighted. Some of these (*SELENOK*, *SMDT1*, *CIB1*) are involved in calcium signaling, while others (*NDUFA1*, *COX7C*, *NDUFB2*) play a role in the mitochondrial respiratory chain. Ca^2+^- dependent signaling plays a crucial role in influenza viral internalization and infection, as well as being implicated in apoptosis of viral-infected cells [33]. An increase in activity of the mitochondrial respiratory chain is also known to occur after influenza infection [34]. Significant down-regulated genes included *PUS10*, *ERBIN*, *SYDE2*, *PCGF6*, *FZD6*, *ROR1*, *LRIG2*, *SUPT7L*, *EXOC8*, *KIF1C* and *PCM*1. These genes have a variety of functions including miRNA processing (PUS10), RNA polymerase II-specific transcription repressor activity (*PCGF6*), and negative regulation of biological processes including cell proliferation and apoptosis (*FZD6*) and chromatin modification (*SUPT7L*). Many of these genes are thus seen to have a role in how other genes are regulated.

### 3.5. Gene Ontology

Analysis of functional ontologies associated with the genes being differentially expressed in response to influenza infection was carried out. Supplementary Tables S5–S8 show the biological processes, molecular functions and cellular components associated with DEGs identified as being up-regulated in response to HPAI (Supplementary Table S5), down-regulated in response to HPAI (Supplementary Table S6), up-regulated in response to LPAI (Supplementary Table S7) and down-regulated in response to LPAI (Supplementary Table S8). Based on KEGG analysis, important pathways affected during response to HPAI include those of Influenza A, Cytokine-cytokine receptor interaction, NOD-like receptor signaling and Toll-like receptor signaling, whereas after LPAI challenge cell adhesion molecules, MAPK signaling, ErbB signaling and phagosome activity are modified.

### 3.6. Pathway Analysis

**Figure 7:**
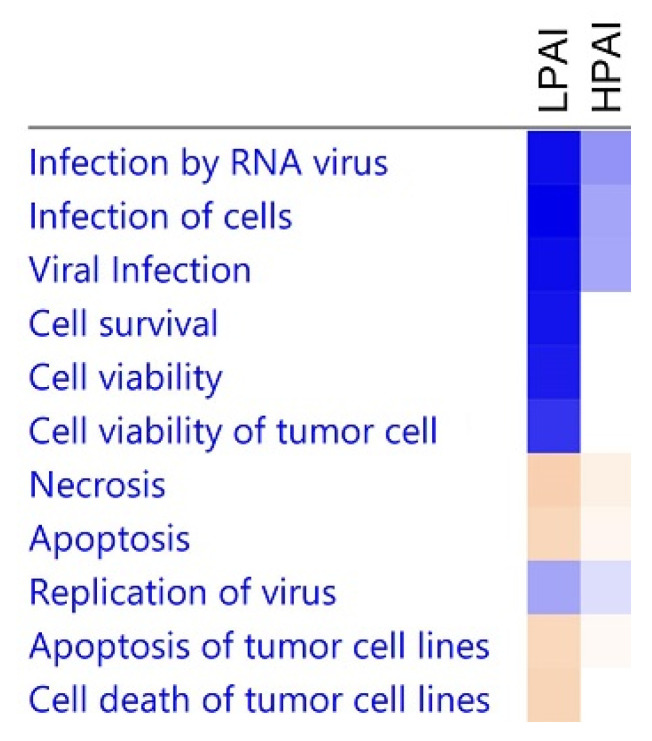
A comparison of biological activities differentially regulated upon LPAI or HPAI challenge. Comparison as determined by IPA software. DEGs with *p* < 0.05 and FC > 1.5 were used in the analysis. Shades of blue indicate down-regulation and shades of orange up-regulation.

In order to explore the biological pathways and functional processes associated with differentially expressed genes, Ingenuity Pathway Analysis software was used. Figure 7 shows a comparison of some of the most significant biological activities related to the DEGs with respect to LPAI challenge compared to HPAI disease. Viral infection and replication is lower after LPAI challenge and apoptosis higher. This higher level of cell death is mirrored by the lower levels of cell survival indicated. This reflects the host ability to kill AI-infected cells and ultimately overcome LPAI infection—a situation not enabled when chickens succumb to HPAI virus. Upon investigation of the gene networks being modified upon HPAI challenge, we see that *IFNG* and *IFNB* are two of the main activators of gene expression. Conversely, genes such as *IL4*, *IL10* and *STAT6* are inhibited, restricting a Th2-type immune response (Supplementary Table S9), directing the host toward an antiviral Th1 response. Supplementary Figure S1 shows the interferon-stimulated inflammatory response that is initiated after the H5N1 HPAI infection investigated in this study.

## 4. Discussion

Here we present a network analysis of a variety of microarray datasets representing avian influenza challenge in chicken. These datasets represent both lowly pathogenic and highly pathogenic infections. Our network modelling identifies core genes central to the response to both kinds of challenge, whether that be through up- or down-regulation of gene expression.

We observed that degree, harmonic and closeness centrality methods generate highly significant results. These methods are reliable applicants for use in practice to identify the hub genes related to a particular disease [35]. Load and betweenness centrality methods do not have a higher statistical significance than the baseline method [34] in highlighting known avian influenza genes. However, degree, harmonic and closeness centrality parameters can identify the previously unknown genes which are involved in the disease response of interest. These methods can be used to generate new hypotheses on host-virus interaction, and highlight candidates for experimental validation. Gene networks were used to identify the functional relevance of a gene interacting with communicating nodes in a biological network. The higher the value, the higher the relevance of the gene in connecting the regulatory molecules [36]. One can easily read basic features of the graph (degree, hierarchical structure, etc.) as well as more nuanced features, e.g., the relationship between a vertex and the hierarchical position of its neighbors. The present visualization strategy is a useful tool in discriminating between networks with different topological properties and structural arrangement, and may be also used for comparison of models with real data, providing an additional tool for model validation [37].

For each type of challenge (LPAI and HPAI), unique and common host response genes were identified. During LPAI infection, genes involved in the process of apoptosis are activated, compared with the HPAI response. This may be one of the reasons why birds are able to overcome lowly pathogenic strain infections but not that of the highly pathogenic variety. On the other hand, immune stimulation and an interferon response is seen after HPAI challenge. However, as chickens are unable to survive the HPAI challenge, this response is either (1) inadequate in overcoming the viral infection or (2) too extreme and causes the so-called ‘cytokine storm’ often responsible for mortality in the face of HPAI infection. The levels of cytokine expression that are seen here do not appear to be excessive however, so it is likely that the response that is being initiated, in this instance, is insufficient. Indeed, only 96 genes are seen to be significantly up-regulated in response to HPAI (with 4.9-fold maximum expression change). A strong down-regulation of several genes is however seen. These include immune genes such as *BLB1*, *CXCR4*, *IRF2BP2*, *TLR5* and *TNFRSF1B*. So it may be that HPAI infection causes an overall down-regulation of the immune system, thus rendering the host incapable of mounting a sufficient response to the virus.

Most of the up-regulated LPAI DEGs are involved in functions associated with the mitochondria or Golgi apparatus. Flu infection is known to lead to alterations in mitochondria morphology, release of pro-apoptotic proteins, loss of mitochondrial membrane potential, and eventually cell death [38]. When we look at the up-regulated HPAI DEGs on the other hand, we see that several are involved in the innate immune response, with *CMTR1* and *IFIT5* being well-known interferon-stimulated genes, *LY96* enhancing TLR4-dependent activation of NF-kappa-B [39] and *EPSTI1* mediating RELA/p65 and STAT1 phosphorylation and nuclear localization upon activation of macrophages [40].

Looking at core genes identified across all viral challenges, genes up-regulated in both highly and lowly pathogenic infections include those involved in calcium signaling –a process central to biological activity after viral infection. Various viruses enter host cells via endocytosis, but the underlying molecular mechanisms are unknown. The influenza A viruses (IAVs) enter cells via redundant pathways of clathrin-mediated and clathrin-independent endocytosis, with intracellular calcium having a central role in regulation of both pathways, by activating a signaling axis comprising RhoA, Rho-kinase, phosphatidylinositol 4-phosphate 5-kinase (PIP5K) and phospholipase C (PLC). IAV infection induces oscillations in the cytosolic Ca^2+^ concentration of host cells, the prevention of which markedly attenuates virus internalization and infection. The small GTPase RhoA is found both to function downstream of the virus-induced Ca^2+^ response and to induce Ca^2+^ oscillations in a manner dependent on Rho-kinase and subsequent PIP5K-PLC signaling. This signaling circuit regulates both clathrin-mediated and clathrin-independent endocytosis during virus infection and seems to constitute a key mechanism for regulation of IAV internalization and infection [33].

Other genes with a role in the mitochondrial respiratory chain were also identified. Genes commonly down-regulated encompass a variety of functions, but many were involved in gene regulation. The Cap Methyltransferase 1 (*CMTR1*) gene was identified as a core up-regulated gene in the HPAI gene expression network. This gene has been identified as a host dependency factor, vital for efficient viral cap-snatching and regulating cell autonomous immune response. It also provides synergistic protection with the influenza endonuclease inhibitor Xofluza [41]. *CMTR1* also has a potential role in the pathogenesis of asthma exacerbations [42]. Also in the HPAI gene expression network, the *PARD6G* gene (Par-6 Family Cell Polarity Regulator Gamma) was seen to be down-regulated. It is thought to play a role in the formation of epithelial tight junctions [43]. The expression changes occurring in these genes could help explain the differences in mortality seen between LPAI and HPAI infection in chickens.

## 5. Conclusions

We have used network analysis methods to predict hub gene associations from a collection of microarray datasets known to be related to the avian response to influenza infection, and created gene-interaction networks by correlation amongst differentially regulated genes. Next, we used degree, eigenvector, closeness, betweenness, subgraph, harmonic centrality, clustering and core number metrics to rank the genes in the network according to their relevance in the system. Our method has enabled the identification of core genes involved in the general host response to influenza infection in chicken e.g., *SELENOK*, *NDUFA1*, *PPP1R7*, *SMDT1*, *COX7C*, *PRELID3B*, *CIB1*, *OST4* and *NDUFB2* amongst up-regulated data and *PUS10*, *ERBIN*, *SYDE2*, *PCGF6*, *FZD6*, *ROR1*, *LRIG2*, *SUPT7L*, *EXOC8*, *KIF1C* and *PCM1* from down-regulated data. Comparison between different pathogenic strains identifies up-regulation of *CMTR1*, *HERC4L*, *IFIT5*, *LY96*, *RNF213* and *EPSTI1* as being significant during HPAI challenge, with *RBM18*, *NDUFB1*, *DCTN3*, *COX7C*, *IMMP2* and *LZDHHC9* being central to the LPAI expression network.

## Figures and Tables

**Table 1:** Python Networks.

Python Network for LPAI Microarray Data		
**Regulation**	Number of Nodes	Number of Edges
up-regulation	1842	60,606
down-regulation	4162	243,604
**Python Network for HPAI Microarray Data**		
**Regulation**	Number of Nodes	Number of Edges
up-regulation	305	114
down-regulation	1813	276

LPAI—lowly pathogenic avian influenza; HPAI—highly pathogenic avian influenza; nodes = genes; edges = interactions.

**Table 2:** Top 6 hub genes identified from each network analysis.

LPAI: Up-Regulated Genes						
**Gene**	**Number of Connections**	**Degree**	**Harmonic**	**Closeness**	**Eigenvector**	**Subgraph**
*RBM18*	384	0.37	647.81	0.5	0.07	1.21 × 10^103^
*NDUFB1*	375	0.36	639.53	0.49	0.07	1.17 × 10^103^
*DCTN3*	372	0.36	641.19	0.5	0.07	1.16 × 10^103^
*COX7C*	370	0.36	639.55	0.49	0.06	1.03 × 10^103^
*IMMP2L*	368	0.36	638.38	0.49	0.07	1.16 × 10^103^
*ZDHHC9*	368	0.36	639.43	0.5	0.07	1.11 × 10^103^
**LPAI: Down-Regulated Genes**						
**Gene**	**Number of Connections**	**Degree**	**Harmonic**	**Closeness**	**Eigenvector**	**Subgraph**
*GTF3C5*	849	0.36	1491.63	0.52	0.06	8.47 × 10^184^
*RFWD2*	840	0.36	1493.43	0.52	0.05	8.04 × 10^184^
*MED23*	828	0.35	1486.05	0.52	0.05	7.78 × 10^184^
*SEC23B*	798	0.34	1469.68	0.52	0.05	7.52 × 10^184^
*ATRX*	796	0.34	1450.85	0.5	0.05	6.77 × 10^184^
*DROSHA*	794	0.34	1456.03	0.5	0.05	7.31 × 10^184^
**HPAI: Up-Regulated Genes**						
**Gene**	**Number of Connections**	**Degree**	**Harmonic**	**Closeness**	**Eigenvector**	**Subgraph**
*CMTR1*	27	0.52	37.08	0.61	0.26	2.23 × 10^6^
*HERC4L*	24	0.47	35.58	0.58	0.24	1.91 × 10^6^
*IFIT5*	23	0.45	34.11	0.53	0.24	1.84 × 106
*LY96*	23	0.45	34.03	0.52	0.21	1.45 × 10^6^
*RNF213*	22	0.43	33.61	0.52	0.24	1.78 × 10^6^
*EPSTI1*	22	0.43	33.53	0.52	0.22	1.56 × 10^6^
**HPAI: Down-Regulated Genes**						
**Gene**	**Number of Connections**	**Degree**	**Harmonic**	**Closeness**	**Eigenvector**	**Subgraph**
*PEX14*	60	0.45	81.7	0.5	0.16	5.62 × 10^17^
*HMG20A*	59	0.44	81.2	0.5	0.16	5.53 × 10^17^
*TLK1L*	59	0.44	81.2	0.5	0.16	5.52 × 10^17^
*RNF151*	59	0.44	81.78	0.51	0.16	5.43 × 10^17^
*PARD6G*	58	0.43	80.78	0.5	0.15	5.01 × 10^17^
*CAV2*	55	0.41	79.2	0.49	0.15	4.88 × 10^17^

**Table 3:** Comparison of differential gene expression found between HPAI and LPAI datasets.

Gene Symbol	Gene Name	Entrez Gene ID	HPAI		PAI	
			Log2 Fold Change	Regulation	Log2 Fold Change	Expression
*CMTR1*	cap methyltransferase 1	14306	2.10	UP		
*HERC4L*	hect domain and RLD 4-like	4297	2.05	UP		
*IFIT5*	interferon induced protein with tetratricopeptide repeats 5	33635	2.64	UP		
*LY96*	lymphocyte antigen 96	5508	1.91	UP		
*RNF213*	ring finger protein 213	10972	2.06	UP		
*EPSTI1*	epithelial stromal interaction 1	11241	2.01	UP		
*RBM18*	RNA binding motif protein 18	7150			1.30	UP
*NDUFB1*	NADH:ubiquinone oxidoreductase subunit B1	4970			1.27	UP
*DCTN3*	dynactin subunit 3	4824			1.29	UP
*COX7C*	cytochrome c oxidase subunit 7C	4726			1.32	UP
*IMMP2L*	inner mitochondrial membrane peptidase subunit 2	9006			1.27	UP
*ZDHHC9*	zinc finger DHHC-type containing 9	2532			1.19	UP
*PEX14*	peroxisomal biogenesis factor 14	6768	−0.34	down		
*HMG20A*	high mobility group 20A	20936	−0.34	down		
*TLK1L*	tousled like kinase 1 like	6751	−0.27	down		
*RNF151*	ring finger protein 151	9782	−0.36	down		
*PARD6G*	par-6 family cell polarity regulator gamma	9912	−0.42	down		
*CAV2*	caveolin 2	9078	−0.36	down		
*GTF3C5*	general transcription factor IIIC subunit 5	21008			−0.70	down
*RFWD2*	ring finger and WD repeat domain 2	37706			−0.64	down
*MED23*	mediator complex subunit 23	8401			−0.68	down
*SEC23B*	Sec23 homolog B, coat complex II component	21262			−0.76	down
*ATRX*	alpha thalassemia/mental retardation syndrome X-linked	7476			−0.68	down
*DROSHA*	drosha ribonuclease III	20908			−0.70	down

**Table 4:** Hub genes for all up-regulated data.

Gene Symbol	Gene Name	Probe ID	Number of Connections
*SELENOK*	selenoprotein K	Gga.1058.1.S1_at	209
*NDUFA1*	NADH:ubiquinone oxidoreductase subunit A1	Gga.5918.1.A1_a_at	207
*PPP1R7*	protein phosphatase 1 regulatory subunit 7	Gga.5583.1.S1_a_at	202
*SMDT1*	single-pass membrane protein with aspartate rich tail 1	Gga.9946.1.S1_at	200
*COX7C*	cytochrome c oxidase subunit 7C	Gga.6171.1.S1_a_at	200
*PRELID3B*	PRELI domain containing 3B	Gga.9900.2.S1_at	199
*CIB1*	calcium and integrin binding 1	Gga.5965.2.S1_a_at	198
*OST4*	oligosaccharyltransferase complex subunit 4, non-catalytic	Gga.6184.1.S1_at	196
*NDUFB2*	NADH:ubiquinone oxidoreductase subunit B2	Gga.17299.1.S1_a_at	196

**Table 5:** Hub genes for all down-regulated data.

Gene Symbol	Gene Name	Probe ID	Number of Connections
*PUS10*	pseudouridylate synthase 10	GgaAffx.4897.1.S1_at	509
*ERBIN*	erbb2 interacting protein	Gga.17560.1.S1_at	496
*SYDE2*	synapse defective Rho GTPase homolog 2	Gga.11842.1.S1_s_at	492
*PCGF6*	polycomb group ring finger 6	Gga.16959.1.S1_at	490
*FZD6*	frizzled class receptor 6	Gga.2690.1.S1_at	489
*ROR1*	receptor tyrosine kinase like orphan receptor 1	Gga.9476.1.S1_at	485
*LRIG2*	leucine rich repeats and immunoglobulin like domains 2	Gga.17165.1.S1_at	479
*SUPT7L*	SPT7 like, STAGA complex gamma subunit	Gga.16763.1.S1_at	475
*EXOC8*	exocyst complex component 8	Gga.14199.1.S1_at	470
*KIF1C*	kinesin family member 1C	Gga.15878.1.S1_s_at	469
*PCM1*	pericentriolar material 1	Gga.3449.1.S1_at	468

## Data Availability

All data examined in this study are as outlined in Figure 2 along with relevant accession numbers.

## Supplementary Materials

The following supporting information can be downloaded at: https://www.mdpi.com/article/10.3390/genes13030435/s1, Table S1: Previous network based studies in chicken [44,45,46,47,48,49,50,51,52,53,54,55,56,57,58,59,60], Table S2: Network metrics used in this study [61,62,63,64,65,66,67], Table S3: Differentially expressed genes from LPAI data analysis, Table S4: Differentially expressed genes from HPAI data analysis, Table S5: Gene Ontology of up-regulated HPAI DEGs, Table S6: Gene Ontology of down-regulated HPAI DEGs, Table S7: Gene Ontology of up-regulated LPAI DEGs, Table S8: Gene Ontology of down-regulated LPAI DEGs, Table S9: HPAI upstream activators.
