# Supplementary material for: Network Meta-Analysis of Chicken Microarray Data following Avian Influenza Challenge—A Comparison of Highly and Lowly Pathogenic Strains
Source: Genes (Basel). 2022 Feb 26;13(3):435. doi: 10.3390/genes13030435 (PMC8953847; doi:10.3390/genes13030435)
Supplement: Supplementary file 1 [file genes-13-00435-s001.zip › genes-1586274-supplementary.pdf]

Supplementary Table S1: A non-exhaustive review of transcriptomic based network construction in chicken

Technology	Software	Tissue	Publication
Microarray	MIDAS, SAS	Hypothalamus	Higgins et al., 2010 [44]
Microarray	GeneSpring, Beacon Designer	Kidney	Cong et al., 2013 [45]
NGS	DNASTAR	Spleen, intestine	Hong et al., 2014 [46]
RNA-Seq	Deseq	Mucus	Luo et al., 2014 [47]
RNA-eq,qPCR	Fastx_clipper Fastx_toolkit, GSNAP	Liver	Coble et al., 2014 [48]
Microarray	GeneSpring, SPSS	Intestine	Kim et al., 2014 [49]
RNA-Seq	Toolx, Fastx, FastQC, TopHat2, edgeR, R	Thymus	Sun et al., 2016 [50]
RNA-Seq	DNASTAR, SAS	Breast muscle	Chen et al., 2015 [51]
Unigenes	bowtie2, Mega, BLAST comparison	Lymphocytes	Tariq et al. 2015 [52]
Microarray	Affymetrix GCOS, Expression Console	Embryonic	Naraballobh et al., 2016 [53]
RNA –Seq	FastQC, clipper fastX, Packet DESeq2	Liver	Lan et al., 2016 [54]
RNA-Seq	HTSeq, DES-R, KOBAS	Kidney	Liu et al., 2017 [55]
Microarray	GENESPRING, CYTOSCAPE	Liver, spleen	Ma et al., 2017 [56]
RNA –Seq	CytoScape, STRING, SPSS	Yellow Follicles	Wang et al., 2017 [57]
RNA –Seq	SPSS, FastQC, RSeQC, edgeR	Thyroid	Xie et al., 2018 [58]
RNA –Seq	SPSS, DESeq	Leg muscle	Wu et al., 2018 [59]
RNA –Seq	FastQC, NGN QC Toolkit, DESeq	Spleen	Qiu et al., 2018 [60]

Supplementary Table S2: The network metric measures used in this study

Explanation	Formula	Measures
The betweenness centrality of a node is the numeral of shortest paths between further nodes that run through the node of interest [61].	$B_v = \sum_{i=1}^{G-1} \sum_{j=i+1}^{G} \frac{c_{i.j(v)}}{c_{i.j}}$	Betweenness Centrality
The closeness centrality of a node measures the centrality of a node based on how near it is to other nodes in the network [62].	$C_i = \frac{1}{\sum_{nj \in N \setminus \{ni\}} H_{ni \to nj}}$	Closeness Centrality
Degree centrality is the number of nodes that a given node is connected to [62]. The degree ki of node i is computed as follows.	$K_i = \sum_{j=1}^{n} A_{ij}$	Degree Centrality
Eigenvector centrality assigns each node a centrality that not only depends on the quantity of its connections, but also on their qualities [63].	$x_j = \frac{1}{\lambda} \sum_{j=1}^{n} A_{ij}\, x_j$	Eigenvector
The harmonic mean is normalized by noting that on a star graph, the maximum is obtained by the node in the center and is |V|−1 [64].	$C_H = \frac{2(n-1) \sum_i \left(C_{H\,max_j} - C_H(x_j) \right)}{n}$	Harmonic Mean
Load centrality a property fully defined by the graph structure and by the algorithm used to discover minimum weight paths [65].	$LC_v = \sum_{s.d \in v} \theta_{s.d\,(v)}$	Load Centrality
Counts the times that a node takes part in the different connected subgraphs of the network, with smaller subgraphs having higher importance [66].	$S(i) = \sum_{k=0}^{\infty} \frac{w_k(i)}{k!}$	Subgraph
Clustering based on the concept of subgraph intensity, defined as the geometric average of subgraph edge weights, resulting edge weights are normalized by the maximum weight in the network [67].	$c_u = \frac{2T(u)}{deg(u)(deg(u)-1)}$	Clustering
The average clustering criterion is obtained by calculating the average clustering criterion.	$c = \frac{1}{n} \sum_{u \in G} c_u$	Average Clustering

Supplementary Table S3: Differentially expressed genes from LPAI data analysis

Gene	Log2 Fold change	Fold change	p-value
GPX8	2.45	5.48	0.001797
COL9A1	2.44	5.44	0.0003596
OSGIN1	2.39	5.23	0.0006649
TNFSF10	2.37	5.16	0.0004972
TF	2.34	5.06	0.002374
RFFL	2.32	5.00	0.002177
MXD4	2.13	4.37	0.0004258
TCP11L2	2.10	4.28	0.0007636
COMMD8	2.06	4.17	0.002014
PCMTD1	2.05	4.15	5.15E-07
RIPK3	2.05	4.15	2.96E-06
EPYC	2.05	4.13	0.0005793
KRT15	2.05	4.13	9.27E-05
GEM	2.04	4.12	7.16E-07
IL13RA1	2.04	4.12	3.16E-05
APAF1	2.03	4.08	0.001919
FABP4	2.03	4.08	0.001022
IKBIP	2.02	4.07	0.002005
ARHGAP8	2.02	4.06	0.001385
BORCS6	2.02	4.05	0.001384
TIPARP	2.00	4.01	0.001271
GPR1	2.00	4.00	0.0008197
CMBL	2.00	4.00	3.33E-05
LY75	2.00	4.00	0.0002498
CD44	1.99	3.98	7.26E-05
CHODL	1.99	3.98	0.0004185
CPNE8	1.99	3.98	3.15E-05
CUL2	1.98	3.95	0.00032
CYTL1	1.98	3.94	0.001869
NKTR	1.97	3.93	0.001218
COL9A3	1.97	3.91	4.39E-06
PLSCR1	1.97	3.91	0.001127
AKR1D1	1.97	3.90	0.001444
EDNRB	1.96	3.90	0.001222
CDH1	1.95	3.87	0.001578
MYD88	1.95	3.87	6.54E-06
ZCCHC2	1.95	3.86	6.82E-05
TNFRSF6B	1.95	3.85	0.0007762
SAT1	1.94	3.85	0.0008462
NANP	1.94	3.83	2.07E-07
PPP1R17	1.94	3.83	0.0008319
CD200R1L	1.93	3.82	0.001524
BIRC2	1.93	3.82	0.001222
GLIPR1L	1.93	3.81	0.002011
HTR2B	1.93	3.81	0.0005804
RAB31	1.93	3.81	0.001539
SLC46A2	1.93	3.81	0.0006222
PIT54	1.93	3.80	8.48E-06

FBP1	1.92	3.79	0.001728
CG-16	1.92	3.78	0.002077
FUNDC1	1.92	3.78	0.00156
GFPT2	1.92	3.77	0.001181
VNN1	1.92	3.77	0.0003668
CUBN	1.91	3.77	0.001498
EDNRA	1.91	3.76	0.002143
CHST15	1.90	3.73	0.00166
COMP	1.90	3.73	0.001082
C1QTNF6	1.90	3.72	0.0008608
ARHGAP15	1.89	3.72	5.61E-05
LPCAT3	1.89	3.71	0.0004842
NAT9	1.89	3.70	0.0007216
ASB9	1.89	3.70	0.0009752
SPP1	1.89	3.70	0.001311
CAPN9	1.89	3.69	0.0006847
LYRM1	1.88	3.69	0.0002464
SERPIND1	1.88	3.68	0.001339
TMEM168	1.88	3.68	3.49E-05
CHST2	1.88	3.68	0.001622
RAB36	1.88	3.68	0.0008856
YPEL5	1.88	3.68	0.001143
FMR1	1.88	3.68	0.0001082
SPP2	1.88	3.67	9.89E-05
N4BP2L1	1.88	3.67	0.0008834
CSTF3	1.88	3.67	0.000139
TTR	1.87	3.67	0.001284
HAS2	1.87	3.66	5.23E-05
ABRACL	1.87	3.66	0.0009105
ACSL1	1.87	3.66	0.0004569
UBE2V2	1.87	3.65	0.001754
STX17	1.87	3.65	0.0001348
TANGO2	1.87	3.65	0.001454
APOA1	1.86	3.64	1.96E-05
FGB	1.86	3.64	0.0001041
BFAR	1.86	3.64	0.00109
MAPK11	1.86	3.63	0.001449
PDE4B	1.86	3.63	0.0006793
HSPB11	1.86	3.63	0.002272
PKIG	1.86	3.63	0.000472
ARRDC1	1.86	3.62	0.0001534
FCF1	1.86	3.62	0.0006475
NSMCE2	1.86	3.62	0.001505
GLUL	1.86	3.62	3.20E-07
BCL2L10	1.85	3.62	0.0009866
IL16	1.85	3.61	0.0003105
CDKN2B	1.85	3.61	0.0007953
FAM195A	1.85	3.61	0.001876
TIMM21	1.85	3.61	0.0001278
CETN3	1.85	3.61	0.000797

PRPS2	1.85	3.61	0.0001945
UBE2H	1.85	3.60	0.001322
GSTO1	1.85	3.60	0.001696
PTS	1.85	3.60	0.001484
FAM180A	1.85	3.60	0.001696
UPP1	1.85	3.59	0.001305
LYRM2	1.84	3.59	0.0004162
WIPI1	1.84	3.59	0.00196
NINJ1	1.84	3.58	2.98E-06
HOXA6	1.84	3.58	0.001086
NUDT7	1.84	3.58	0.001611
SDHAF2	1.84	3.58	0.001189
LOC420807	1.84	3.57	4.29E-05
PLAGL1	1.83	3.56	0.0002544
DYNLT1	1.83	3.56	0.001124
IDNK	1.83	3.56	0.0003305
LOC769756	1.83	3.56	0.00213
NDFIP1	1.83	3.56	0.0001619
NPL	1.83	3.55	0.0009393
SNRNP25	1.83	3.55	0.0009809
NUDT5	1.83	3.55	0.0003548
CYBRD1	1.83	3.55	0.00129
WDSUB1	1.83	3.54	0.0006421
TRERF1	1.83	3.54	0.00155
IRF6	1.83	3.54	2.40E-05
KLF9	1.82	3.54	0.0004913
CBWD1	1.82	3.54	0.001192
PTGS2	1.82	3.54	0.001339
PTP4A1	1.82	3.54	0.001226
NDP	1.82	3.53	0.0006586
MALL	1.82	3.53	2.74E-05
PHYKPL	1.82	3.53	0.001164
SLC40A1	1.82	3.53	0.0003653
IFNAR2	1.82	3.53	0.001838
SERPINB2	1.82	3.53	9.89E-05
MMP15	1.82	3.52	0.0005942
DACT2	1.82	3.52	0.0007958
SULT1C3	1.82	3.52	0.001222
FNDC3A	1.82	3.52	1.86E-05
HPGD	1.81	3.52	0.0001599
ITGB3BP	1.81	3.52	0.001639
LOC416951	1.81	3.51	0.001276
PDCD6	1.81	3.51	0.0006717
CCLL4	1.81	3.51	0.0001266
SELENOP1	1.81	3.51	0.0007952
PSCA	1.81	3.51	0.0002428
UBL3	1.81	3.50	0.001221
MOSPD1	1.81	3.50	0.0002518
LOC424401	1.81	3.50	0.0005672
HNRNPKL	1.81	3.50	0.0007943

DDTNFR23	1.81	3.50	0.002283
FABP1	1.81	3.50	0.001786
ABLIM2	1.81	3.50	0.001684
LOC772080	1.81	3.50	0.001199
TPMT	1.81	3.50	3.38E-05
EIF4E2	1.81	3.50	0.001
EPB41L3	1.80	3.49	0.001824
MMP9	1.80	3.49	0.001849
PCYT2	1.80	3.49	0.001237
C22H8ORF4	1.80	3.49	0.001222
KCNK12	1.80	3.49	0.0001029
GABRA5	1.80	3.49	0.002223
ADCYAP1	1.80	3.48	0.002332
MND1	1.80	3.48	0.002349
RAMAC	1.80	3.48	0.0007331
CENPW	1.80	3.48	0.001577
GSTK1	1.80	3.48	0.001263
CMKLR1	1.80	3.48	0.001384
ATP6V1G3	1.80	3.48	0.00182
NCF4	1.80	3.48	0.0002489
LGI1	1.80	3.48	0.0003024
COMMD2	1.80	3.48	0.0004191
SOX7	1.80	3.48	0.0002751
PUDP	1.80	3.48	0.0005211
ASTL	1.80	3.48	0.0008907
SLCO4C1	1.80	3.47	0.0004637
GLRX5	1.80	3.47	0.0002885
AZIN2	1.80	3.47	0.0001853
FAM171B	1.80	3.47	0.0003974
GPR137B	1.80	3.47	2.83E-06
HOXD8	1.79	3.47	0.0006426
MAP3K7CL	1.79	3.47	0.000812
IL20RA	1.79	3.47	0.0008163
ST6GAL1	1.79	3.47	0.0003176
GPX1	1.79	3.47	0.0008233
COMMD7	1.79	3.46	0.002099
NIM1KZ	1.79	3.46	3.34E-06
PACRG	1.79	3.46	0.0009806
PBDC1	1.79	3.46	0.001972
FETUB	1.79	3.46	0.0006797
PTCHD1	1.79	3.46	0.0002648
MYOZ2	1.79	3.46	0.001601
CALML4	1.79	3.46	0.0005663
GPR143	1.79	3.46	0.0003212
RNF11	1.79	3.46	0.0006759
IL10RB	1.79	3.46	0.0002011
HOXD12	1.79	3.46	0.0002027
FGF7	1.79	3.46	0.001488
PLCXD1	1.79	3.46	0.0008777
ALDH1A1	1.79	3.46	0.0002369

LOC101748561	1.79	3.45	0.0001944
LOC101751113	1.79	3.45	0.0002619
CCK	1.79	3.45	0.001789
SIGMAR1	1.79	3.45	0.001984
RPA3	1.78	3.45	0.0008517
ETFRF1	1.78	3.44	0.001262
KRT6A	1.78	3.44	0.0006926
LIPA	1.78	3.44	0.001049
ZDHHC9	1.78	3.44	0.00135
BNIP3	1.78	3.44	1.49E-05
NT5C1B	1.78	3.44	0.001078
HOXB6	1.78	3.44	0.001461
LHX4-AS1	1.78	3.44	0.001168
MRPL33	1.78	3.44	0.001304
MT3	1.78	3.44	0.00155
KRT5	1.78	3.44	1.41E-05
CTBS	1.78	3.44	0.0009421
KXD1	1.78	3.44	0.0001883
INS-IGF2	1.78	3.44	0.000635
SELENOW	1.78	3.44	0.001351
UBE2F	1.78	3.44	0.001791
TRPV2	1.78	3.43	0.0003174
OAZ2	1.78	3.43	0.000538
SQSTM1	1.78	3.43	0.000367
LOC768772	1.78	3.43	0.0003251
GJB6	1.78	3.43	0.001221
RER1	1.78	3.43	0.002343
BEAN1	1.78	3.43	0.000167
VSNL1	1.78	3.43	0.002127
SLC30A5	1.78	3.43	0.002254
AK3	1.78	3.43	0.0004638
CASTOR2	1.78	3.42	0.0006379
CYP2AC1	1.78	3.42	0.001488
FAIM	1.78	3.42	0.0007969
NFKBIA	1.78	3.42	1.79E-07
TMEM9B	1.78	3.42	0.0008676
DPY30	1.78	3.42	0.001453
CAPZA2	1.78	3.42	0.002368
MME	1.77	3.42	0.0005868
TNFRSF21	1.77	3.42	0.00205
RGS7BP	1.77	3.42	0.0001794
AADAT	1.77	3.42	0.0004942
BTN1A1	1.77	3.42	0.0005262
TNIP2	1.77	3.42	0.0001942
GCG	1.77	3.42	0.001015
RHOC	1.77	3.42	1.30E-06
RBM43	1.77	3.42	1.70E-06
LAMTOR3	1.77	3.41	0.00173
LOC101749574	1.77	3.41	0.0003864
ESRP2	1.77	3.41	1.83E-05

MRAS	1.77	3.41	0.0007813
HAGH	1.77	3.41	0.001623
MAP1LC3B2	1.77	3.41	0.0001273
APOH	1.77	3.41	0.0001917
CAB39L	1.77	3.41	9.09E-05
C5H11orf58	1.77	3.41	0.001721
LOC424109	1.77	3.41	0.00131
MYL3	1.77	3.41	0.0001319
FAHD2A	1.77	3.41	0.0009794
MRPS33	1.77	3.41	6.33E-05
SPIK5	1.77	3.40	3.21E-07
FBLIM1	1.77	3.40	0.0002013
GYG1	1.77	3.40	0.0001032
KLHL5	1.77	3.40	0.001216
ELL	1.77	3.40	0.002208
VAT1	1.77	3.40	0.0002006
TOR4A	1.77	3.40	1.48E-05
IGSF21	1.77	3.40	0.00154
AVDL	1.77	3.40	0.0003983
DPM1	1.76	3.40	0.0003314
B4GALNT4	1.76	3.40	0.000329
NAIF1	1.76	3.40	0.001613
HDAC8	1.76	3.39	0.00083
HMGA1	1.76	3.39	0.001848
LOC101749502	1.76	3.39	0.000833
CCL17	1.76	3.39	0.001571
FNDC5	1.76	3.39	0.0006811
BAG2	1.76	3.39	0.002345
ADNP	1.76	3.39	0.001692
GPX4	1.76	3.39	0.001164
THEMIS2	1.76	3.39	0.001313
TRA2A	1.76	3.39	0.001689
SF3A2	1.76	3.39	0.0004571
TMEM138	1.76	3.39	0.0001201
EDMPN1	1.76	3.39	0.0001864
SCARA5	1.76	3.39	0.001551
UPF2	1.76	3.39	0.001098
WFDC2L	1.76	3.39	0.001443
NDUFB3	1.76	3.39	0.0008818
MPPED1	1.76	3.39	0.0002662
MTUS2	1.76	3.39	0.00148
FBXW8	1.76	3.38	6.21E-05
PIP5K1B	1.76	3.38	0.0009525
SLC3A1	1.76	3.38	0.00142
PAK1IP1	1.76	3.38	0.001915
CH25H	1.76	3.38	3.68E-06
TMEM248	1.76	3.38	0.0006493
LYRM4	1.76	3.38	7.67E-05
COA3	1.76	3.38	0.0007025
LOC769121	1.76	3.38	0.001441

NMU	1.76	3.38	0.0008331
ASS1	1.76	3.38	1.11E-07
IL15	1.76	3.38	0.0005526
PPP1R2	1.75	3.37	0.0011
APC2	1.75	3.37	0.001599
HOXB5	1.75	3.37	8.22E-06
NGF	1.75	3.37	0.0003106
CTNNBL1	1.75	3.37	0.0004636
LOC101748084	1.75	3.37	0.002373
IRF2	1.75	3.37	0.0004167
CACNG3	1.75	3.37	0.0001455
SPATA22	1.75	3.37	0.001642
BG8	1.75	3.37	0.002255
ANGPTL1	1.75	3.37	5.31E-05
CENPS	1.75	3.37	0.000675
RPS14	1.75	3.37	0.002345
GUK1	1.75	3.37	5.91E-05
MKRN2OS	1.75	3.37	0.00232
AQP9	1.75	3.37	0.0001922
ZNF821	1.75	3.36	0.000552
LSP1	1.75	3.36	0.0009663
DALRD3	1.75	3.36	0.002225
SLC30A4	1.75	3.36	0.001316
GRTP1	1.75	3.36	0.000418
MTAP	1.75	3.36	0.0001685
ZDHHC6	1.75	3.36	0.0002195
EIF2AK1	1.75	3.36	0.001974
CDA	1.75	3.36	0.001016
TMEM200C	1.75	3.36	0.0002257
LOC101749012	1.75	3.36	0.0003407
MAP3K8	1.75	3.36	0.001266
KIAA1143	1.75	3.36	7.49E-05
GNG13	1.75	3.36	0.001315
TNFAIP2	1.75	3.36	0.001958
SF3B5	1.75	3.36	0.0006068
MGST3	1.75	3.36	0.001112
AvBD9	1.75	3.36	6.26E-06
TIMP3	1.75	3.36	0.0003652
RPL35A	1.75	3.36	0.0001249
HIKESHI	1.75	3.36	0.0008933
PIGA	1.75	3.36	8.21E-06
ANGPTL7	1.75	3.35	0.001597
RFKL	1.75	3.35	0.000357
PPIP5K1	1.75	3.35	0.001961
HOMER2	1.75	3.35	0.001299
SLC25A14	1.74	3.35	0.0006437
LOC417056	1.74	3.35	9.12E-05
ARHGDIB	1.74	3.35	0.0008455
MFAP5	1.74	3.35	0.002351
PGS1	1.74	3.35	0.0006136

DDT	1.74	3.35	0.001767
WDFY2	1.74	3.35	5.73E-06
MGAT4B	1.74	3.35	0.0008417
MRGPRH	1.74	3.35	0.0005413
RFXANK	1.74	3.35	0.0001623
AVP	1.74	3.35	0.001616
DPCD	1.74	3.35	0.0008412
SNX3	1.74	3.35	0.0005434
SPCS1	1.74	3.35	0.001709
DLEC1	1.74	3.35	0.0002332
LIF	1.74	3.35	0.001576
VSTM2L	1.74	3.35	0.001851
ENY2	1.74	3.35	0.001325
COLEC10	1.74	3.34	0.001878
RBBP4	1.74	3.34	3.91E-05
VSIG10L	1.74	3.34	0.0006331
PNISR	1.74	3.34	0.001275
ANGPT1	1.74	3.34	0.0001981
SEMA3F	1.74	3.34	0.001278
CISD3	1.74	3.34	0.000106
C11H19orf12	1.74	3.34	0.0002498
RIMS3	1.74	3.34	0.001825
WRAP73	1.74	3.34	0.001225
IL18	1.74	3.34	0.0001113
GLYR1	1.74	3.34	0.001607
MIEN1	1.74	3.34	0.001398
RIPK4	1.74	3.34	7.21E-06
TMEM121L	1.74	3.34	0.001789
TMPRSS2	1.74	3.34	0.0007267
TRIM8	1.74	3.34	0.002058
NDUFB4	1.74	3.34	0.001983
SS18L2	1.74	3.34	0.001302
CTSB	1.74	3.34	0.0005234
NHP2	1.74	3.34	0.0001601
NME4	1.74	3.33	0.001297
LEPROTL1	1.74	3.33	0.0004784
KDELC2	1.74	3.33	0.0007374
CALB2	1.74	3.33	0.0008188
SMIM10L1	1.74	3.33	0.001553
CCNI	1.74	3.33	6.28E-05
CHRNB2	1.74	3.33	0.00168
TPM2	1.74	3.33	0.001328
EIF4H	1.74	3.33	0.0006512
CARMIL1	1.74	3.33	0.0005049
CKS1B	1.74	3.33	0.000547
CMTR1	1.74	3.33	0.001555
FGF2	1.74	3.33	0.0008131
LSM5	1.74	3.33	0.0005114
PRRG3	1.74	3.33	0.001305
C14H16orf45	1.73	3.33	0.001402

PFDN1	1.73	3.33	0.0006793
DYNLRB2	1.73	3.33	0.0004255
TMEM242	1.73	3.33	2.70E-05
LOC100858311	1.73	3.33	0.001682
PHYHD1	1.73	3.33	0.0008214
PI3	1.73	3.33	0.0005995
USP16	1.73	3.33	0.0009885
GPX2	1.73	3.33	0.0005084
TIMP4	1.73	3.33	8.13E-05
ATP5ME	1.73	3.33	0.002188
GPX7	1.73	3.33	0.001216
FAM194A	1.73	3.33	0.001644
TMBIM4	1.73	3.33	5.95E-05
ADAMTS15	1.73	3.32	0.002072
KIAA1671	1.73	3.32	0.002385
TXNDC17	1.73	3.32	0.0002673
GATA3	1.73	3.32	0.0002344
HPSE2	1.73	3.32	0.001062
SLC36A4	1.73	3.32	0.001696
WDR88	1.73	3.32	0.002319
PRELID1	1.73	3.32	0.000129
CXCL12	1.73	3.32	0.0005034
MSX2	1.73	3.32	0.001007
CRTAC1	1.73	3.32	0.0006133
RSPO3	1.73	3.32	0.0004611
B4GALT2	1.73	3.32	0.0004483
CZH9ORF3	1.73	3.32	0.001092
GPAT4	1.73	3.32	0.0002065
CSTB	1.73	3.32	0.0001054
AN1ZNF5L	1.73	3.32	0.0001056
ZMAT5	1.73	3.32	0.001048
TRUB2	1.73	3.32	0.0001181
C5AR1	1.73	3.32	0.001942
MRPL52	1.73	3.32	0.001043
TDO2	1.73	3.31	0.0002915
SLC4A8	1.73	3.31	0.002345
LOC771972	1.73	3.31	0.001047
FXYD2	1.73	3.31	0.0003815
LOC101750765	1.73	3.31	0.000393
NTAN1	1.73	3.31	0.0002622
URM1	1.73	3.31	0.0001245
TBX5	1.73	3.31	0.0002121
URAH	1.73	3.31	0.001464
LSMEM1	1.73	3.31	0.0002532
SELENOU	1.73	3.31	0.001152
WSB2	1.73	3.31	0.0004641
ARL6IP5	1.73	3.31	0.000443
MRPS25	1.73	3.31	0.0007921
SMOX	1.73	3.31	3.59E-05
PIN4	1.73	3.31	0.0002044

CD83	1.73	3.31	0.0001895
CENPV	1.73	3.31	1.71E-05
IMMP2L	1.73	3.31	9.02E-05
OGFR	1.73	3.31	6.84E-05
HSBP1L1	1.73	3.31	0.002269
COL16A1	1.73	3.31	0.001036
SMIM7	1.72	3.31	0.0006083
XPO1	1.72	3.30	0.0002878
CHCHD7	1.72	3.30	3.30E-05
CABP2	1.72	3.30	0.0004968
CASP1	1.72	3.30	1.33E-05
GBP1	1.72	3.30	0.0002371
MORN4	1.72	3.30	0.0001608
LOC100859471	1.72	3.30	0.002102
S100A4	1.72	3.30	0.0002494
TOMM6	1.72	3.30	0.001881
XPR1	1.72	3.30	0.0002813
APC	1.72	3.30	0.0008635
NDUFB1	1.72	3.30	0.0006332
PHACTR2	1.72	3.30	0.0001178
TPPP	1.72	3.30	0.0004253
JMJD4	1.72	3.30	7.58E-05
SEC24A	1.72	3.30	0.0001595
ANGPT1L	1.72	3.30	0.0007716
UBE2D3	1.72	3.30	9.71E-05
DNTTIP1	1.72	3.30	0.0001197
TLR3	1.72	3.30	0.0004614
TRIM66	1.72	3.30	0.0001633
LOC418701	1.72	3.30	0.001531
RAB34	1.72	3.30	0.001188
FABP6	1.72	3.30	6.38E-05
EEF1E1	1.72	3.30	0.00126
LOC101749087	1.72	3.29	7.95E-05
PARP11	1.72	3.29	0.002158
TM2D1	1.72	3.29	9.62E-05
ZCCHC13	1.72	3.29	9.91E-05
COL3A1	1.72	3.29	0.0001422
EIF4E3	1.72	3.29	2.04E-05
EOGT	1.72	3.29	0.0002824
HMGN5	1.72	3.29	0.001501
LGALSL	1.72	3.29	0.0006053
JADE2	1.72	3.29	0.001274
PTGES3L	1.72	3.29	1.85E-05
MRPL2	1.72	3.29	0.0004097
RNF150	1.72	3.29	0.0001921
LOC101750652	1.72	3.29	0.001045
PDCD1LG2	1.72	3.29	0.000209
SLC32A1	1.72	3.29	0.001018
ZDHHC21	1.72	3.29	3.36E-05
TTLL9	1.72	3.29	0.0008188

PITX2	1.72	3.28	0.0003045
HOXA10-AS	1.72	3.28	7.71E-05
ETV7	1.71	3.28	0.0002982
SNRPG	1.71	3.28	0.0006842
LOC396479	1.71	3.28	0.0001554
NT5C3A	1.71	3.28	0.0001708
CTC1	1.71	3.28	0.0008524
HNRNPA2B1	1.71	3.28	0.001651
MPC1	1.71	3.28	0.0001608
FHL1	1.71	3.28	0.0004021
MZT1	1.71	3.28	7.22E-05
NPM3	1.71	3.28	0.000158
PCASP3	1.71	3.28	3.35E-05
ATP8A1	1.71	3.28	5.54E-06
EI24	1.71	3.28	0.0002143
CSRP3	1.71	3.28	2.60E-05
DYNLT3	1.71	3.28	0.0008696
CRB1	1.71	3.28	0.0003798
GUCY1B4	1.71	3.28	0.000321
CMC2	1.71	3.27	0.0001616
PPP1R7	1.71	3.27	0.0005367
PTRHD1	1.71	3.27	0.001423
DCTN3	1.71	3.27	0.0003208
GKAP1	1.71	3.27	7.09E-05
CALHM6	1.71	3.27	0.0003547
SSTR4	1.71	3.27	0.002183
KCNMB1	1.71	3.27	9.97E-07
SLC25A20	1.71	3.27	0.0002823
LOC426097	1.71	3.27	0.0008101
LOC420486	1.71	3.27	5.81E-07
PPP2CB	1.71	3.27	0.001087
MBP	1.71	3.27	0.0007939
PLEKHA7	1.71	3.27	4.52E-05
WDR31	1.71	3.27	0.0008647
GGT5	1.71	3.27	0.0003733
SLC12A3	1.71	3.27	0.0003882
ARHGEF28	1.71	3.27	0.0002785
SLC25A16	1.71	3.27	9.75E-05
PTN	1.71	3.27	0.0005696
MRPS16	1.71	3.27	0.0002494
ROMO1	1.71	3.27	0.000126
PAM16	1.71	3.26	7.09E-05
ASPG	1.71	3.26	5.18E-05
SHROOM4	1.71	3.26	0.001555
SMIM4	1.71	3.26	0.0001307
FAM122B	1.71	3.26	0.0001109
MRPS21	1.71	3.26	7.62E-05
ADORA2B	1.71	3.26	0.0002652
FABP3	1.71	3.26	4.46E-06
ZIC2	1.71	3.26	0.001654

RPS27L	1.71	3.26	0.00088
SLC35E3	1.71	3.26	0.002038
SNU13	1.71	3.26	1.31E-05
HIVEP1	1.71	3.26	0.002215
IGFBP4	1.71	3.26	0.0006945
LYZ	1.71	3.26	0.001946
RASSF3	1.70	3.26	0.002201
FBXW4	1.70	3.26	6.99E-05
LOC101751481	1.70	3.26	0.001926
NEK10	1.70	3.26	0.002019
TGM2	1.70	3.26	6.10E-06
TAF12	1.70	3.26	0.0001073
EPDR1	1.70	3.26	0.0005242
PPM1J	1.70	3.26	6.93E-05
NFKBIZ	1.70	3.26	1.40E-06
FGF10	1.70	3.26	0.0001127
SNX22	1.70	3.26	0.001755
CHURC1	1.70	3.26	0.0007654
CCDC141	1.70	3.25	0.001593
GINM1	1.70	3.25	7.63E-07
PLEKHB2	1.70	3.25	9.90E-05
SNRPC	1.70	3.25	0.0005657
UCKL1	1.70	3.25	0.000815
NRSN1	1.70	3.25	9.95E-05
CRYGN	1.70	3.25	0.00174
MROH2B1	1.70	3.25	0.0002784
FDX1L	1.70	3.25	1.85E-05
OLFML1	1.70	3.25	0.000111
MED31	1.70	3.25	2.11E-05
STRADB	1.70	3.25	0.001355
DCTN5	1.70	3.25	1.74E-05
NAA20	1.70	3.25	0.0003919
HMGN3	1.70	3.25	0.0003207
C12orf40	1.70	3.25	0.0009513
COPS9	1.70	3.25	0.001742
CYCS	1.70	3.25	0.0001081
GNG4	1.70	3.25	9.19E-05
UPF3A	1.70	3.25	0.0002979
KCNK10	1.70	3.25	0.0009066
RBM18	1.70	3.25	4.72E-05
ZNF692	1.70	3.25	0.0003479
TMEM251	1.70	3.25	0.001011
IGDCC3	1.70	3.25	0.0002236
C1H11ORF70	1.70	3.25	2.89E-05
RPRD2	1.70	3.25	0.0007822
CLSTN3	1.70	3.25	0.001148
PFDN5	1.70	3.25	0.0002375
RSL24D1	1.70	3.25	7.04E-05
LOC101747452	1.70	3.25	0.001004
CXCL14	1.70	3.25	2.50E-05

NCOA7	1.70	3.25	0.0001221
JAM2	1.70	3.25	1.43E-05
LY6CLEL	1.70	3.25	8.90E-08
PVALB	1.70	3.25	5.64E-05
IL1RL1	1.70	3.24	2.47E-05
GBE	1.70	3.24	0.0005691
LOC395325	1.70	3.24	5.10E-05
RPL29	1.70	3.24	0.0001149
PATL2	1.70	3.24	6.83E-05
KAT2B	1.70	3.24	5.21E-06
EIF4EBP1	1.70	3.24	0.0001623
NARF	1.70	3.24	0.0003135
ATP5MF	1.70	3.24	0.00153
TMEM243	1.70	3.24	2.39E-10
CARD8	1.70	3.24	9.86E-09
TIFA	1.70	3.24	0.000219
RFC1	1.70	3.24	0.002265
HSF1	1.70	3.24	4.14E-06
NAMPT	1.70	3.24	3.06E-10
DNAJC18	1.69	3.24	0.0003815
SMDT1	1.69	3.24	0.0001394
SPTBN5	1.69	3.24	3.01E-05
LGALS3	1.69	3.24	1.16E-05
LOC420411	1.69	3.23	0.002205
PSMG4	1.69	3.23	3.41E-05
ARIH1	1.69	3.23	0.0006212
DLAT	1.69	3.23	0.0005872
LOC101751448	1.69	3.23	0.0002817
MBNL3	1.69	3.23	0.0001733
DRAM2	1.69	3.23	4.37E-05
TG	1.69	3.23	0.0004852
PECAM1	1.69	3.23	0.0003725
RILPL2	1.69	3.23	3.13E-05
LOC422757	1.69	3.23	0.001465
NDUFB2	1.69	3.23	0.0002204
SFSWAP	1.69	3.23	0.0005176
LAMTOR2	1.69	3.23	5.09E-05
PTGDS	1.69	3.23	2.61E-05
RGS10	1.69	3.23	0.0003639
CALML3	1.69	3.23	4.71E-06
TMEM100	1.69	3.23	4.62E-05
LOC420716	1.69	3.23	7.46E-05
TNFAIP6	1.69	3.23	0.0001839
ERG	1.69	3.23	0.0005866
NBN	1.69	3.23	1.71E-07
SMCHD1	1.69	3.22	3.67E-07
SLC25A32	1.69	3.22	0.001532
TRIM41	1.69	3.22	0.001553
SMIM14	1.69	3.22	0.0001796
RTKN2	1.69	3.22	0.001463

COX7C	1.69	3.22	0.00027
MKRN1	1.69	3.22	1.25E-05
COA5	1.69	3.22	0.0005508
RPL38	1.69	3.22	0.001341
BLB1	1.69	3.22	0.0009221
COTL1	1.69	3.22	0.000212
MAB21L1	1.69	3.22	0.0009555
C10H15orf40	1.69	3.22	8.78E-06
BRIP1	1.69	3.22	0.0004465
NLRC5	1.69	3.22	0.0002643
CLK1	1.69	3.22	0.0002499
USP30	1.69	3.22	6.97E-05
RPL22L1	1.69	3.22	3.46E-05
ATP5F1E	1.69	3.22	5.96E-06
KCNJ5	1.69	3.22	1.63E-09
SMAD7	1.69	3.22	0.0002353
HMX3	1.69	3.22	0.001683
PARP6	1.68	3.22	4.15E-05
ACOT7	1.68	3.21	7.43E-05
STMP1	1.68	3.21	0.0005718
CATH1	1.68	3.21	0.001327
PDZK1IP1	1.68	3.21	0.0002875
SRP9	1.68	3.21	1.91E-05
HMGA2	1.68	3.21	0.001146
LOC771277	1.68	3.21	0.001091
ETS1	1.68	3.21	0.001519
GNG2	1.68	3.21	1.91E-05
C17H9orf16	1.68	3.21	3.67E-05
LOC771161	1.68	3.21	8.34E-05
TWIST2	1.68	3.21	9.18E-07
HOXA7	1.68	3.21	3.10E-06
TXNDC12	1.68	3.21	0.0002857
GBX2	1.68	3.21	7.24E-05
HRAS	1.68	3.21	2.43E-05
ALDOB	1.68	3.21	2.56E-08
GLIPR2	1.68	3.21	7.23E-05
SOX9	1.68	3.21	0.001155
TMEM173	1.68	3.21	0.0001358
THYN1	1.68	3.21	2.58E-05
EVL	1.68	3.20	8.00E-04
CHMP6	1.68	3.20	1.39E-06
ENS-1	1.68	3.20	6.46E-07
SH3BGRL3	1.68	3.20	5.69E-06
RNF19B	1.68	3.20	7.95E-10
SSU72	1.68	3.20	3.22E-05
RPS12	1.68	3.20	0.0002133
TRIAP1	1.68	3.20	7.53E-05
TMEM123	1.68	3.20	0.0004099
HOXC10	1.68	3.20	1.92E-06
LOC408038	1.68	3.20	1.63E-05

RPLP2	1.68	3.20	0.0001804
MCHR2	1.68	3.20	0.0002831
LOC419842	1.68	3.19	4.17E-05
B3GAT3	1.67	3.19	4.69E-06
LOC396480	1.67	3.19	0.0002147
IRF5	1.67	3.19	0.001228
RRH	1.67	3.19	0.0001643
OLFM1	1.67	3.19	0.0003214
LMO7	1.67	3.19	6.52E-05
PCBP3	1.67	3.19	0.0001247
TTN	1.67	3.19	0.0001749
NTN4L	1.67	3.19	5.69E-05
WWC1	1.67	3.19	3.98E-06
FOXJ1	1.67	3.19	4.68E-06
DUS1L	1.67	3.19	0.0003736
PRELID3B	1.67	3.18	2.97E-05
NPDC1	1.67	3.18	0.0003662
NBL1	1.67	3.18	2.55E-05
TPT1	1.67	3.18	3.45E-05
COLQ	1.67	3.18	0.0001216
CX3CL1	1.67	3.18	0.0001759
TIMM13	1.67	3.18	4.32E-06
DUSP23	1.67	3.18	1.30E-05
PPIC	1.67	3.18	9.13E-05
BSG	1.67	3.18	3.73E-05
ADAMTS6	1.67	3.18	0.000443
HOXC9	1.67	3.18	3.62E-08
ACOD1	1.67	3.18	0.0003372
C1R	1.67	3.18	1.13E-06
DCTN6	1.67	3.18	2.73E-05
GKN2	1.67	3.18	3.66E-05
LOC426640	1.67	3.18	1.18E-05
FAM168A	1.67	3.18	7.21E-05
METTL6	1.67	3.18	2.88E-05
TGFB1	1.67	3.18	3.68E-05
MSRB3	1.67	3.18	0.0002332
LOC101748460	1.67	3.17	7.60E-05
FKBP7	1.67	3.17	8.80E-05
SH2D4B	1.67	3.17	9.55E-05
CABP7	1.67	3.17	0.0003841
FAM133B	1.67	3.17	4.44E-05
CREBRF	1.67	3.17	1.24E-05
IL5RA	1.67	3.17	0.0003508
PRMT8	1.67	3.17	4.98E-06
BMF	1.66	3.17	3.96E-07
LOC100857215	1.66	3.17	4.76E-05
PPY	1.66	3.17	0.0001549
ATP6AP1	1.66	3.17	0.0001755
LAPTM4A	1.66	3.17	0.000296
SERPINA10	1.66	3.17	4.58E-06

CHTOP	1.66	3.17	2.42E-05
ACOT2	1.66	3.17	5.32E-05
FUT10	1.66	3.17	1.24E-06
GNLY	1.66	3.17	0.0004364
GMDS	1.66	3.17	0.0001524
OSTF1	1.66	3.17	3.88E-05
LMO2	1.66	3.17	2.69E-06
DNAJB14	1.66	3.16	9.35E-05
EBAG9	1.66	3.16	1.14E-06
GHRL	1.66	3.16	2.26E-06
HYPK	1.66	3.16	1.56E-05
IL4I1	1.66	3.16	4.08E-06
FKBP1A	1.66	3.16	4.97E-05
TRANK1	1.66	3.16	4.27E-05
C9ORF58	1.66	3.16	0.0001783
CA3B	1.66	3.16	1.29E-06
CTTN	1.66	3.15	0.002365
SLC13A4	1.66	3.15	0.0003011
ZDHHC4	1.66	3.15	1.27E-07
LOC101749803	1.66	3.15	0.001796
RAP2C	1.66	3.15	4.02E-05
APIP	1.66	3.15	2.26E-05
XG	1.66	3.15	1.35E-05
ANKRD42	1.66	3.15	0.0001488
FANCA	1.66	3.15	0.0001727
CSTA	1.66	3.15	2.99E-06
C1orf53	1.66	3.15	3.66E-06
SELENOK	1.66	3.15	1.70E-05
CD99L2	1.65	3.15	7.13E-05
FAM198A	1.65	3.15	0.002122
RBX1	1.65	3.15	1.81E-05
TFPI	1.65	3.15	1.97E-06
TSPO	1.65	3.14	2.62E-06
NAA38	1.65	3.14	8.56E-05
PTGES	1.65	3.14	2.52E-05
C1H12ORF57	1.65	3.14	1.23E-06
RPS25	1.65	3.14	1.87E-05
ASIC1	1.65	3.14	0.0003976
TMEM14A	1.65	3.14	1.07E-06
UBE2D1	1.65	3.14	1.23E-06
JPH3	1.65	3.14	5.46E-07
PNKD	1.65	3.14	6.97E-05
SRC	1.65	3.14	0.001362
THY1	1.65	3.13	8.31E-05
GTF3C6	1.65	3.13	2.20E-06
ABCC6	1.65	3.13	0.001373
CFD	1.65	3.13	5.00E-05
YF6	1.65	3.13	6.65E-06
HSCB	1.65	3.13	1.38E-05
USMG5	1.65	3.13	5.66E-06

LOC420770	1.65	3.13	2.77E-07
TMEM61	1.65	3.13	9.25E-07
ASPN	1.65	3.13	4.06E-06
LGALS2	1.65	3.13	4.27E-06
HOXB4	1.65	3.13	3.43E-06
MPV17	1.65	3.13	2.84E-06
LOC769729	1.65	3.13	3.49E-07
USP4	1.64	3.13	0.001951
PYGB	1.64	3.13	0.001689
VPS72	1.64	3.13	1.06E-05
HCFC2	1.64	3.13	0.00116
SGCE	1.64	3.12	0.0001159
CYP27C1	1.64	3.12	1.11E-05
NR4A2	1.64	3.12	3.50E-06
TMEM14C	1.64	3.12	5.73E-07
UQCR10	1.64	3.12	1.99E-05
MYCBP	1.64	3.12	3.38E-06
UBE2I	1.64	3.12	1.33E-05
AZI2	1.64	3.12	1.73E-06
PDRG1	1.64	3.12	1.64E-06
LBFABP	1.64	3.12	2.89E-07
PDXDC1	1.64	3.11	0.0001362
RPL9	1.64	3.11	0.0001626
HELZ2	1.64	3.11	4.67E-06
MBD2	1.64	3.11	2.02E-06
GPX3	1.64	3.11	0.0001315
C8G	1.64	3.11	1.20E-06
MMP13	1.64	3.11	9.65E-07
LOC100858647	1.64	3.11	2.82E-06
MMP24	1.64	3.11	4.74E-05
TNIP1	1.64	3.11	0.0001005
NDUFA2	1.64	3.11	2.67E-07
MB	1.64	3.11	9.25E-08
LOC426155	1.64	3.11	1.89E-07
UQCRB	1.64	3.11	8.43E-07
CDK2AP1	1.64	3.11	3.59E-06
MINDY3	1.64	3.11	1.40E-06
CYP24A1	1.63	3.10	1.09E-06
UBC	1.63	3.10	1.39E-05
HEXIM1	1.63	3.10	2.12E-07
CIB1	1.63	3.10	8.86E-07
EXFABP	1.63	3.10	1.17E-07
UQCR11	1.63	3.10	1.93E-05
KCNMB2	1.63	3.09	1.32E-05
RPL21	1.63	3.09	0.0002114
LOC101750251	1.63	3.09	3.74E-07
SYNJ2BP	1.63	3.09	3.35E-09
CD226	1.63	3.09	0.0001989
PPP2R2B	1.63	3.09	8.58E-07
PFDN4	1.63	3.09	2.30E-06

JTB	1.63	3.09	2.54E-06
HIST1H110	1.63	3.09	0.0002348
CCAH221	1.63	3.09	4.52E-08
MTFP1	1.63	3.09	1.17E-06
RHOF	1.63	3.09	0.0001285
GUCD1	1.63	3.09	0.0004398
ELF3	1.62	3.08	3.85E-07
GLCCI1	1.62	3.08	0.001501
SEC11C	1.62	3.08	7.14E-06
QPCTL	1.62	3.08	5.46E-06
RSRP1	1.62	3.08	9.86E-06
LINGO1	1.62	3.08	7.18E-07
CHRNA5	1.62	3.08	0.0003311
MRPS36	1.62	3.08	2.15E-06
POLR2L	1.62	3.08	1.42E-06
LCT	1.62	3.08	0.0007661
LOC101750843	1.62	3.08	5.36E-06
SLC25A37	1.62	3.08	1.51E-06
IRAK2	1.62	3.07	0.0007552
PPDPF	1.62	3.07	5.16E-08
HIGD2B	1.62	3.07	1.39E-06
MINOS1	1.62	3.07	2.58E-07
GIMAP8L2	1.62	3.07	1.82E-06
DBNDD2	1.62	3.07	6.47E-07
TMEM57	1.62	3.07	0.001738
BF2	1.62	3.07	4.78E-06
EAF2	1.62	3.07	9.85E-06
TRAF5	1.62	3.06	3.62E-07
RPL18A	1.62	3.06	5.78E-06
PITX1	1.61	3.06	1.05E-07
HOXB1	1.61	3.06	7.56E-07
HORMAD2	1.61	3.06	1.42E-07
MTPN	1.61	3.06	2.09E-05
HIST2H3	1.61	3.06	3.84E-06
XPA	1.61	3.06	6.61E-08
SIIL	1.61	3.06	3.04E-06
TUSC2	1.61	3.05	2.66E-07
HENMT1	1.61	3.05	0.0003587
ITGB3	1.61	3.05	2.36E-06
HIST1H2A4	1.61	3.05	5.02E-06
RSRC2	1.61	3.05	0.001214
SLC26A9	1.61	3.05	1.86E-05
MGP	1.61	3.05	2.26E-08
PCBD2	1.61	3.04	4.47E-07
LRFN3	1.61	3.04	3.38E-07
RCAN1	1.60	3.04	0.0001764
DNLZ	1.60	3.04	2.11E-07
MMD	1.60	3.04	5.07E-07
CDKN2A	1.60	3.04	2.87E-09
RBP5	1.60	3.04	1.61E-09

RBM38	1.60	3.04	5.24E-06
TRABD2B	1.60	3.04	0.0007513
CNP	1.60	3.03	4.83E-09
LAMTOR5	1.60	3.03	1.65E-06
OMA1	1.60	3.03	3.70E-06
NFYB	1.60	3.03	8.78E-08
VPS26C	1.60	3.03	0.0002399
HACD1	1.60	3.03	2.74E-07
SPON2	1.60	3.03	6.21E-09
LOC770450	1.60	3.03	9.95E-09
CRISPLD2	1.60	3.03	0.0009397
HDGFL1	1.60	3.03	2.35E-07
ANKMY1	1.60	3.03	0.0008778
TACSTD2	1.60	3.02	2.00E-07
CXorf21	1.60	3.02	2.69E-05
IL13RA2	1.60	3.02	2.21E-06
TUBB1	1.60	3.02	7.33E-07
SELENOH	1.59	3.02	9.03E-08
HPS5	1.59	3.02	3.04E-07
SMKR1	1.59	3.02	1.58E-08
KCNJ15	1.59	3.02	7.19E-08
B2M	1.59	3.02	3.17E-06
NOS2	1.59	3.02	3.31E-07
MUSTN1	1.59	3.02	3.28E-07
PTBP2	1.59	3.01	0.0003944
ELOC	1.59	3.01	2.41E-08
UQCRQ	1.59	3.01	1.61E-07
C3H8ORF80	1.59	3.01	1.17E-07
IL1B	1.59	3.01	3.53E-06
GREM1	1.59	3.01	0.000109
RBP7	1.59	3.01	7.65E-08
PMAIP1	1.59	3.01	7.92E-07
ABCA12	1.59	3.00	3.30E-07
CACNA1B	1.59	3.00	3.89E-06
LOC419404	1.59	3.00	1.58E-07
ACYP2	1.59	3.00	1.84E-08
ARPP19	1.59	3.00	5.88E-06
LEAP2	1.58	3.00	1.77E-08
RAP1A	1.58	3.00	4.60E-08
NDUFAF8	1.58	3.00	2.22E-07
LOC100857266	1.58	3.00	2.47E-09
DLG3	1.58	3.00	2.08E-07
NCS1	1.58	2.99	6.57E-06
LOC769486	1.58	2.99	7.79E-08
SLC34A2	1.58	2.99	1.21E-06
PLAU	1.58	2.98	9.24E-06
LY6E	1.58	2.98	4.89E-08
YF5	1.57	2.98	5.31E-08
CYSTM1	1.57	2.98	5.95E-07
B3GNT7	1.57	2.98	4.09E-06

DNAH7	1.57	2.97	6.96E-06
LOC421584	1.57	2.97	3.02E-06
MUC2	1.57	2.97	7.90E-06
TOMM5	1.57	2.97	1.33E-07
ROPN1L	1.57	2.97	5.54E-10
RHOQ	1.57	2.97	4.63E-08
LOC101749175	1.57	2.97	2.85E-08
CHDH	1.57	2.97	3.74E-06
SRRM2	1.57	2.97	4.90E-08
TPSNRL	1.57	2.97	2.76E-07
CAMK2N1	1.57	2.97	7.68E-08
CNN1	1.57	2.96	4.14E-06
RASL11A	1.57	2.96	5.61E-07
HSBP1	1.57	2.96	3.97E-08
GCH1	1.57	2.96	1.67E-08
COX8A	1.57	2.96	2.98E-07
FGF8	1.57	2.96	1.33E-06
TAP2	1.56	2.96	7.09E-09
PEX3	1.56	2.96	2.13E-06
ERFE	1.56	2.96	3.50E-08
NUB1	1.56	2.96	3.38E-09
KIF2A	1.56	2.96	9.46E-09
RAB10L	1.56	2.96	3.31E-09
BCO1	1.56	2.96	8.23E-09
BAK1	1.56	2.95	6.32E-09
NRAP	1.56	2.95	0.002121
RNF213	1.56	2.95	6.35E-09
LOC101747405	1.56	2.95	4.64E-09
RWDD4	1.56	2.95	8.01E-08
PCBD1	1.56	2.95	8.37E-08
TAPBP	1.56	2.95	6.13E-09
IL6	1.56	2.95	2.22E-09
INHBB	1.56	2.95	3.97E-05
NT5C3B	1.56	2.95	9.31E-09
SAMHD1	1.56	2.95	1.34E-07
ACAD6L	1.56	2.94	4.57E-08
SST	1.56	2.94	2.15E-08
TRIM25	1.56	2.94	1.95E-10
C5H11ORF96	1.55	2.94	4.56E-08
DHX36	1.55	2.94	0.002047
CHD7	1.55	2.93	9.44E-05
ALB	1.55	2.93	6.72E-10
ASB5	1.55	2.93	4.20E-09
AVD	1.55	2.93	1.86E-06
ATOX1	1.55	2.93	4.48E-09
DDB2	1.55	2.93	0.0003182
BF1	1.55	2.92	1.00E-08
LOC101751989	1.55	2.92	3.72E-08
DTX3	1.54	2.92	3.39E-11
OSCP1	1.54	2.91	0.000458

SCT	1.54	2.91	0.0003723
GIMAP8L1	1.54	2.91	1.07E-08
PPFIBP1	1.54	2.91	9.64E-10
NDUFA1	1.54	2.91	3.16E-09
DESI2L	1.54	2.90	5.45E-08
YAE1D1	1.54	2.90	1.61E-07
ATF3	1.53	2.90	5.70E-09
MSI1	1.53	2.89	5.27E-06
CREG2	1.53	2.88	8.87E-10
AKAP5	1.53	2.88	7.51E-10
GET4	1.53	2.88	3.25E-07
LOC425214	1.53	2.88	2.75E-06
TAP1	1.53	2.88	1.71E-09
SRRM3	1.53	2.88	2.94E-07
OST4	1.52	2.88	2.53E-09
LOC415325	1.52	2.87	1.98E-06
SELE	1.52	2.87	2.94E-10
HIST1H2B7	1.52	2.87	1.91E-08
ADAR	1.52	2.87	2.51E-11
LYPLAL1	1.52	2.87	3.06E-08
PRDM12	1.52	2.87	7.09E-07
HBBA	1.52	2.87	4.18E-09
LYG2	1.52	2.86	2.42E-07
BTG4	1.52	2.86	2.70E-06
PDGFB	1.52	2.86	1.74E-07
PARP14L	1.51	2.86	1.63E-09
STOML1	1.51	2.85	8.45E-09
CRIP2	1.51	2.85	1.09E-10
TRAFD1	1.51	2.85	9.66E-11
MCOLN3	1.51	2.85	1.20E-07
GLRX	1.51	2.85	5.59E-11
ARAP2	1.51	2.85	1.50E-08
MXI1	1.51	2.85	1.35E-13
PCDH10	1.51	2.84	2.60E-07
MMP7	1.50	2.84	9.88E-08
RLN3	1.50	2.83	2.39E-08
HOXB3	1.50	2.83	7.24E-11
MMP23B	1.50	2.83	4.83E-10
HBA1	-1.84	-3.58	0.0004308
DNAJB4	-1.87	-3.64	0.001282
PCDH9	-1.92	-3.80	0.0007569
ORM1	-1.97	-3.91	0.003265
SETD1B	-1.97	-3.92	0.001429
RNASE4	-1.97	-3.92	0.002567
CENPE	-1.98	-3.96	2.98E-07
COL9A2	-2.00	-3.99	0.002746
SEMA6D	-2.00	-3.99	0.001603
SOX17	-2.00	-3.99	0.004248
NEB	-2.00	-3.99	0.004257
LOC395159	-2.00	-4.01	0.001446

BOD1L1	-2.02	-4.04	0.002061
ADH1C	-2.04	-4.12	0.003425
HOMER1	-2.04	-4.12	0.003422
BPTF	-2.06	-4.16	0.00494
BPI	-2.06	-4.18	0.003653
AGR2	-2.07	-4.19	0.004608
LOC100857474	-2.07	-4.20	0.003557
TSPAN8	-2.07	-4.20	0.002433
EEF1A2	-2.07	-4.21	0.005265
MATN4	-2.07	-4.21	0.000661
COBL	-2.08	-4.22	0.005344
BTAF1	-2.08	-4.23	0.0004487
RASL11B	-2.09	-4.26	0.0005997
KMT2A	-2.09	-4.26	0.002852
FGG	-2.09	-4.27	0.005272
SFTPA2	-2.10	-4.29	0.0001447
CENPF	-2.11	-4.31	3.04E-07
THBS4	-2.11	-4.31	0.003133
CKAP5	-2.11	-4.32	4.26E-05
MYOG	-2.11	-4.32	0.003348
CRYAB	-2.11	-4.32	0.002166
CHRDL1	-2.11	-4.32	0.003447
NKAIN4	-2.11	-4.33	0.00446
EPCAM	-2.12	-4.36	0.0006037
PALLD	-2.12	-4.36	0.003768
ADAMTS1	-2.12	-4.36	0.003736
ANKHD1	-2.12	-4.36	0.004954
CLEC3B	-2.13	-4.36	0.001097
EYA4	-2.13	-4.38	0.003781
F13A1	-2.13	-4.38	0.001221
FBLN5	-2.13	-4.38	0.0007031
TRIP12	-2.13	-4.38	0.00475
C3	-2.13	-4.39	0.003633
LOC407092	-2.13	-4.39	0.005307
GAS1	-2.14	-4.40	0.002587
FNDC1	-2.14	-4.40	0.003225
FSTL1	-2.14	-4.41	0.002604
FRMD3	-2.14	-4.41	0.002219
ALG12	-2.14	-4.42	0.002181
KIF5B	-2.15	-4.42	0.004316
HMOX1	-2.15	-4.44	0.001262
ENPP3	-2.15	-4.45	0.0008295
NEURL1	-2.16	-4.46	0.00329
CEP55	-2.16	-4.47	0.002167
RBM33	-2.16	-4.47	0.001047
SORBS1	-2.16	-4.48	0.001416
VEGFD	-2.17	-4.49	0.0009605
MYL1	-2.17	-4.50	0.00225
PLXNA4	-2.17	-4.50	0.004584
CNTRL	-2.17	-4.50	9.27E-05

XIRP1	-2.17	-4.50	0.0002206
AMOTL2	-2.17	-4.51	0.001319
LONRF1	-2.17	-4.51	0.003702
CBLB	-2.17	-4.51	0.0008274
COL6A1	-2.17	-4.51	0.001607
FN1	-2.17	-4.51	0.00174
KAT6B	-2.17	-4.51	0.004225
PDE5A	-2.17	-4.51	0.004175
PPP1R13B	-2.17	-4.52	0.002578
RUNX1	-2.17	-4.52	0.004478
NET1	-2.18	-4.52	0.002934
OGN	-2.18	-4.52	0.0003321
COL11A1	-2.18	-4.53	0.004254
DMD	-2.18	-4.53	0.004453
MYOT	-2.18	-4.53	0.0006038
LRIG3	-2.18	-4.53	0.0003946
KPNA1	-2.18	-4.54	0.002623
CHERP	-2.18	-4.54	0.004439
HSPB2	-2.18	-4.54	0.004827
IL4R	-2.19	-4.55	0.003822
ANKRD17	-2.19	-4.55	0.002322
LATS2	-2.19	-4.55	0.0001969
MRVI1	-2.19	-4.55	0.004277
NFXL1	-2.19	-4.55	0.001597
AP3D1	-2.19	-4.56	0.004216
PLXNB2	-2.19	-4.56	0.001849
THOC2	-2.19	-4.56	0.001842
TACC2	-2.19	-4.57	0.004385
FBN2	-2.19	-4.57	0.000205
SVIL	-2.19	-4.57	0.004525
DACH1	-2.19	-4.58	0.004814
FAM65B	-2.19	-4.58	0.00459
KDM3B	-2.19	-4.58	0.0001384
TNNC2	-2.19	-4.58	0.003107
VPS50	-2.19	-4.58	0.004823
ATAD5	-2.20	-4.58	0.00363
CDC5L	-2.20	-4.58	0.004113
EML4	-2.20	-4.58	0.002051
MED13L	-2.20	-4.59	0.003622
RNF19A	-2.20	-4.59	0.003937
ADAMTS3	-2.20	-4.59	0.004301
UTRN	-2.20	-4.59	0.0006506
TNMD	-2.20	-4.60	0.001018
POLR3A	-2.21	-4.62	0.001785
AMPD3	-2.21	-4.62	0.00454
PRICKLE1	-2.21	-4.63	0.002626
IFI30	-2.21	-4.64	0.005356
TGFBR3	-2.21	-4.64	0.002646
EVI2A	-2.21	-4.64	0.003767
GSTA3	-2.21	-4.64	0.0001202

MATN1	-2.21	-4.64	0.0002064
RDH10	-2.21	-4.64	0.004171
ALDH1A2	-2.22	-4.65	0.00063
LOC100858295	-2.22	-4.65	0.003728
UTP20	-2.22	-4.65	0.004594
HERC2	-2.22	-4.65	0.00409
KIDINS220	-2.22	-4.65	0.00243
DUSP4	-2.22	-4.66	0.000789
LARP1	-2.22	-4.66	0.001771
TUBGCP3	-2.22	-4.66	0.00033
RUBCNL	-2.22	-4.66	0.002615
APBB2	-2.22	-4.67	0.001683
MGAT4A	-2.22	-4.67	0.002194
COL11A2	-2.22	-4.67	0.002278
MYOF	-2.22	-4.67	0.001558
TFR2	-2.22	-4.67	0.003858
MED12	-2.23	-4.68	0.003474
PDE8A	-2.23	-4.68	0.004973
QSOX2	-2.23	-4.68	0.002551
TNNI2	-2.23	-4.68	0.0003173
ZMIZ1	-2.23	-4.68	0.002262
EPHA3	-2.23	-4.68	0.0002704
GPD2	-2.23	-4.68	0.003208
SMG1	-2.23	-4.68	0.0006173
UTP4	-2.23	-4.68	0.004689
ADAM28	-2.23	-4.69	3.57E-05
SUV39H1	-2.23	-4.69	0.002328
UPF1	-2.23	-4.69	0.0005854
GPR65	-2.23	-4.70	0.005355
SEL1L3	-2.23	-4.70	0.001733
HSD17B12	-2.23	-4.70	0.002688
NOL8	-2.23	-4.70	0.0007215
REXO5	-2.23	-4.70	0.004725
MARCO	-2.24	-4.71	0.001637
SON	-2.24	-4.71	0.0007973
ECT2	-2.24	-4.72	0.00274
EIF4G1	-2.24	-4.72	0.005217
SOX11	-2.24	-4.72	0.0008356
LCP1	-2.24	-4.73	0.0009889
SLC23A2	-2.24	-4.73	0.0004006
SYNPO2L	-2.24	-4.73	2.12E-05
LOC423766	-2.24	-4.73	0.002712
LRRC32	-2.24	-4.73	0.003541
OCM2	-2.24	-4.73	1.91E-05
RPN2	-2.24	-4.73	0.000234
TNPO1	-2.24	-4.73	0.0001987
USP47	-2.24	-4.73	0.002752
MEF2C	-2.24	-4.74	0.0005948
TBC1D1	-2.24	-4.74	0.005232
FAP	-2.25	-4.74	0.0006876

LOC415662	-2.25	-4.74	0.0007367
SFTPA1	-2.25	-4.74	3.05E-08
SHROOM2	-2.25	-4.74	0.001868
TES	-2.25	-4.74	0.002933
GGH	-2.25	-4.75	0.002467
HMMR	-2.25	-4.75	0.003267
PXYLP1	-2.25	-4.75	0.002634
RBM24	-2.25	-4.75	0.0002165
RBM25	-2.25	-4.75	0.004343
REEP3	-2.25	-4.75	0.001572
TNC	-2.25	-4.75	0.000119
COL22A1	-2.25	-4.75	0.004861
DHX37	-2.25	-4.75	0.000296
GDPD5	-2.25	-4.75	0.001163
RNF2	-2.25	-4.75	0.00221
PCNX1	-2.25	-4.76	0.001673
SH3PXD2A	-2.25	-4.76	0.001987
SOD3	-2.25	-4.76	0.0007696
KIF14	-2.25	-4.76	0.001168
KNL1	-2.25	-4.76	0.002408
LYPLA2	-2.25	-4.76	0.005011
PLXNA2	-2.25	-4.76	0.003072
VAT1L	-2.25	-4.76	0.004646
ATRX	-2.25	-4.77	0.001921
COL6A3	-2.25	-4.77	0.005356
FAM208A	-2.25	-4.77	0.004663
NMD3	-2.25	-4.77	0.004384
SPI1	-2.25	-4.77	0.0004251
SCAF8	-2.26	-4.78	0.003542
XPO5	-2.26	-4.78	0.003552
ZNF131	-2.26	-4.78	0.00437
CDT1	-2.26	-4.78	0.003688
CHD4	-2.26	-4.78	0.004958
NF1	-2.26	-4.78	0.00234
SLC25A25	-2.26	-4.78	0.003419
BMPR1A	-2.26	-4.79	0.001781
POLDIP2	-2.26	-4.79	0.00228
SDC3	-2.26	-4.79	0.0001455
TRIO	-2.26	-4.79	0.004155
CDC14B	-2.26	-4.79	0.002747
CRISPLD1	-2.26	-4.79	0.004613
CUL1	-2.26	-4.79	0.003937
LOC422171	-2.26	-4.79	0.001587
RTN4	-2.26	-4.79	0.0007836
ARHGAP24	-2.26	-4.80	0.0007332
CNOT6L	-2.26	-4.80	0.0006458
CPT1A	-2.26	-4.80	0.002662
ROBO2	-2.26	-4.80	0.002971
USP42	-2.26	-4.80	0.001468
C9H2ORF82	-2.26	-4.80	0.00273

DDX52	-2.26	-4.80	0.005017
ITGB2	-2.26	-4.80	0.0007835
SLC29A1	-2.26	-4.80	0.00172
IFITM5	-2.27	-4.81	3.02E-05
PKN1	-2.27	-4.81	0.00254
PTPN6	-2.27	-4.81	0.001074
PTPRM	-2.27	-4.81	0.004713
NPTX2	-2.27	-4.81	0.002864
GINS1	-2.27	-4.82	0.002974
ZMYND11	-2.27	-4.82	0.001301
CHM	-2.27	-4.82	0.001071
FOCAD	-2.27	-4.82	0.003576
CDK17	-2.27	-4.83	0.00532
DYM	-2.27	-4.83	0.001353
EPHB2	-2.27	-4.83	0.005388
GLB1	-2.27	-4.83	0.002761
SLC25A30	-2.27	-4.83	0.004649
ABHD2	-2.27	-4.84	0.00512
GOLGA2	-2.27	-4.84	0.00211
PRPF39	-2.27	-4.84	0.005298
CHD9	-2.28	-4.84	0.002623
KIAA0430	-2.28	-4.84	0.0009557
LRCH4	-2.28	-4.84	0.004296
PLA2G4A	-2.28	-4.84	0.005212
FAM168B	-2.28	-4.85	0.002279
POLR1A	-2.28	-4.85	0.001284
PTH1R	-2.28	-4.85	0.0024
CA2	-2.28	-4.85	0.00159
CHRNA1	-2.28	-4.85	0.001157
FLRT3	-2.28	-4.85	0.001618
KIAA1107	-2.28	-4.85	0.001197
NAV3	-2.28	-4.85	0.002277
SENP6	-2.28	-4.85	0.004398
APLP2	-2.28	-4.86	0.0002139
CEP170	-2.28	-4.86	0.0005303
ID2	-2.28	-4.86	0.003469
MAP2K3	-2.28	-4.86	0.002142
RPAP1	-2.28	-4.86	0.002559
ZFHX4	-2.28	-4.86	0.0009711
CPEB1	-2.28	-4.86	0.001433
HSPG2	-2.28	-4.86	0.001057
PHIP	-2.28	-4.86	0.002404
SHROOM1	-2.28	-4.86	0.0009464
SPEN	-2.28	-4.86	0.002699
COL2A1	-2.28	-4.87	5.02E-05
DDX24	-2.28	-4.87	0.001411
EMB	-2.28	-4.87	0.002863
NRK	-2.28	-4.87	0.00188
SLC2A14	-2.28	-4.87	0.003653
WIPF1	-2.28	-4.87	0.0002211

AARS	-2.28	-4.87	0.005374
CAVIN4	-2.28	-4.87	0.0008137
DKK1	-2.28	-4.87	6.11E-05
GNPTAB	-2.28	-4.87	0.005196
IGF2BP3	-2.28	-4.87	0.004143
ITGAV	-2.28	-4.87	0.001956
LOC100859072	-2.28	-4.87	0.003478
TUBB	-2.28	-4.87	0.0003815
FOXP2	-2.29	-4.88	0.002465
HAPLN1	-2.29	-4.88	0.0003638
LOX	-2.29	-4.88	0.0002131
MYCBP2	-2.29	-4.88	0.001529
MYH1B	-2.29	-4.88	0.001182
PRR5L	-2.29	-4.88	0.003251
WWC2	-2.29	-4.88	0.004992
CTDSPL2	-2.29	-4.88	0.004261
EGR1	-2.29	-4.88	0.0004616
KIAA0355	-2.29	-4.88	0.0001604
METTL13	-2.29	-4.88	0.003113
SLC7A3	-2.29	-4.88	0.0002882
CEP89	-2.29	-4.89	0.001483
DHX8	-2.29	-4.89	0.001763
DTL	-2.29	-4.89	0.002243
MAP3K1	-2.29	-4.89	0.00257
RASGEF1B	-2.29	-4.89	0.0005998
SEC31A	-2.29	-4.89	0.005093
CASP8AP2	-2.29	-4.89	0.004822
DDR2	-2.29	-4.89	0.0008438
ERBIN	-2.29	-4.89	0.004549
FAM105A	-2.29	-4.89	0.003845
HMG20A	-2.29	-4.89	0.0009836
LATS1	-2.29	-4.89	0.003799
TMEM131	-2.29	-4.89	0.00315
GOLGB1	-2.29	-4.90	0.0007749
MT4L	-2.29	-4.90	0.001059
PUM1	-2.29	-4.90	0.0005075
SULF1	-2.29	-4.90	0.0002089
TSHZ1	-2.29	-4.90	0.001547
TTYH2	-2.29	-4.90	0.0005698
ADAM12	-2.29	-4.90	0.0004477
AGAP1	-2.29	-4.90	0.004615
ANKRD1	-2.29	-4.90	0.0005512
COL1A2	-2.29	-4.90	0.0002832
SLC10A7	-2.29	-4.90	0.002077
TOMM34	-2.29	-4.90	0.004006
FECH	-2.30	-4.91	0.003314
LOC416530	-2.30	-4.91	0.001903
RAI14	-2.30	-4.91	0.000127
TOB2	-2.30	-4.91	0.001859
USP24	-2.30	-4.91	0.00115

DDX54	-2.30	-4.92	0.004781
EML1	-2.30	-4.92	0.004644
ZNF106	-2.30	-4.92	0.005078
BBOX1	-2.30	-4.92	0.003464
KIAA1551	-2.30	-4.92	0.005112
TOX3	-2.30	-4.92	0.0003727
ATP13A3	-2.30	-4.93	0.004241
DMXL2	-2.30	-4.93	0.002061
KMT2C	-2.30	-4.93	0.0008145
LPCAT2	-2.30	-4.93	0.002668
OSBPL1A	-2.30	-4.93	0.002437
LOC100857946	-2.30	-4.93	0.0008188
SLC22A23	-2.30	-4.93	0.002566
TGFBRAP1	-2.30	-4.93	0.003467
YAP1	-2.30	-4.93	0.00137
ZNF423	-2.30	-4.93	0.003403
ANKRD52	-2.30	-4.94	0.00163
ARHGEF1	-2.30	-4.94	0.0004275
CAMSAP3	-2.30	-4.94	0.004003
DIO2	-2.30	-4.94	0.001026
FBLN1	-2.30	-4.94	0.002632
INO80	-2.30	-4.94	0.002696
LOC424111	-2.30	-4.94	0.002046
MYH11	-2.30	-4.94	0.003123
NFIA	-2.30	-4.94	0.002769
STARD13	-2.30	-4.94	0.003468
FAT3	-2.31	-4.94	0.001737
LOC101748723	-2.31	-4.94	0.0007245
PCDH11X	-2.31	-4.94	0.0001888
PROSER2	-2.31	-4.94	0.001492
TNRC6B	-2.31	-4.94	0.005129
ZCCHC6	-2.31	-4.94	0.003895
ZFRL1	-2.31	-4.94	0.003413
ACLY	-2.31	-4.95	0.0003127
AKAP9	-2.31	-4.95	0.00197
AP2B1	-2.31	-4.95	0.001612
CNOT1	-2.31	-4.95	0.004608
EIF4ENIF1	-2.31	-4.95	0.001591
FAT4	-2.31	-4.95	0.001566
LOC101752174	-2.31	-4.95	0.0008169
PLXNA1	-2.31	-4.95	0.00137
ST5	-2.31	-4.95	0.0007943
UBR4	-2.31	-4.95	0.002205
ARHGAP29	-2.31	-4.95	0.0002155
EIF3A	-2.31	-4.95	0.0006651
SERPINF1	-2.31	-4.95	0.004167
TPP2	-2.31	-4.95	0.0006029
FLRT2	-2.31	-4.96	0.002317
KPNA3	-2.31	-4.96	0.003588
MTCL1	-2.31	-4.96	0.0006586

UNG	-2.31	-4.96	0.004183
VPS13A	-2.31	-4.96	0.002659
ZFC3H1	-2.31	-4.96	0.001554
BRWD1	-2.31	-4.96	0.001391
CACNA2D1	-2.31	-4.96	0.0008971
RNPS1	-2.31	-4.96	0.003647
SREBF2	-2.31	-4.96	0.00246
CASQ2	-2.31	-4.97	0.0004805
KDM5B	-2.31	-4.97	0.002173
RBM10	-2.31	-4.97	1.93E-05
SVEP1	-2.31	-4.97	0.0003884
TJP1	-2.31	-4.97	0.002916
ANLN	-2.31	-4.98	0.002913
BHMT	-2.31	-4.98	0.0001637
FBN1	-2.31	-4.98	0.001334
ZBTB41	-2.31	-4.98	0.003916
ZMYM4	-2.31	-4.98	0.0003696
ZNF507	-2.31	-4.98	0.00197
CELF1	-2.32	-4.98	0.0005871
PIEZO2	-2.32	-4.98	0.002018
TNNT3	-2.32	-4.98	7.40E-05
DIP2B	-2.32	-4.99	0.00382
EFR3A	-2.32	-4.99	0.003002
FAT1	-2.32	-4.99	0.001082
KCNQ1	-2.32	-4.99	0.003919
MED16	-2.32	-4.99	0.004779
NCAM1	-2.32	-4.99	0.0003641
OSBPL8	-2.32	-4.99	0.003003
PLA2G10	-2.32	-4.99	0.001131
ZBTB49	-2.32	-4.99	0.004986
CRTC1	-2.32	-4.99	0.002076
KANSL1L	-2.32	-4.99	0.0006747
KIAA1217	-2.32	-4.99	0.001718
LHX8	-2.32	-4.99	0.001008
PICALM	-2.32	-4.99	0.003575
THBS2	-2.32	-4.99	1.23E-05
TSEN54	-2.32	-4.99	0.002708
ZSWIM8	-2.32	-4.99	0.004986
FANCB	-2.32	-5.00	0.001617
GAPVD1	-2.32	-5.00	0.003257
MCM10	-2.32	-5.00	0.0001742
NEK1	-2.32	-5.00	0.002139
PPIP5K2	-2.32	-5.00	0.00203
SLC6A6	-2.32	-5.00	0.003452
TICRR	-2.32	-5.00	7.05E-05
TLL1	-2.32	-5.00	0.00164
TMEM132C	-2.32	-5.00	0.00185
WDFY4	-2.32	-5.00	0.00438
DDX21	-2.32	-5.00	0.004407
IFT88	-2.32	-5.00	0.001825

RBM27	-2.32	-5.00	0.0003824
SRSF7	-2.32	-5.00	0.001278
ZMYM2	-2.32	-5.00	0.002218
DOP1A	-2.32	-5.01	0.002774
PLCB1	-2.32	-5.01	0.0002952
SNRNP200	-2.32	-5.01	0.0003717
TENM3	-2.32	-5.01	0.003817
UTP6	-2.32	-5.01	0.002528
ZNF148	-2.32	-5.01	0.0006291
EMC1	-2.33	-5.01	0.0004942
EPHA2	-2.33	-5.01	6.41E-05
LIN54	-2.33	-5.01	0.002372
MAP1B	-2.33	-5.01	0.0002457
MARS	-2.33	-5.01	0.001872
RIN2	-2.33	-5.01	0.0003192
DGKD	-2.33	-5.02	0.001473
MYH1E	-2.33	-5.02	0.0005231
SLC47A1	-2.33	-5.02	0.001069
WDR89	-2.33	-5.02	0.001542
ABCB6	-2.33	-5.03	0.002799
CDYL	-2.33	-5.03	0.003415
COL24A1	-2.33	-5.03	1.95E-05
DDAH1	-2.33	-5.03	0.005043
NUDT4	-2.33	-5.03	0.001173
PDLIM5	-2.33	-5.03	0.0004561
SHQ1	-2.33	-5.03	0.0004154
STAC	-2.33	-5.03	0.0002506
TBC1D4	-2.33	-5.03	0.003936
NSD1	-2.33	-5.03	0.004431
RIOK3	-2.33	-5.03	0.00225
SLC27A4	-2.33	-5.03	0.002511
SRBD1	-2.33	-5.03	0.001642
ST8SIA6	-2.33	-5.03	0.001477
BAZ2B	-2.33	-5.04	0.0007696
CBL	-2.33	-5.04	0.005205
CP	-2.33	-5.04	1.53E-05
MKL1	-2.33	-5.04	0.003188
PARD3B	-2.33	-5.04	0.005266
PRG4	-2.33	-5.04	0.0007475
SPECC1L	-2.33	-5.04	0.004704
ZBTB38	-2.33	-5.04	0.001269
FAM210B	-2.33	-5.04	0.003247
APPL1	-2.34	-5.05	0.001523
BRCA1	-2.34	-5.05	0.000101
HNRNPM	-2.34	-5.05	0.0002865
MAP7D3	-2.34	-5.05	0.0003956
PAICS	-2.34	-5.05	0.002761
PBRM1	-2.34	-5.05	0.002158
PRRC2C	-2.34	-5.05	0.0001192
SBNO1	-2.34	-5.05	0.004017

SEC61A2	-2.34	-5.05	0.0007504
SNTB1	-2.34	-5.05	0.000698
ZBTB11	-2.34	-5.05	0.0002373
ACTR3B	-2.34	-5.05	0.003654
CPZ	-2.34	-5.05	0.000742
ITGBL1	-2.34	-5.05	0.0001742
KDM5A	-2.34	-5.05	0.001141
NUP153	-2.34	-5.05	0.0003332
SPRY1	-2.34	-5.05	0.0003033
AKAP13	-2.34	-5.06	0.005288
DCAF13	-2.34	-5.06	0.003938
PRPF40A	-2.34	-5.06	0.0003665
RECK	-2.34	-5.06	0.001845
BCL11A	-2.34	-5.07	0.0005702
DAAM2	-2.34	-5.07	0.0008936
KDM6A	-2.34	-5.07	0.003286
MPHOSPH9	-2.34	-5.07	0.004653
SPAG9	-2.34	-5.07	0.001038
ST8SIA5	-2.34	-5.07	0.004478
VPS39	-2.34	-5.07	0.004517
XRN1	-2.34	-5.07	0.0001246
ADCY5	-2.34	-5.07	0.004555
MED23	-2.34	-5.07	0.001228
RELN	-2.34	-5.07	0.0001054
SLBP	-2.34	-5.07	0.0002398
ABCC1	-2.34	-5.08	0.000425
ETNK1	-2.34	-5.08	0.002877
MTM1	-2.34	-5.08	0.001841
ZNF462	-2.34	-5.08	0.003873
CSRNP1	-2.35	-5.08	0.002161
CTSD	-2.35	-5.08	0.0001819
FSCN1	-2.35	-5.08	0.0007055
MAP1A	-2.35	-5.08	0.0002701
PCDH7	-2.35	-5.08	0.0002139
SLC25A29	-2.35	-5.08	0.005206
ACOT2L	-2.35	-5.09	0.001151
AOAH	-2.35	-5.09	0.004385
CCDC102A	-2.35	-5.09	0.003527
MTBP	-2.35	-5.09	0.002054
PAPD7	-2.35	-5.09	0.001755
PDE10A	-2.35	-5.09	0.003192
PDE4DIP	-2.35	-5.09	0.002071
UACA	-2.35	-5.09	0.0006379
ZFX	-2.35	-5.09	0.0003363
ALS2	-2.35	-5.09	0.004744
MDN1	-2.35	-5.09	0.0004074
MICAL3	-2.35	-5.09	0.002824
PDXP	-2.35	-5.09	0.001044
ADGRA3	-2.35	-5.10	0.00138
APBB1IP	-2.35	-5.10	0.004208

ATF6B	-2.35	-5.10	0.002861
BIN1	-2.35	-5.10	0.000951
CDC42BPA	-2.35	-5.10	0.001629
IPP	-2.35	-5.10	0.003606
MYO9B	-2.35	-5.10	4.44E-05
RBSN	-2.35	-5.10	0.0007511
SPSB2	-2.35	-5.10	0.004537
WTIP	-2.35	-5.10	0.0009919
COL12A1	-2.35	-5.11	0.005223
DCAF1	-2.35	-5.11	0.0007061
GPR68	-2.35	-5.11	0.0001809
LOC100859715	-2.35	-5.11	1.10E-07
TMEM47	-2.35	-5.11	0.004254
TPM1	-2.35	-5.11	0.005022
UVSSA	-2.35	-5.11	0.004177
ADAMTS5	-2.35	-5.11	0.0007624
ERCC6L2	-2.35	-5.11	0.0009666
HSF2	-2.35	-5.11	0.0008542
INO80D	-2.35	-5.11	0.004723
NOL6	-2.35	-5.11	0.001325
CCSER2	-2.36	-5.12	0.001157
TAC1	-2.36	-5.12	0.0004886
TRPM7	-2.36	-5.12	0.0006089
CMTM4	-2.36	-5.12	7.15E-05
E2F7	-2.36	-5.12	0.0009417
TSHZ2	-2.36	-5.12	0.002654
SEC24D	-2.36	-5.13	0.0003356
USP37	-2.36	-5.13	0.0004583
ARMC4	-2.36	-5.13	0.001283
CCDC88A	-2.36	-5.13	0.0005387
CCT3	-2.36	-5.13	0.003826
CDC7	-2.36	-5.13	0.003621
CDH6	-2.36	-5.13	0.005032
DNA2	-2.36	-5.13	0.0006865
KIF20B	-2.36	-5.13	0.0001032
TRAPPC11	-2.36	-5.13	0.002345
UAP1	-2.36	-5.13	0.004381
ACTN2	-2.36	-5.14	6.59E-08
ALDH1L2	-2.36	-5.14	0.005014
BIRC6	-2.36	-5.14	0.0001404
HECTD4	-2.36	-5.14	0.0001679
IDE	-2.36	-5.14	0.0001804
KCNJ2	-2.36	-5.14	0.001383
MCM6	-2.36	-5.14	0.003058
PWP1	-2.36	-5.14	0.00348
ATXN3	-2.36	-5.15	0.004911
COL15A1	-2.36	-5.15	0.002063
CRKL	-2.36	-5.15	0.00119
CTDP1	-2.36	-5.15	0.0003101
EP400	-2.36	-5.15	0.001024

FBXO39	-2.36	-5.15	0.00189
ITGA8	-2.36	-5.15	0.0002571
MYO10L	-2.36	-5.15	0.0006604
NCDN	-2.36	-5.15	0.0005336
PPM1K	-2.36	-5.15	0.0002811
SMPD3	-2.36	-5.15	0.0006883
SPON1	-2.36	-5.15	0.000625
TMEM186	-2.36	-5.15	0.002879
ZGRF1	-2.36	-5.15	0.002381
ANKLE2	-2.37	-5.15	0.001439
ATP2B2	-2.37	-5.15	0.0008299
DDX28	-2.37	-5.15	0.002202
MSTO1	-2.37	-5.15	0.001836
NT5DC3	-2.37	-5.15	0.002468
VWA8	-2.37	-5.15	0.002569
CCNE1	-2.37	-5.16	0.00369
CSE1L	-2.37	-5.16	0.004539
RASA3	-2.37	-5.16	0.0001066
SMARCA4	-2.37	-5.16	0.0005815
EFTUD2	-2.37	-5.16	0.002507
FAM110B	-2.37	-5.16	0.003166
LOC776067	-2.37	-5.16	0.001206
PAG1	-2.37	-5.16	0.000503
CTNNA1	-2.37	-5.17	0.001392
DNER	-2.37	-5.17	0.0003954
KRT7	-2.37	-5.17	0.003984
LRRC58	-2.37	-5.17	0.001211
DDX20	-2.37	-5.18	0.003823
MAN1A2	-2.37	-5.18	0.003696
MEGF6L	-2.37	-5.18	7.48E-05
NOTCH2	-2.37	-5.18	0.0004662
TRAPPC8	-2.37	-5.18	0.0005127
FGFR2	-2.37	-5.18	1.12E-05
GOPC	-2.37	-5.18	0.003698
LOC396224	-2.37	-5.18	0.004918
PTPRR	-2.37	-5.18	0.004903
TOPORS	-2.37	-5.18	0.002171
CLIP1	-2.38	-5.19	0.001881
CNKSR3	-2.38	-5.19	8.33E-05
LRRC8C	-2.38	-5.19	0.002532
NEMF	-2.38	-5.19	0.002713
NID1	-2.38	-5.19	0.0006569
RAPH1	-2.38	-5.19	0.0002753
RBMS3	-2.38	-5.19	0.0002487
DKC1	-2.38	-5.19	0.0007205
ITGB1	-2.38	-5.19	0.003391
PPP6R3	-2.38	-5.19	0.0004021
TOGARAM1	-2.38	-5.19	0.002337
ALCAM	-2.38	-5.20	0.001259
CASK	-2.38	-5.20	0.0002491

CENPU	-2.38	-5.20	0.00446
EDN2	-2.38	-5.20	1.10E-06
GOLGA4	-2.38	-5.20	0.001438
KPNA5	-2.38	-5.20	0.003613
LTA4H	-2.38	-5.20	0.00162
STN1	-2.38	-5.20	0.004546
ZDHHC17	-2.38	-5.20	0.002031
AMFR	-2.38	-5.21	0.003322
ATG16L1	-2.38	-5.21	0.00204
CBX8	-2.38	-5.21	0.003279
DHX57	-2.38	-5.21	0.003064
THOP1	-2.38	-5.21	0.0007305
AGGF1	-2.38	-5.21	0.003326
ANKRD46	-2.38	-5.21	0.002367
GSC	-2.38	-5.21	4.60E-05
KRT14	-2.38	-5.21	0.000183
PPM1E	-2.38	-5.21	0.002511
BRD1	-2.38	-5.22	0.0005572
DDX11	-2.38	-5.22	0.0006809
DPYD	-2.38	-5.22	0.003001
EIF5B	-2.38	-5.22	0.002588
FAM199X	-2.38	-5.22	0.002262
HIP1R	-2.38	-5.22	0.00241
IPO9	-2.38	-5.22	0.002154
ADCY2	-2.39	-5.22	0.0003138
B4GALT6	-2.39	-5.22	0.001458
ELMO1	-2.39	-5.22	0.002236
FBN3	-2.39	-5.22	4.44E-06
FLII	-2.39	-5.22	0.003314
GULP1	-2.39	-5.22	9.52E-05
KCNAB3	-2.39	-5.22	0.004669
KDM1A	-2.39	-5.22	0.001538
LOC415456	-2.39	-5.22	0.000629
NOP2	-2.39	-5.22	0.002773
PPP4R1	-2.39	-5.22	0.001171
SEC23A	-2.39	-5.22	0.001907
C2CD5	-2.39	-5.23	0.000182
KHDRBS1	-2.39	-5.23	0.0008686
VPS36	-2.39	-5.23	0.0007096
CREB3L2	-2.39	-5.24	0.002838
SLC39A6	-2.39	-5.24	0.0008055
TUBA1A	-2.39	-5.24	0.002127
ZMYND8	-2.39	-5.24	0.0004552
CAMTA2	-2.39	-5.24	0.00139
COL14A1	-2.39	-5.24	0.004282
G3BP1	-2.39	-5.24	0.0004905
HBS1L	-2.39	-5.24	0.0003932
LPP	-2.39	-5.24	3.66E-05
SART3	-2.39	-5.24	0.0001456
TARSL2	-2.39	-5.24	0.0001897

CWF19L2	-2.39	-5.25	0.001377
GPAM	-2.39	-5.25	0.0007673
HDAC4	-2.39	-5.25	0.0002457
NOC2L	-2.39	-5.25	0.001551
RABGAP1L	-2.39	-5.25	0.004073
ZC3H7A	-2.39	-5.25	0.001184
ATXN2	-2.39	-5.25	0.0008526
NCKAP1	-2.39	-5.25	0.003041
POLA1	-2.39	-5.25	0.0001496
RASA2	-2.39	-5.26	0.0002484
TMEM214	-2.39	-5.26	0.001222
TRPS1	-2.39	-5.26	0.0005249
ALDH9A1	-2.40	-5.27	0.001453
ARID4A	-2.40	-5.27	0.002037
ATP1B1	-2.40	-5.27	0.004737
C4H20ORF194	-2.40	-5.27	0.001007
CACFD1	-2.40	-5.27	0.003596
ERCC6L	-2.40	-5.27	0.0001013
KCTD10	-2.40	-5.27	0.002602
LARP6	-2.40	-5.27	0.00352
LRP5	-2.40	-5.27	0.00103
NCKIPSD	-2.40	-5.27	0.001546
NOLC1	-2.40	-5.27	0.0001794
RAB3IP	-2.40	-5.27	1.29E-05
ATP11B	-2.40	-5.27	0.0001979
CDC73	-2.40	-5.27	0.0003281
CFI	-2.40	-5.27	0.003244
MMS19	-2.40	-5.27	0.0001655
NTF3	-2.40	-5.27	0.0005903
LMINA	-2.40	-5.28	0.003985
LOC427439	-2.40	-5.28	0.0004221
MTMR6	-2.40	-5.28	9.07E-05
NLE1	-2.40	-5.28	0.0002135
POM121	-2.40	-5.28	0.001035
TBC1D2B	-2.40	-5.28	0.00138
BMS1	-2.40	-5.28	0.0001301
CEP192	-2.40	-5.28	0.0001859
DOCK6	-2.40	-5.28	0.0001012
FBXO25	-2.40	-5.28	0.00537
LOC415641	-2.40	-5.28	0.0008739
P4HB	-2.40	-5.28	1.75E-05
RETSAT	-2.40	-5.28	0.001973
TCERG1	-2.40	-5.28	0.003313
LDHA	-2.40	-5.29	0.004012
LMO1	-2.40	-5.29	0.001037
MTO1	-2.40	-5.29	0.003523
MXRA5	-2.40	-5.29	0.0004827
RAD18	-2.40	-5.29	0.001948
RPS6KA5	-2.40	-5.29	0.003169
SRGAP1	-2.40	-5.29	0.0002355

STIL	-2.40	-5.29	0.0001887
TMEM209	-2.40	-5.29	0.0003902
CEP120	-2.40	-5.30	0.0001783
CXCR4	-2.40	-5.30	0.0002451
MAPK1	-2.40	-5.30	0.002711
PIGU	-2.40	-5.30	0.002603
RAPGEF2	-2.40	-5.30	0.0002061
SIPA1L2	-2.40	-5.30	0.00226
SPOPL	-2.40	-5.30	0.000425
SPTBN1	-2.40	-5.30	0.0002436
SYNE1	-2.40	-5.30	9.63E-05
TRIB2	-2.40	-5.30	0.0007247
ARNT2	-2.41	-5.30	0.004428
GNA11	-2.41	-5.30	0.0003386
HOXA9	-2.41	-5.30	0.0005334
MATR3	-2.41	-5.30	0.003631
NAA25	-2.41	-5.30	0.0005799
SNAI1	-2.41	-5.30	0.001067
TOP3B	-2.41	-5.30	0.0002119
VPS54	-2.41	-5.30	0.001282
ANKFY1	-2.41	-5.31	0.0002342
GART	-2.41	-5.31	0.003751
GLUD1	-2.41	-5.31	0.001187
AGO1	-2.41	-5.31	0.00143
CABIN1	-2.41	-5.31	0.003469
DDI1	-2.41	-5.31	0.003688
KPNB1	-2.41	-5.31	3.76E-05
NAT10	-2.41	-5.31	6.71E-05
SELENOI	-2.41	-5.31	0.0009493
SMOC2	-2.41	-5.31	0.0007563
TLE4Z1	-2.41	-5.31	0.002241
P3H1	-2.41	-5.32	0.003176
WDFY3	-2.41	-5.32	0.0003101
ANKRD26	-2.41	-5.33	0.0001627
CAMSAP1	-2.41	-5.33	5.01E-05
NRBP1	-2.41	-5.33	0.0007901
PPP2R3B	-2.41	-5.33	0.0002205
RSBN1L	-2.41	-5.33	0.001161
ZFR2	-2.41	-5.33	0.0001958
ZRANB1	-2.41	-5.33	0.001747
APOO	-2.41	-5.33	0.003911
CHD6	-2.41	-5.33	0.001212
LINS1	-2.41	-5.33	0.001926
NES	-2.41	-5.33	1.23E-05
SINHCAF	-2.41	-5.33	0.005066
SMYD5	-2.41	-5.33	0.001645
BRD4	-2.42	-5.34	0.003528
CUL4B	-2.42	-5.34	0.001389
MACF1	-2.42	-5.34	0.0003642
MGA	-2.42	-5.34	0.0002165

MYO19	-2.42	-5.34	0.003366
STX8	-2.42	-5.34	0.005205
ARID4B	-2.42	-5.35	0.0003796
EEPD1	-2.42	-5.35	0.001466
HDX	-2.42	-5.35	0.003632
CALD1	-2.42	-5.35	0.0006848
MYOD1	-2.42	-5.35	9.33E-09
MYOM3	-2.42	-5.35	3.67E-05
PTX3	-2.42	-5.35	8.81E-05
ST3GAL5	-2.42	-5.35	0.001583
USP19	-2.42	-5.35	0.0007982
USPL1	-2.42	-5.35	0.0003214
GPS1	-2.42	-5.36	0.001724
LARP4B	-2.42	-5.36	0.001376
MIA2	-2.42	-5.36	0.0003121
PALB2	-2.42	-5.36	0.003272
PAXIP1	-2.42	-5.36	0.003938
PDGFRA	-2.42	-5.36	6.45E-06
PRKD3	-2.42	-5.36	0.002517
SOWAHC	-2.42	-5.36	0.0004681
ZNF516	-2.42	-5.36	0.001598
BICC1	-2.42	-5.36	4.42E-05
C14H7orf26	-2.42	-5.36	0.0009659
CLDN1	-2.42	-5.36	0.001068
DEPDC5	-2.42	-5.36	0.0003423
FAR2	-2.42	-5.36	0.002015
JMJD1C	-2.42	-5.36	4.67E-05
PIM3	-2.42	-5.36	0.001162
TUBGCP4	-2.42	-5.36	0.00163
ALG3	-2.43	-5.37	0.001445
CWC22	-2.43	-5.37	0.002299
HELLS	-2.43	-5.37	0.0002222
PCDH19	-2.43	-5.37	0.001185
PRR14L	-2.43	-5.37	0.0001305
SLC4A7	-2.43	-5.37	0.0004225
UQCRC2	-2.43	-5.37	0.0008697
CCT5	-2.43	-5.38	0.004714
DDX47	-2.43	-5.38	0.004329
MAN2A1	-2.43	-5.38	0.00108
NCKAP5	-2.43	-5.38	0.0001357
ADGRL2	-2.43	-5.38	0.0004064
INF2	-2.43	-5.38	0.0004468
LOC416257	-2.43	-5.38	0.002194
NUDT16L1	-2.43	-5.38	0.002277
ROCK2	-2.43	-5.38	0.000444
STEAP1	-2.43	-5.38	8.86E-05
DPP9	-2.43	-5.39	0.001537
DUS2	-2.43	-5.39	0.003202
EXOC2	-2.43	-5.39	0.0002599
HERC1	-2.43	-5.39	0.0003755

LOC426820	-2.43	-5.39	1.64E-05
NRCAM	-2.43	-5.39	0.0001287
OSGIN2	-2.43	-5.39	0.004352
CAD	-2.43	-5.40	0.0001314
CCNL2	-2.43	-5.40	0.002248
GPR176	-2.43	-5.40	0.00499
HADHB	-2.43	-5.40	0.003751
HYOU1	-2.43	-5.40	0.00109
IQGAP2	-2.43	-5.40	0.0003687
PANK1	-2.43	-5.40	0.005062
TSR1	-2.43	-5.40	0.0001721
DCLK1	-2.43	-5.40	0.0003713
EGFR	-2.43	-5.40	0.0001943
EXOC1	-2.43	-5.40	8.86E-05
GEMIN4	-2.43	-5.40	0.001496
GPR161	-2.43	-5.40	0.001722
NEFM	-2.43	-5.40	7.34E-07
NUP98	-2.43	-5.40	0.003291
WDR12	-2.43	-5.40	0.003989
ASCC2	-2.44	-5.41	0.00395
CCT7	-2.44	-5.41	0.0006058
CYFIP2	-2.44	-5.41	0.0007653
MAP3K4	-2.44	-5.41	0.005146
SETMAR	-2.44	-5.41	0.001186
CAPN7	-2.44	-5.41	0.001783
DNAJC3	-2.44	-5.41	0.003648
LOC421241	-2.44	-5.41	0.001342
MCC	-2.44	-5.41	0.0001787
AHCY	-2.44	-5.42	0.004436
DNM1	-2.44	-5.42	0.001441
IBTK	-2.44	-5.42	0.0007535
LMBR1	-2.44	-5.42	0.004874
NNT	-2.44	-5.42	0.001227
RNF20	-2.44	-5.42	0.001532
RRP12	-2.44	-5.42	1.63E-05
CABLES1	-2.44	-5.43	0.0009614
CLDN5	-2.44	-5.43	0.002075
ANKZF1	-2.44	-5.43	0.0004745
ASH1L	-2.44	-5.43	0.001923
BBS9	-2.44	-5.43	0.0002172
CCNT2	-2.44	-5.43	0.00421
CDAN1	-2.44	-5.43	0.002083
CHADL	-2.44	-5.43	0.001158
DHX30	-2.44	-5.43	0.003037
FILIP1L	-2.44	-5.43	8.20E-05
MARCH7	-2.44	-5.43	0.00519
OLFML2B	-2.44	-5.43	0.001199
PARP4	-2.44	-5.43	0.0001515
VASN	-2.44	-5.43	0.004741
FBXO11	-2.44	-5.44	0.003215

HAUS6	-2.44	-5.44	0.0005648
JARID2	-2.44	-5.44	0.0006122
MCM4	-2.44	-5.44	0.0003798
TTC37	-2.44	-5.44	0.0008981
AATF	-2.45	-5.45	2.68E-05
BORA	-2.45	-5.45	0.0007182
EMILIN3	-2.45	-5.45	3.92E-07
KANK1	-2.45	-5.45	0.001374
LMNB2	-2.45	-5.45	0.0003122
N4BP2	-2.45	-5.45	0.0007074
PAPSS1	-2.45	-5.45	0.000528
RALGAPA1	-2.45	-5.45	0.001692
CYB5R4	-2.45	-5.45	0.001592
SRP72	-2.45	-5.45	0.004743
SUZ12	-2.45	-5.45	6.93E-05
FAM160B1	-2.45	-5.46	0.0001042
NUFIP2	-2.45	-5.46	0.003302
PAN3	-2.45	-5.46	0.003103
SECISBP2L	-2.45	-5.46	0.001027
TAF7L	-2.45	-5.46	0.0034
UBR5	-2.45	-5.46	3.71E-05
DHX29	-2.45	-5.47	0.001523
EEA1	-2.45	-5.47	0.0008131
PCDHGA2	-2.45	-5.47	0.001908
PHLPP1	-2.45	-5.47	0.0001768
RAPGEF6	-2.45	-5.47	0.00081
TM4SF18	-2.45	-5.47	0.003404
DNAJC27	-2.45	-5.47	0.0004639
HSPA9	-2.45	-5.47	0.0007085
LRRC8D	-2.45	-5.47	0.002993
MARCKS	-2.45	-5.47	0.001038
METTL23	-2.45	-5.47	0.004946
OXCT1	-2.45	-5.47	7.31E-05
UGGT1	-2.45	-5.47	0.0008038
ADAMTSL3	-2.46	-5.48	0.0002009
CRIM1	-2.46	-5.48	0.0004101
PARD3	-2.46	-5.48	0.003376
AFTPH	-2.46	-5.49	0.001745
BTBD6	-2.46	-5.49	0.003113
DGKQ	-2.46	-5.49	0.0002433
GLG1	-2.46	-5.49	0.0024
GMNN	-2.46	-5.49	0.003057
QARS	-2.46	-5.49	0.0002567
STAG2	-2.46	-5.49	0.0003351
ZW10	-2.46	-5.49	0.003289
FAAP24	-2.46	-5.50	0.002162
FGFR3	-2.46	-5.50	0.0008418
HSPA12A	-2.46	-5.50	0.0005352
MID1	-2.46	-5.50	0.00198
NUF2	-2.46	-5.50	0.002511

OGFOD1	-2.46	-5.50	0.001257
SHROOM3	-2.46	-5.50	0.002199
TRMT1L	-2.46	-5.50	0.0002141
ADAMTS17	-2.46	-5.50	0.0006257
ALOX5	-2.46	-5.50	0.001061
EMILIN2	-2.46	-5.50	0.001916
ESCO2	-2.46	-5.50	0.0004875
FAM83B	-2.46	-5.50	0.002758
RIOX1	-2.46	-5.50	0.0013
SH3GL3	-2.46	-5.50	0.0004196
UAP1L1	-2.46	-5.50	0.0006071
ZNF644	-2.46	-5.50	0.004771
DCTN4	-2.46	-5.51	0.0001829
GNA13	-2.46	-5.51	0.0001948
GPR107	-2.46	-5.51	0.0002314
MBD5	-2.46	-5.51	0.001904
MVP	-2.46	-5.51	0.002651
PSEN1	-2.46	-5.51	0.004229
CNOT6	-2.46	-5.52	8.02E-06
SOCS6	-2.46	-5.52	0.002874
TGFB2	-2.46	-5.52	0.002048
TSPAN14	-2.46	-5.52	0.0003344
GNAL	-2.47	-5.52	0.004072
PRKDC	-2.47	-5.52	0.0006278
ACAT1	-2.47	-5.53	0.0008028
PISD	-2.47	-5.53	0.00189
PLTP	-2.47	-5.53	0.0001649
SEC23B	-2.47	-5.53	0.004299
TSC22D2	-2.47	-5.53	0.002874
DIDO1	-2.47	-5.54	0.00201
G2E3	-2.47	-5.54	0.0007604
HSPA8	-2.47	-5.54	0.004343
MARK1	-2.47	-5.54	3.44E-05
PCCA	-2.47	-5.54	0.004307
PSMC3	-2.47	-5.54	0.00179
AMER1	-2.47	-5.55	3.18E-05
FRMD6	-2.47	-5.55	0.005012
HSP90AA1	-2.47	-5.55	0.001895
IL17RD	-2.47	-5.55	2.26E-05
IVD	-2.47	-5.55	0.003692
LONRF2	-2.47	-5.55	0.001214
PANX3	-2.47	-5.55	4.78E-05
AHCTF1	-2.47	-5.56	1.48E-06
CCNF	-2.47	-5.56	3.54E-05
FAM120A	-2.47	-5.56	0.0001399
GNB2	-2.47	-5.56	0.001785
LYST	-2.47	-5.56	0.0006098
NPEPPS	-2.47	-5.56	0.004352
PARP1	-2.47	-5.56	0.0007104
PKM	-2.47	-5.56	0.0004919

SHCBP1	-2.47	-5.56	0.0002845
STXBP5L	-2.47	-5.56	0.0003761
AGPAT4	-2.48	-5.56	0.002709
CLCC1	-2.48	-5.56	0.00505
FAF1	-2.48	-5.56	0.004066
ABCC13	-2.48	-5.57	0.001887
CPD	-2.48	-5.57	0.0001835
DHX15	-2.48	-5.57	0.000238
DIAPH2	-2.48	-5.57	9.96E-06
HSP90AB1	-2.48	-5.57	0.0003633
IPMK	-2.48	-5.57	0.004695
RPGRIP1L	-2.48	-5.57	0.00412
SRPX	-2.48	-5.57	0.002454
SYNE2	-2.48	-5.57	0.000272
TBCK	-2.48	-5.57	0.0005538
TLN1	-2.48	-5.57	3.91E-05
MLYCD	-2.48	-5.58	0.004552
PI4K2B	-2.48	-5.58	0.001936
PSEN2	-2.48	-5.58	0.000484
RAPGEF1	-2.48	-5.58	0.0001803
UBR1	-2.48	-5.58	6.15E-05
ESRRG	-2.48	-5.58	0.004807
IL1R1	-2.48	-5.58	0.0003222
STAG1	-2.48	-5.58	0.00336
YPEL1	-2.48	-5.58	0.0008162
DHTKD1	-2.48	-5.59	0.001347
EIF2B5	-2.48	-5.59	0.001063
ELMSAN1	-2.48	-5.59	0.0004506
FAM69B	-2.48	-5.59	0.002078
FNIP2	-2.48	-5.59	0.0004944
KDM3A	-2.48	-5.59	0.002944
KIF13A	-2.48	-5.59	0.0008683
PTPRS	-2.48	-5.59	0.0005272
RBL2	-2.48	-5.59	0.0003948
ZNF407	-2.48	-5.59	0.0001043
ECPAS	-2.48	-5.60	0.0006762
IRX2	-2.48	-5.60	6.90E-06
LAMA5	-2.48	-5.60	0.0006683
NFS1	-2.48	-5.60	0.002135
SHC4	-2.48	-5.60	0.001303
HSPA4L	-2.49	-5.60	0.0004222
AP3B1	-2.49	-5.61	0.0009742
FAM129A	-2.49	-5.61	0.0003294
HINFP	-2.49	-5.61	0.001814
NOP14	-2.49	-5.61	0.001818
TENM2	-2.49	-5.61	8.08E-06
NUP160	-2.49	-5.62	1.16E-05
PLOD1	-2.49	-5.62	0.00115
SMPD4	-2.49	-5.62	0.001493
TPR	-2.49	-5.62	3.75E-05

ANAPC1	-2.49	-5.62	0.0008034
BCAR3	-2.49	-5.62	0.0003606
COG1	-2.49	-5.62	0.005142
GTSE1	-2.49	-5.62	0.001764
KDM4A	-2.49	-5.62	0.005096
KLF6	-2.49	-5.62	0.004447
NSF	-2.49	-5.62	0.001901
PPP1R14C	-2.49	-5.62	8.50E-06
TMEM128	-2.49	-5.62	0.005217
AKAP1	-2.49	-5.63	0.001774
CREBZF	-2.49	-5.63	0.0001488
INTS12	-2.49	-5.63	0.005157
KIF15	-2.49	-5.63	9.74E-07
KLHL29	-2.49	-5.63	0.0005144
STK4	-2.49	-5.63	0.003807
TLE1	-2.49	-5.63	1.08E-05
ZZZ3	-2.49	-5.63	0.0004741
NCALD	-2.49	-5.64	0.004487
TMEM164	-2.49	-5.64	0.004497
CEBPZ	-2.50	-5.64	0.003486
GGA1	-2.50	-5.64	0.00213
KBTBD8	-2.50	-5.64	0.0002779
MCM5	-2.50	-5.64	1.10E-05
C3AR1	-2.50	-5.65	2.33E-06
EFNB2	-2.50	-5.65	0.0004697
KCNMA1	-2.50	-5.65	0.003908
MYO1E	-2.50	-5.65	0.0001211
PDIA3	-2.50	-5.65	0.001719
PIGS	-2.50	-5.65	0.002372
RDH12	-2.50	-5.65	0.001717
SACS	-2.50	-5.65	1.60E-05
SLC4A1AP	-2.50	-5.65	0.002282
WIF1	-2.50	-5.65	6.91E-06
ABRAXAS2	-2.50	-5.66	0.004542
CCT4	-2.50	-5.66	0.001893
DIO3	-2.50	-5.66	0.0003423
MCM3AP	-2.50	-5.66	0.003432
MIPOL1	-2.50	-5.66	0.002854
PDS5A	-2.50	-5.66	0.0003186
PRDM2	-2.50	-5.66	0.0002797
CKAP4	-2.50	-5.66	0.000227
MB21D1	-2.50	-5.66	0.0002035
PGRMC2	-2.50	-5.66	0.0003718
RANBPL	-2.50	-5.66	5.27E-05
KIF1C	-2.50	-5.67	2.38E-05
SLC24A3	-2.50	-5.67	0.0007264
SLC43A2	-2.50	-5.67	0.0006817
DGKZ	-2.51	-5.68	0.002133
FEM1C	-2.51	-5.68	0.001016
IGFBP3	-2.51	-5.68	0.004816

MPP5	-2.51	-5.68	0.001573
ARMC8	-2.51	-5.68	0.000191
KPNA4	-2.51	-5.68	0.004151
PNPLA8	-2.51	-5.68	0.001666
TMEM87A	-2.51	-5.68	0.0004006
YARS	-2.51	-5.68	0.001686
CEP170P1	-2.51	-5.69	0.00135
DUSP8	-2.51	-5.69	0.0003264
LOC101752211	-2.51	-5.69	0.002632
RBBP5	-2.51	-5.69	0.003386
SCFD1	-2.51	-5.69	0.003116
TCP1	-2.51	-5.69	0.005381
CCDC66	-2.51	-5.70	0.003058
CEP63	-2.51	-5.70	0.001361
FXR1	-2.51	-5.70	0.003633
GATAD2A	-2.51	-5.70	0.001904
PLBD1	-2.51	-5.70	0.0006732
RIF1	-2.51	-5.70	0.0003479
SGK3	-2.51	-5.70	0.001968
TUBAL3	-2.51	-5.70	1.03E-06
ARVCF	-2.51	-5.71	5.97E-05
BHLHE40	-2.51	-5.71	0.003699
CUL5	-2.51	-5.71	0.00437
DHDDS	-2.51	-5.71	0.0001849
ACAT2	-2.51	-5.71	0.005042
BIRC5	-2.51	-5.71	0.0004203
CAT	-2.51	-5.71	0.005054
DENND2C	-2.51	-5.71	0.002168
EPB41	-2.51	-5.71	0.0001095
LZTR1	-2.51	-5.71	0.002888
NEFL	-2.51	-5.71	2.17E-06
PCNT	-2.51	-5.71	3.21E-05
PLOD3	-2.51	-5.71	0.0002417
SMARCA2	-2.51	-5.71	0.002251
ZBTB43	-2.51	-5.71	0.0002846
DEPDC1	-2.52	-5.73	1.13E-05
ID1	-2.52	-5.73	4.70E-05
SLC16A1	-2.52	-5.73	0.0009043
BDH1B	-2.52	-5.73	0.0006064
LAMB1	-2.52	-5.73	0.0006683
NCLN	-2.52	-5.73	0.0001242
P4HA1	-2.52	-5.73	0.001147
SLC25A22	-2.52	-5.73	4.77E-05
TMEM120B	-2.52	-5.73	0.0003017
USP5	-2.52	-5.73	0.002365
ZNF767	-2.52	-5.73	0.0008551
BNC2	-2.52	-5.74	0.001759
CKS2	-2.52	-5.74	0.0003936
HABP4	-2.52	-5.74	0.0009457
KCTD7	-2.52	-5.74	0.00129

MMS22L	-2.52	-5.74	0.0003173
MRPL37	-2.52	-5.74	0.004721
PEX6	-2.52	-5.74	5.53E-05
SLC25A38	-2.52	-5.74	0.001676
TBCCD1	-2.52	-5.74	0.004937
TNS1	-2.52	-5.74	0.00158
DHODH	-2.52	-5.75	0.0008267
EEF1G	-2.52	-5.75	0.000158
FUBP1	-2.52	-5.75	4.56E-05
IFRD1	-2.52	-5.75	7.84E-06
IFT81	-2.52	-5.75	0.005069
IPO5	-2.52	-5.75	1.82E-05
NCAPD3	-2.52	-5.75	0.001035
NLGN1	-2.52	-5.75	0.0004108
SMTN_C	-2.52	-5.75	5.20E-06
UBXN7	-2.52	-5.75	0.00153
GCC2	-2.53	-5.76	0.0001509
IPO7	-2.53	-5.76	0.0009805
NASP	-2.53	-5.76	0.002862
SLC35B2	-2.53	-5.76	0.0004808
TMEM67	-2.53	-5.76	0.004782
AKAP17A	-2.53	-5.77	0.003196
CNMD	-2.53	-5.77	5.95E-09
GPSM2	-2.53	-5.77	0.001662
HEPH	-2.53	-5.77	0.003143
INSC	-2.53	-5.77	3.30E-05
LETM1	-2.53	-5.77	0.0005746
MED15	-2.53	-5.77	0.004898
NKRF	-2.53	-5.77	6.76E-06
PHF20L1	-2.53	-5.77	0.005034
ZFAND4	-2.53	-5.77	0.003429
GLTPD2	-2.53	-5.77	0.002149
ICMT	-2.53	-5.77	0.003486
KATNAL1	-2.53	-5.77	0.004916
NAF1	-2.53	-5.77	0.002718
NPAS3	-2.53	-5.77	1.97E-05
WDR91	-2.53	-5.77	0.001442
ATF4	-2.53	-5.78	0.0009214
BCS1L	-2.53	-5.78	0.005156
FBXL4	-2.53	-5.78	0.0003704
GLE1	-2.53	-5.78	0.003421
GSPT2	-2.53	-5.78	0.003435
GTF2I	-2.53	-5.78	0.002611
MYO6	-2.53	-5.78	0.0007095
PTPRK	-2.53	-5.78	0.0002985
RFWD2	-2.53	-5.78	0.00325
SMG8	-2.53	-5.78	0.0004015
SACM1L	-2.53	-5.79	0.0001003
SCAF4	-2.53	-5.79	0.0004854
SLC45A4	-2.53	-5.79	0.002609

TMCO7	-2.53	-5.79	0.0001518
KNOP1	-2.54	-5.80	0.005206
NADSYN1	-2.54	-5.80	0.002877
NUP210	-2.54	-5.80	0.001914
PQLC2	-2.54	-5.80	0.003459
PTPRF	-2.54	-5.80	0.0004301
SF3B3	-2.54	-5.80	0.0002712
TBP	-2.54	-5.80	0.002405
TMEM131L	-2.54	-5.80	0.003934
TMEM68	-2.54	-5.80	0.002142
WASHC1	-2.54	-5.80	0.003867
ZNF518A	-2.54	-5.80	0.001171
DUSP1	-2.54	-5.80	0.0005928
EIF4G3	-2.54	-5.80	8.03E-05
G0S2	-2.54	-5.80	0.001049
GPI	-2.54	-5.80	0.0002248
GUSB	-2.54	-5.80	0.000254
RUNX1T1	-2.54	-5.80	0.000147
TERF2	-2.54	-5.80	0.004955
VPS13D	-2.54	-5.80	0.002296
ALDH5A1	-2.54	-5.81	0.001878
KIF23	-2.54	-5.81	0.0007935
LFNG	-2.54	-5.81	1.14E-06
RRM1	-2.54	-5.81	0.000172
SEPT6	-2.54	-5.81	0.002524
TM7SF2	-2.54	-5.81	0.0003021
ARMC1	-2.54	-5.82	0.001674
COL4A3BP	-2.54	-5.82	0.00163
KIAA1429	-2.54	-5.82	0.0002702
LIMS1	-2.54	-5.82	0.001444
METTL4	-2.54	-5.82	3.79E-05
MYO5A	-2.54	-5.82	0.000293
PPP1R12A	-2.54	-5.82	0.0003593
PTBP1	-2.54	-5.82	0.0003208
TTC21B	-2.54	-5.82	0.000133
WDR20	-2.54	-5.82	0.0009614
AGTR2	-2.54	-5.82	1.97E-05
ARL13B	-2.54	-5.82	0.003305
NPRL3	-2.54	-5.82	0.004087
RAF1	-2.54	-5.82	0.00484
RNASEL	-2.54	-5.82	0.003046
UNC5B	-2.54	-5.82	0.0004353
CPEB2	-2.54	-5.83	0.004446
IPO11	-2.54	-5.83	0.0005375
KATNBL1	-2.54	-5.83	0.0002246
MSI2	-2.54	-5.83	0.001034
USP7	-2.54	-5.83	7.56E-06
VSTM4	-2.54	-5.83	0.003731
CLK2	-2.55	-5.84	0.0006909
COPG1	-2.55	-5.84	0.0003038

FAM20B	-2.55	-5.84	0.002554
RFC3	-2.55	-5.84	0.001494
TNFRSF1B	-2.55	-5.84	9.13E-07
ADPRHL2	-2.55	-5.85	0.002707
ATP5F1C	-2.55	-5.85	7.13E-05
HSPD1	-2.55	-5.85	0.0006906
LTO1	-2.55	-5.85	0.0002042
ACBD3	-2.55	-5.85	0.001492
ARSB	-2.55	-5.85	0.001076
FNIP1	-2.55	-5.85	0.002044
IST1	-2.55	-5.85	0.001869
NADK	-2.55	-5.85	8.29E-05
POLE3	-2.55	-5.85	0.003269
SLC35F5	-2.55	-5.85	0.0001451
TIAM1	-2.55	-5.85	0.0001858
TLR2A	-2.55	-5.85	5.13E-06
YEATS2	-2.55	-5.85	0.0003315
AKAP12	-2.55	-5.86	1.69E-06
ARHGEF3	-2.55	-5.86	0.0006639
ASCC3	-2.55	-5.86	0.0002124
ENC1	-2.55	-5.86	0.0009227
MTDH	-2.55	-5.86	0.0001995
PDE4D	-2.55	-5.86	2.49E-05
SMARCA1	-2.55	-5.86	0.001096
ARFGEF1	-2.55	-5.87	1.95E-05
DCX	-2.55	-5.87	7.90E-05
DICER1	-2.55	-5.87	0.0001034
NXT2	-2.55	-5.87	0.002898
RBM12B	-2.55	-5.87	0.004165
RRM2	-2.55	-5.87	6.61E-05
UTP18	-2.55	-5.87	0.005161
ASNS	-2.56	-5.88	0.001769
ELL2	-2.56	-5.88	0.002586
FBXO30	-2.56	-5.88	4.89E-05
GCLC	-2.56	-5.88	0.0003518
KAT14	-2.56	-5.88	0.005344
ANKRD50	-2.56	-5.89	0.0005384
C1H12orf29	-2.56	-5.89	0.004943
KMT2E	-2.56	-5.89	5.13E-06
OSBPL2	-2.56	-5.89	0.004395
PIK3CB	-2.56	-5.89	0.0009814
RRBP1	-2.56	-5.89	2.00E-05
TADA1	-2.56	-5.89	0.001714
UFL1	-2.56	-5.89	0.001064
ANO3	-2.56	-5.90	3.48E-06
ESF1	-2.56	-5.90	0.001467
F2R	-2.56	-5.90	0.0001117
KLHL7	-2.56	-5.90	7.79E-05
RNF214	-2.56	-5.90	0.004965
RPL27	-2.56	-5.90	0.00124

SASS6	-2.56	-5.90	0.001287
SCYL2	-2.56	-5.90	0.0006141
SLC33A1	-2.56	-5.90	0.003605
SPTAN1	-2.56	-5.90	4.25E-07
ARIH2	-2.56	-5.90	0.003093
C24H11orf57	-2.56	-5.90	0.002598
CKAP2	-2.56	-5.90	0.0001416
COL5A1	-2.56	-5.90	0.0002493
LRRC45	-2.56	-5.90	0.00434
N4BP2L2	-2.56	-5.90	0.0005232
BUB1	-2.56	-5.91	0.0001832
IMPAD1	-2.56	-5.91	0.002025
POC1B	-2.56	-5.91	0.002217
PRSS35	-2.56	-5.91	0.002845
EDEM2	-2.57	-5.92	0.001213
ERC1	-2.57	-5.92	1.37E-06
MINPP1	-2.57	-5.92	0.0001631
ORC1	-2.57	-5.92	1.83E-05
COPB1	-2.57	-5.93	0.001273
HMGB3	-2.57	-5.93	0.0006729
MRM3	-2.57	-5.93	0.002113
ZNF622	-2.57	-5.93	0.001767
DIAPH3	-2.57	-5.93	0.0006984
PACSIN3	-2.57	-5.93	0.002566
PGAM5	-2.57	-5.93	0.004088
POLR3B	-2.57	-5.93	1.04E-06
TIAM2	-2.57	-5.93	9.51E-07
ACTC1	-2.57	-5.94	8.59E-08
AP2A2	-2.57	-5.94	0.0003065
CC2D2A	-2.57	-5.94	0.002909
HK1	-2.57	-5.94	0.0004719
MAP4	-2.57	-5.94	4.14E-06
NDC1	-2.57	-5.94	0.0003177
PQLC3	-2.57	-5.94	0.0003558
SLC12A7	-2.57	-5.94	2.72E-05
VAV3	-2.57	-5.94	0.002277
GPT	-2.57	-5.95	6.47E-05
LOXL3	-2.57	-5.95	1.25E-05
MET	-2.57	-5.95	0.0009442
PPP1R9B	-2.57	-5.95	0.005351
TLN2	-2.57	-5.95	8.43E-05
ZCCHC7	-2.57	-5.95	0.0009112
ACTA1	-2.57	-5.95	0.0001017
CCDC14	-2.57	-5.95	0.0006971
GTF3C4	-2.57	-5.95	3.81E-05
LRBA	-2.57	-5.95	0.002853
NEDD9	-2.57	-5.95	3.35E-08
ZBTB10	-2.57	-5.95	0.0007656
ARPC5L	-2.58	-5.96	0.002575
CLTCL1	-2.58	-5.96	0.0004751

ESPL1	-2.58	-5.96	0.0006872
FBXO8	-2.58	-5.96	0.001297
HIGD1A	-2.58	-5.96	0.000452
LANCL1	-2.58	-5.96	0.003244
NAGA	-2.58	-5.96	0.001598
PEX12	-2.58	-5.96	0.004094
SASH1	-2.58	-5.96	1.83E-06
SEMA3C	-2.58	-5.96	0.0001758
HERPUD1	-2.58	-5.97	3.47E-06
PIM1	-2.58	-5.97	4.06E-05
RRN3	-2.58	-5.97	4.12E-06
SCAF11	-2.58	-5.97	0.003102
TM9SF4	-2.58	-5.97	0.001356
UBASH3B	-2.58	-5.97	0.0001328
AMD1	-2.58	-5.98	0.0003649
CCNDBP1	-2.58	-5.98	0.004242
HELQ	-2.58	-5.98	0.001835
MATN3	-2.58	-5.98	0.000289
SNX13	-2.58	-5.98	0.005334
TGDS	-2.58	-5.98	0.004699
CAST	-2.58	-5.98	0.0001373
INTS2	-2.58	-5.98	0.001704
LARP4	-2.58	-5.98	0.0005279
NCBP3	-2.58	-5.98	0.002591
PPP3CB	-2.58	-5.98	0.0003704
RAB9B	-2.58	-5.98	0.003108
RAE1	-2.58	-5.98	0.0002437
ARL4C	-2.58	-5.99	0.0001506
ASPSCR1	-2.58	-5.99	0.004055
CDKN2AIP	-2.58	-5.99	0.003358
CENPO	-2.58	-5.99	0.001755
LAPTM5	-2.58	-5.99	2.85E-06
PRIM2	-2.58	-5.99	0.0006638
SRP68	-2.58	-5.99	0.004521
SYNCRIP	-2.58	-5.99	0.00201
TMEM8C	-2.58	-5.99	3.44E-07
ZC3H14	-2.58	-5.99	0.002439
ABCD3	-2.58	-6.00	6.64E-06
ATP13A1	-2.58	-6.00	0.0002652
CASD1	-2.58	-6.00	0.001203
COL10A1	-2.58	-6.00	0.0001236
LAS1L	-2.58	-6.00	0.0003787
LRRFIP2	-2.58	-6.00	0.004573
NUDT19	-2.58	-6.00	3.29E-05
TSC1	-2.58	-6.00	0.003096
WDR36	-2.58	-6.00	0.0002632
COL1A1	-2.59	-6.01	0.0004509
FZD1	-2.59	-6.01	0.003485
GNA12	-2.59	-6.01	0.001508
LRRK2	-2.59	-6.01	0.0005556

SREK1	-2.59	-6.01	0.0008067
UBA5	-2.59	-6.01	0.0001593
CDKN3	-2.59	-6.01	0.001653
MIEF2	-2.59	-6.01	0.0008161
PRDM15	-2.59	-6.01	0.002565
SYNM	-2.59	-6.01	0.0001455
EPB41L2	-2.59	-6.02	0.001405
LRRC3B	-2.59	-6.02	0.001959
MARCH6	-2.59	-6.02	0.000568
ZC3H15	-2.59	-6.02	0.005233
AMBRA1	-2.59	-6.03	0.00171
MYOCD	-2.59	-6.03	2.11E-05
NLRP1L	-2.59	-6.03	2.38E-05
PANK4	-2.59	-6.03	0.003457
PLEKHF2	-2.59	-6.03	0.002116
RHOU	-2.59	-6.03	2.01E-05
BRAF	-2.59	-6.04	0.0006644
GFPT1	-2.59	-6.04	0.0003914
LRPPRC	-2.59	-6.04	1.27E-05
MAP2	-2.59	-6.04	0.0004813
MPP6	-2.59	-6.04	0.001304
NCOR1	-2.59	-6.04	9.16E-05
NOMO3	-2.59	-6.04	0.003039
OTUD3	-2.59	-6.04	0.001595
AUH	-2.60	-6.04	0.0009489
SLC20A2	-2.60	-6.04	0.001108
TLK1L	-2.60	-6.04	3.43E-06
CAPRIN2	-2.60	-6.05	0.0006926
CDH2	-2.60	-6.05	6.82E-05
LUC7L	-2.60	-6.05	0.0003887
PINX1	-2.60	-6.05	0.004176
PNPLA7	-2.60	-6.05	0.001085
UHRF1	-2.60	-6.05	6.81E-06
CSTF2	-2.60	-6.06	0.0006474
NR3C2	-2.60	-6.06	0.0001018
SIK1	-2.60	-6.06	3.59E-05
WDR4	-2.60	-6.06	1.78E-05
ARV1	-2.60	-6.07	0.0007309
CLDN12	-2.60	-6.07	0.004591
DST	-2.60	-6.07	0.0001702
KCNK2	-2.60	-6.07	1.44E-06
KSR1	-2.60	-6.07	0.002155
PRKX	-2.60	-6.07	3.69E-06
RASGEF1A	-2.60	-6.07	8.02E-07
SGSM2	-2.60	-6.07	0.003895
THSD4	-2.60	-6.07	5.23E-06
ATP2B1	-2.60	-6.07	0.000498
BAIAP2L1	-2.60	-6.07	0.001773
PANK3	-2.60	-6.07	0.0007524
PDCD7	-2.60	-6.07	0.001032

PPP4R1L	-2.60	-6.07	2.02E-05
PRKAA1	-2.60	-6.07	0.0005071
TK1	-2.60	-6.07	0.0003365
LMCD1	-2.60	-6.08	0.0001264
LRRFIP1	-2.60	-6.08	0.003077
MTHFSD	-2.60	-6.08	0.003265
PCMTD2	-2.60	-6.08	0.00134
RLF	-2.60	-6.08	0.001304
TRIM13	-2.60	-6.08	0.0001649
CDADC1	-2.61	-6.09	0.002768
CTR9	-2.61	-6.09	5.85E-05
DSE	-2.61	-6.09	0.001168
MTHFD1	-2.61	-6.09	3.49E-05
AQP1	-2.61	-6.10	0.001048
MIS18BP1	-2.61	-6.10	6.30E-05
NUP214	-2.61	-6.10	0.001358
SLC15A4	-2.61	-6.10	0.0003928
SLK	-2.61	-6.10	0.0001574
USP9X	-2.61	-6.10	0.0002863
ATRN	-2.61	-6.10	0.001273
EEFSEC	-2.61	-6.10	0.001297
EME1	-2.61	-6.10	0.002401
RNMT	-2.61	-6.10	0.0007668
SLC1A6	-2.61	-6.10	0.0009972
TMEM2	-2.61	-6.10	0.0002964
C10ORF12	-2.61	-6.11	1.69E-05
HOOK3	-2.61	-6.11	6.56E-05
LRRC8A	-2.61	-6.11	2.19E-05
QSER1	-2.61	-6.11	0.0004571
ZFHX3	-2.61	-6.11	0.0001477
BRF1	-2.61	-6.12	0.0003704
CAND1	-2.61	-6.12	0.00354
SCAPER	-2.61	-6.12	0.005011
TOLLIP	-2.61	-6.12	0.002244
GSTT1	-2.62	-6.13	0.0002262
MDM1	-2.62	-6.13	0.003726
NUP205	-2.62	-6.13	0.0001099
PLPP3	-2.62	-6.13	0.0009719
REPS1	-2.62	-6.13	5.88E-05
WDR18	-2.62	-6.13	0.002818
MCPH1	-2.62	-6.14	0.0002584
PDHX	-2.62	-6.14	0.0001309
PDP2	-2.62	-6.14	2.84E-06
POT1	-2.62	-6.14	0.002824
SCYL3	-2.62	-6.14	0.003465
SDHA	-2.62	-6.14	0.002309
VRK1	-2.62	-6.14	0.0004733
WAC	-2.62	-6.14	0.001329
ETFBKMT	-2.62	-6.14	0.0006434
FAM114A2	-2.62	-6.14	0.0007215

HSP90B1	-2.62	-6.14	0.001293
SLIT2	-2.62	-6.14	0.0001296
GPR34	-2.62	-6.15	1.60E-07
KCNK5	-2.62	-6.15	3.51E-07
LARS	-2.62	-6.15	0.0003006
MCF2	-2.62	-6.15	0.0005052
MTMR8	-2.62	-6.15	0.000639
NSUN2	-2.62	-6.15	0.0004505
SLC16A3	-2.62	-6.15	0.0001577
SMURF2	-2.62	-6.15	3.44E-05
ZNF292	-2.62	-6.15	0.0003473
CHTF18	-2.62	-6.16	0.0007727
GNAI1	-2.62	-6.16	0.001515
MVB12B	-2.62	-6.16	0.001628
NSUN4	-2.62	-6.16	0.002894
RHNO1	-2.62	-6.16	0.0006976
SPTLC2	-2.62	-6.16	0.0002291
FOXO3	-2.62	-6.17	0.000305
GCN1	-2.62	-6.17	4.43E-05
MIA3	-2.62	-6.17	1.51E-05
WDR47	-2.62	-6.17	0.001388
USP34	-2.63	-6.17	0.0003209
ADCK2	-2.63	-6.18	0.00198
COMT	-2.63	-6.18	5.61E-05
NTHL1	-2.63	-6.18	0.005227
PDCD4	-2.63	-6.18	0.001945
PLEKHO1	-2.63	-6.18	0.001358
PKP2	-2.63	-6.19	0.001305
TMEM260	-2.63	-6.19	0.001071
PELO	-2.63	-6.20	0.003902
TERF1	-2.63	-6.20	0.0007336
TSPAN6	-2.63	-6.20	0.001222
XPO4	-2.63	-6.20	0.001159
AMDHD2	-2.63	-6.21	0.0005359
JUP	-2.63	-6.21	0.0002164
MDM2	-2.63	-6.21	1.32E-05
PREX1	-2.63	-6.21	0.003089
ABI2	-2.64	-6.21	3.49E-05
F2RL1	-2.64	-6.21	0.0001378
ITPK1	-2.64	-6.21	6.44E-05
KTN1	-2.64	-6.21	0.0001192
RRM2B	-2.64	-6.21	0.005304
TPRA1	-2.64	-6.21	0.00133
ARHGAP21	-2.64	-6.22	0.0004647
CPOX	-2.64	-6.22	0.00206
NEO1	-2.64	-6.22	0.001356
NGLY1	-2.64	-6.22	0.0002108
PIK3CA	-2.64	-6.22	0.004427
SEC14L1	-2.64	-6.22	0.0005668
CDKN1B	-2.64	-6.23	0.002629

KIF4A	-2.64	-6.23	2.29E-07
SEC23IP	-2.64	-6.23	0.005327
SESN3	-2.64	-6.23	4.73E-05
ABCC4	-2.64	-6.24	0.0002309
ARHGAP28	-2.64	-6.24	0.001877
CHPF2	-2.64	-6.24	0.003734
CRY1	-2.64	-6.24	0.0008012
DIEXF	-2.64	-6.24	0.0002931
DOP1B	-2.64	-6.24	0.0001539
C5H14ORF169	-2.64	-6.24	0.0001822
C8H1orf27	-2.64	-6.24	0.0002569
CDK11A	-2.64	-6.24	0.002015
GTF2A1	-2.64	-6.24	0.001235
MCCC2L	-2.64	-6.24	0.000428
NAPG	-2.64	-6.24	2.08E-06
NCAPD2	-2.64	-6.24	1.43E-06
PDE3B	-2.64	-6.24	7.43E-05
RAD51C	-2.64	-6.24	0.00226
WDR33	-2.64	-6.24	0.0001027
ACSBG2	-2.64	-6.25	0.0004161
CCNJ	-2.64	-6.25	4.07E-05
ELAC2	-2.64	-6.25	2.13E-05
MPHOSPH10	-2.64	-6.25	0.0002971
MRPL22	-2.64	-6.25	0.002176
NAA16	-2.64	-6.25	6.53E-05
OSBPL5	-2.64	-6.25	0.0009122
PPIL2	-2.64	-6.25	0.003552
ALG6	-2.65	-6.26	0.0003433
GIGYF2	-2.65	-6.26	0.00191
HNRNPAB	-2.65	-6.26	0.002833
NLN	-2.65	-6.26	0.002625
PARD6G	-2.65	-6.26	0.004493
PSME4	-2.65	-6.26	7.87E-05
ELP2	-2.65	-6.27	0.0002057
GRB10	-2.65	-6.27	0.0003359
IQGAP3	-2.65	-6.27	3.44E-05
NEK2	-2.65	-6.27	9.02E-07
STON1	-2.65	-6.27	0.001517
UBE2G1	-2.65	-6.27	0.0043
CEP68	-2.65	-6.28	4.17E-05
CEPT1	-2.65	-6.28	0.003417
DYNC1LI2	-2.65	-6.28	0.0009856
HIC2	-2.65	-6.28	0.00146
NUP35	-2.65	-6.28	0.001258
SEC24C	-2.65	-6.28	0.002126
SLC35E1	-2.65	-6.28	0.004073
TRAF6	-2.65	-6.28	6.71E-05
UGGT2	-2.65	-6.28	0.000433
WASHC2C	-2.65	-6.28	0.002396
DDX49	-2.65	-6.28	0.0001412

MICALL1	-2.65	-6.28	0.004166
NUP58	-2.65	-6.28	0.0004036
SF3A1	-2.65	-6.28	0.000664
SNRNP48	-2.65	-6.28	0.001637
ASAP1	-2.65	-6.29	0.004232
CCDC117	-2.65	-6.29	0.003067
SKA3	-2.65	-6.29	9.67E-05
DOCK10	-2.66	-6.30	0.0006196
INTU	-2.66	-6.30	2.14E-05
NUAK1	-2.66	-6.30	6.74E-06
TPX2	-2.66	-6.30	1.53E-05
U2SURP	-2.66	-6.30	0.0001236
ZNF521	-2.66	-6.30	0.001437
FAM107B	-2.66	-6.31	0.000559
FZD6	-2.66	-6.31	0.003744
MICU2	-2.66	-6.31	0.0002825
PPP2R5E	-2.66	-6.31	0.001761
SUGP2	-2.66	-6.31	0.0001545
CLUH	-2.66	-6.32	1.84E-05
GMPS	-2.66	-6.32	0.0004524
IL1RAP	-2.66	-6.32	0.002657
LOC419074	-2.66	-6.32	0.0002187
PRIM1	-2.66	-6.32	0.0001731
RAD1	-2.66	-6.32	0.00222
TBC1D14	-2.66	-6.32	0.0006459
WDR77	-2.66	-6.32	0.0001578
IFNAR1	-2.66	-6.32	0.0005613
SERPINH1	-2.66	-6.33	0.00056
SPG11	-2.66	-6.33	0.0003442
SRRM1	-2.66	-6.33	0.000139
TRMT44	-2.66	-6.33	0.002634
WDR82	-2.66	-6.33	0.002975
WFS1	-2.66	-6.33	0.0005509
ARHGAP42	-2.66	-6.34	0.0005539
DDX3X	-2.66	-6.34	0.0009723
WDR3	-2.66	-6.34	2.25E-05
XRCC5	-2.66	-6.34	0.0001917
ACACA	-2.67	-6.35	1.06E-05
KIAA1524	-2.67	-6.35	1.70E-05
KIAA2026	-2.67	-6.35	0.0006315
SLC6A15	-2.67	-6.35	3.87E-05
ZUFSP	-2.67	-6.35	0.001057
BLM	-2.67	-6.36	0.0002013
COPA	-2.67	-6.36	5.62E-05
CUL3	-2.67	-6.36	0.0002569
ORMDL3	-2.67	-6.36	0.00101
RARB	-2.67	-6.36	0.0002842
SCAI	-2.67	-6.36	0.0001915
LTBP1	-2.67	-6.37	0.0003348
SESN1	-2.67	-6.37	0.000142

SGMS2	-2.67	-6.37	0.0005896
SUCLG2	-2.67	-6.37	0.0009614
TMEM170A	-2.67	-6.37	0.0002283
WASHC5	-2.67	-6.37	0.0003681
ATM	-2.67	-6.37	0.0002052
GOLGA5	-2.67	-6.37	0.0001376
LIN7C	-2.67	-6.37	0.0001368
RINT1	-2.67	-6.37	0.0004107
SPAST	-2.67	-6.37	0.0007026
TOP2B	-2.67	-6.37	0.0002227
USP40	-2.67	-6.37	0.00123
YLPM1	-2.67	-6.37	1.01E-05
ACADS	-2.67	-6.38	0.001415
DNAJC21	-2.67	-6.38	0.002619
DROSHA	-2.67	-6.38	0.001489
HEATR1	-2.67	-6.38	3.26E-07
HEXDC	-2.67	-6.38	0.0002975
MSH6	-2.67	-6.38	5.68E-05
NBR1	-2.67	-6.38	0.001215
TCEANC	-2.67	-6.38	0.004754
TROVE2	-2.67	-6.38	0.002801
CDK8	-2.68	-6.39	0.003139
COPG2	-2.68	-6.39	0.001063
CXADR	-2.68	-6.39	2.12E-06
KIAA1456	-2.68	-6.39	0.001141
MCCC1	-2.68	-6.39	0.0002418
PIGQ	-2.68	-6.39	0.0002562
PTGFR	-2.68	-6.39	6.34E-05
SELENOO	-2.68	-6.39	0.002537
ACAP3	-2.68	-6.40	0.001821
EZR	-2.68	-6.40	0.0001029
NUP188	-2.68	-6.40	0.0001031
AHNAK2	-2.68	-6.41	5.51E-09
CEP57	-2.68	-6.41	0.000182
LBH	-2.68	-6.41	0.003825
MAP1LC3C	-2.68	-6.41	5.67E-06
MAP2K4	-2.68	-6.41	0.0003757
METRNL	-2.68	-6.41	0.003938
MSMO1	-2.68	-6.41	0.0002459
NDUFS1	-2.68	-6.41	0.0002232
SMYD4	-2.68	-6.41	0.004411
SNAPC1	-2.68	-6.41	0.004236
UGCG	-2.68	-6.41	3.39E-06
ASTE1	-2.68	-6.41	1.13E-05
ERCC4	-2.68	-6.41	4.29E-06
GNAI3	-2.68	-6.41	0.002913
GTF3C5	-2.68	-6.41	0.0002297
PXDN	-2.68	-6.41	4.08E-07
SEMA3D	-2.68	-6.41	0.0001254
ABCB7	-2.68	-6.42	6.90E-05

ACSL4	-2.68	-6.42	0.0001224
ITGA1	-2.68	-6.42	0.004325
PFKFB4L	-2.68	-6.42	0.0008178
C1H3ORF38	-2.69	-6.43	0.002366
CHPF	-2.69	-6.43	0.0003174
LRRC28	-2.69	-6.43	0.003526
MAD2L1	-2.69	-6.43	0.0003826
NHLRC1	-2.69	-6.43	0.0005329
TRMT6	-2.69	-6.43	0.0003804
ZDHHC15	-2.69	-6.43	0.0007142
ZNF488	-2.69	-6.43	0.0009914
FUT8	-2.69	-6.44	0.0006281
SPRED2	-2.69	-6.44	0.0002207
TTC14	-2.69	-6.44	0.0002116
AP1AR	-2.69	-6.45	0.0004138
CMTR2	-2.69	-6.45	0.002489
R3HCC1	-2.69	-6.45	0.0004671
ANKRD13C	-2.69	-6.46	0.002461
FBLN2	-2.69	-6.46	7.81E-06
GNAS	-2.69	-6.46	0.001128
GRN	-2.69	-6.46	0.0001481
PHF14	-2.69	-6.46	0.003579
TMEM268	-2.69	-6.46	0.00112
AGRN	-2.69	-6.46	1.59E-06
BCL7A	-2.69	-6.46	4.05E-05
GTDC1	-2.69	-6.46	0.0003876
GTF3C1	-2.69	-6.46	0.0001428
WDR1	-2.69	-6.46	0.001329
YPEL2	-2.69	-6.46	0.000285
CCSER1	-2.69	-6.47	7.97E-06
LRP8	-2.69	-6.47	1.66E-06
RHOBTB1	-2.69	-6.47	4.62E-07
USP3	-2.69	-6.47	0.0003318
CEP97	-2.70	-6.48	2.21E-05
RGMA	-2.70	-6.48	0.0001597
FBXW11	-2.70	-6.49	0.002276
FMN1	-2.70	-6.49	0.0008123
STX2	-2.70	-6.49	0.002978
CEP85L	-2.70	-6.50	0.001087
CLCN5	-2.70	-6.50	0.0005263
APOOL	-2.70	-6.51	0.00349
CTTNBP2NL	-2.70	-6.51	0.0009693
DUSP10	-2.70	-6.51	0.000843
LOC416055	-2.70	-6.51	0.0003561
TNFAIP8	-2.70	-6.51	0.001659
EXOSC3	-2.70	-6.52	0.0001199
PIK3C2A	-2.70	-6.52	7.20E-07
PWWP2B	-2.70	-6.52	0.0005477
RPP38	-2.70	-6.52	0.001377
PAXBP1	-2.71	-6.52	0.0005127

TGS1	-2.71	-6.52	6.44E-05
TYW3	-2.71	-6.52	0.0002934
VEZT	-2.71	-6.52	9.89E-05
CCND3	-2.71	-6.53	6.26E-05
FAR1	-2.71	-6.53	0.0005453
ICE2	-2.71	-6.53	0.0002652
MYBL1	-2.71	-6.53	5.78E-05
SDE2	-2.71	-6.53	9.04E-05
SLC9A3R2	-2.71	-6.53	0.0009165
USP38	-2.71	-6.53	0.003555
EDC3	-2.71	-6.54	8.18E-05
PTCD3	-2.71	-6.54	0.0004897
SMARCAD1	-2.71	-6.54	0.000189
EXO1	-2.71	-6.55	6.05E-07
TPRN	-2.71	-6.55	0.0002391
TUBGCP6	-2.71	-6.55	0.0001154
ERCC5	-2.71	-6.56	0.0005079
FITM2	-2.71	-6.56	0.001458
GRK4	-2.71	-6.56	0.0001451
PTPRJ	-2.71	-6.56	3.87E-06
RAB11FIP4	-2.71	-6.56	0.0002156
TRAPPC12	-2.71	-6.56	0.001259
TTF1	-2.71	-6.56	0.000127
TXNDC11	-2.71	-6.56	0.002108
ACKR3	-2.72	-6.57	0.000224
AEBP2	-2.72	-6.57	0.002414
AFAP1	-2.72	-6.57	0.0001337
ANAPC5	-2.72	-6.57	1.19E-05
EIPR1	-2.72	-6.57	0.0001532
MOB1B	-2.72	-6.57	0.0002509
MTIF2	-2.72	-6.57	0.002444
PRPF6	-2.72	-6.57	0.0002707
SMC4	-2.72	-6.57	2.68E-05
URB1	-2.72	-6.57	6.66E-07
CDC27	-2.72	-6.57	1.80E-05
HSPA4	-2.72	-6.57	5.33E-05
LDAH	-2.72	-6.57	0.0001687
NGFR	-2.72	-6.57	1.70E-08
PPP2R3A	-2.72	-6.57	0.0006702
TAMM41	-2.72	-6.57	0.0001824
ADRM1	-2.72	-6.58	0.002596
CLOCK	-2.72	-6.58	0.0005412
DPH3P1	-2.72	-6.58	0.0001983
EXOC8	-2.72	-6.58	0.001885
MMAA	-2.72	-6.58	0.001477
NSMCE4A	-2.72	-6.58	0.0007094
PMS2	-2.72	-6.58	0.001928
SLC35A2	-2.72	-6.58	0.0001843
TP53BP1	-2.72	-6.58	8.66E-05
AMACR	-2.72	-6.59	0.002742

HAUS3	-2.72	-6.59	8.57E-07
MYH9	-2.72	-6.59	5.55E-06
NOTUM	-2.72	-6.59	0.0001025
RRP1B	-2.72	-6.59	0.0006185
WDR7	-2.72	-6.59	0.0001448
CCDC51	-2.72	-6.60	0.003878
CDYL2	-2.72	-6.60	0.00228
RIN3	-2.72	-6.60	0.0003553
TRIP11	-2.72	-6.60	3.99E-06
ANAPC10	-2.72	-6.61	0.002003
DDB1	-2.72	-6.61	3.50E-06
SMARCD3	-2.72	-6.61	8.71E-06
B3GALT6	-2.73	-6.62	0.002222
FIGNL1	-2.73	-6.62	0.0007656
CLASP1	-2.73	-6.63	0.0003457
FBXO34	-2.73	-6.63	0.001745
P3H4	-2.73	-6.63	4.98E-05
RAB9A	-2.73	-6.63	0.000831
STIM2	-2.73	-6.63	0.0003678
USP28	-2.73	-6.63	8.17E-05
MYH15	-2.73	-6.64	6.86E-08
RFT1	-2.73	-6.64	4.31E-05
LTN1	-2.73	-6.64	0.0006812
NCAPG	-2.73	-6.64	2.29E-06
PLEKHG4	-2.73	-6.64	1.41E-05
POC1A	-2.73	-6.64	0.0004274
RPP40	-2.73	-6.64	0.001039
TBC1D8	-2.73	-6.64	4.76E-06
INPP5B	-2.73	-6.65	0.002385
MIR92A1	-2.73	-6.65	1.95E-08
ORC2	-2.73	-6.65	5.81E-06
PRICKLE2	-2.73	-6.65	0.0007976
ZNF451	-2.73	-6.65	0.003528
DIP2A	-2.74	-6.66	0.001141
MYO10	-2.74	-6.66	1.30E-06
NIN	-2.74	-6.66	3.73E-06
ALDH18A1	-2.74	-6.67	0.0001778
ANKRD28	-2.74	-6.67	0.004311
ARHGAP12	-2.74	-6.67	4.81E-06
DENND4C	-2.74	-6.67	3.88E-05
MIOS	-2.74	-6.67	0.001741
OSBPL11	-2.74	-6.67	0.001598
PREP	-2.74	-6.67	4.77E-05
ZNF217	-2.74	-6.67	0.0001919
C11H16orf70	-2.74	-6.68	0.0004324
FLCN	-2.74	-6.68	0.004255
GGNBP2	-2.74	-6.68	0.0001122
LPCAT1	-2.74	-6.68	0.0007662
PIGG	-2.74	-6.68	0.0007912
SF3B1	-2.74	-6.68	0.0002461

SLC39A8	-2.74	-6.68	9.72E-05
PBX3	-2.74	-6.69	0.001397
PUS10	-2.74	-6.69	0.0009918
SLC9A7	-2.74	-6.69	5.12E-05
YIPF6	-2.74	-6.69	0.0003807
DDX42	-2.74	-6.70	4.37E-05
MADD	-2.74	-6.70	0.002963
RAD21	-2.74	-6.70	0.001902
RAD54B	-2.74	-6.70	0.0001654
TBC1D8B	-2.74	-6.70	0.0007652
TRIP13	-2.74	-6.70	0.0001003
CCDC15	-2.75	-6.71	0.0003117
FPGT	-2.75	-6.71	0.005175
FTSJ3	-2.75	-6.71	8.51E-06
LIPT1	-2.75	-6.71	0.00258
SETD6	-2.75	-6.71	0.0003591
SPDL1	-2.75	-6.71	0.0001847
TBCD	-2.75	-6.71	8.69E-05
PDCD2L	-2.75	-6.71	0.0002669
PHAX	-2.75	-6.71	0.001852
SMC2	-2.75	-6.71	1.88E-06
CPT2	-2.75	-6.72	0.005134
SRFBP1	-2.75	-6.72	0.002969
CNEP1R1	-2.75	-6.73	0.00193
CNOT2	-2.75	-6.73	0.0006898
RANGAP1	-2.75	-6.73	6.10E-05
ZNF518B	-2.75	-6.73	8.92E-07
CENPT	-2.75	-6.74	3.30E-05
DLGAP5	-2.75	-6.74	2.92E-05
PTPRD	-2.75	-6.74	6.43E-06
TMOD3	-2.75	-6.74	2.10E-05
BCAS3	-2.75	-6.75	0.0001878
NUMB	-2.75	-6.75	3.05E-05
INSIG1	-2.76	-6.76	2.59E-06
MLLT10	-2.76	-6.76	9.95E-05
ITPR3	-2.76	-6.77	2.60E-05
KDM2B	-2.76	-6.77	8.11E-06
PENK	-2.76	-6.77	1.35E-08
ALG1	-2.76	-6.78	0.001114
BEND3	-2.76	-6.78	0.000451
SLC12A4	-2.76	-6.78	7.53E-06
UBA6	-2.76	-6.78	0.001901
ABHD4	-2.76	-6.79	7.03E-05
CAV2	-2.76	-6.79	0.000151
DYNC1H1	-2.76	-6.79	1.39E-05
LONP1	-2.76	-6.79	0.0008547
MSANTD2	-2.76	-6.79	0.001696
RPP25L	-2.76	-6.79	0.003589
TFDP2	-2.76	-6.79	0.0004825
NAV2	-2.76	-6.79	3.61E-05

NDC80	-2.76	-6.79	4.20E-05
LAMP2	-2.77	-6.80	0.0001355
MPP7	-2.77	-6.80	2.80E-05
SGK1	-2.77	-6.80	0.0005245
DNAJB9	-2.77	-6.81	0.001286
KIF26A	-2.77	-6.81	0.0001252
BACH1	-2.77	-6.82	0.0004507
GEN1	-2.77	-6.82	0.0005672
SNN	-2.77	-6.82	0.0002035
ESYT2	-2.77	-6.83	0.0001205
EXOSC9	-2.77	-6.83	3.95E-05
HLF	-2.77	-6.83	0.0003171
MARS2	-2.77	-6.83	0.000279
NCAPH	-2.77	-6.83	1.02E-05
UBE4B	-2.77	-6.83	5.49E-05
WBP2	-2.77	-6.83	3.52E-05
PRELP	-2.77	-6.84	0.0002607
TFB2M	-2.77	-6.84	0.0002985
ARL16	-2.78	-6.85	9.18E-05
NABP1	-2.78	-6.85	0.0004031
POLR3H	-2.78	-6.85	8.35E-05
RAB35	-2.78	-6.85	0.003517
WDR35	-2.78	-6.85	2.75E-05
CDC23	-2.78	-6.86	6.41E-06
CENPJ	-2.78	-6.86	1.45E-05
LAMA1	-2.78	-6.86	3.51E-06
LSM14A	-2.78	-6.86	0.0005028
SRPK2	-2.78	-6.86	0.0002476
GSDME	-2.78	-6.87	0.001004
IARS	-2.78	-6.87	3.91E-05
NBAS	-2.78	-6.87	2.39E-05
UBE2K	-2.78	-6.87	0.0003131
ABHD17B	-2.78	-6.88	0.001364
DDX6	-2.78	-6.88	1.54E-05
DPH1	-2.78	-6.88	7.88E-05
MCM3	-2.78	-6.88	7.31E-06
RACGAP1	-2.78	-6.88	1.25E-05
RBM15B	-2.78	-6.88	0.001815
VPS11	-2.78	-6.88	0.002866
ARFGEF2	-2.78	-6.89	1.29E-05
ARHGAP10	-2.78	-6.89	0.003902
ATF7IP	-2.78	-6.89	4.35E-05
CCNB2	-2.78	-6.89	7.05E-06
DCAF4	-2.78	-6.89	0.0004943
UBE2QL1	-2.78	-6.89	0.0003105
ARID1B	-2.79	-6.90	0.0002797
BRCA2	-2.79	-6.90	6.66E-06
MAML1	-2.79	-6.90	0.004384
PPP1R21	-2.79	-6.90	0.002656
MTFR2	-2.79	-6.90	0.0003323

ALG2	-2.79	-6.91	0.003453
CD36	-2.79	-6.91	1.65E-07
KRT19	-2.79	-6.91	1.53E-05
TARDBP	-2.79	-6.92	7.23E-05
ZFYVE21	-2.79	-6.92	0.001374
PRORSD1P	-2.79	-6.93	0.004203
SETD5	-2.79	-6.93	0.0002184
DEPDC1B	-2.80	-6.94	9.20E-05
GPC4	-2.80	-6.94	6.23E-05
PHLDB2	-2.80	-6.94	6.68E-06
PRPF8	-2.80	-6.94	7.82E-06
SKIV2L2	-2.80	-6.94	0.0001196
CBLL1	-2.80	-6.95	0.001027
CLCN4	-2.80	-6.95	0.0002093
FANCF	-2.80	-6.95	6.19E-06
GPATCH2L	-2.80	-6.95	0.0001574
SEC22A	-2.80	-6.95	0.001544
SPATS2L	-2.80	-6.95	0.0001397
TMEM26	-2.80	-6.95	0.0002288
USO1	-2.80	-6.95	1.29E-05
GATD1	-2.80	-6.96	0.0005406
LIG3	-2.80	-6.96	0.0002042
MED18	-2.80	-6.96	0.002664
AGL	-2.80	-6.97	1.44E-07
ARID5B	-2.80	-6.97	0.0002014
CENPN	-2.80	-6.97	8.54E-05
CUL4A	-2.80	-6.97	0.002673
PACSIN2	-2.80	-6.97	0.0003612
THBS1	-2.80	-6.97	3.23E-07
ZHX1	-2.80	-6.97	0.002614
NDE1	-2.80	-6.98	0.0001111
ITGA6	-2.80	-6.99	3.09E-06
MTRF1L	-2.80	-6.99	0.0005141
LSS	-2.81	-7.00	8.50E-06
SNIP1	-2.81	-7.00	0.001162
SRPX2	-2.81	-7.00	4.85E-07
CCNA2	-2.81	-7.01	5.15E-05
GLDC	-2.81	-7.01	0.0003232
PATJ	-2.81	-7.01	0.001485
SMAD3	-2.81	-7.01	4.26E-05
TMLHE	-2.81	-7.01	0.0001554
AQR	-2.81	-7.02	5.62E-05
DUSP6	-2.81	-7.02	9.21E-06
NUP85	-2.81	-7.02	0.0002727
SRL	-2.81	-7.02	1.98E-09
ABCB10	-2.81	-7.03	7.00E-05
RND3	-2.81	-7.03	0.0001536
RYBP	-2.81	-7.03	0.001152
TTK	-2.81	-7.03	5.96E-06
ATF2	-2.81	-7.04	0.0003923

SOCS5	-2.82	-7.05	1.15E-05
TMEM171	-2.82	-7.05	0.0001427
MYBBP1A	-2.82	-7.05	7.16E-07
EYA2	-2.82	-7.06	0.001294
LEO1	-2.82	-7.06	2.64E-05
SLC25A46	-2.82	-7.06	0.0001024
ZCRB1	-2.82	-7.06	0.004148
BDP1L	-2.82	-7.07	0.0005104
DIRC2	-2.82	-7.07	2.31E-05
RABGGTB	-2.82	-7.07	1.07E-05
TTC30B	-2.82	-7.07	0.0009576
GARNL3	-2.82	-7.08	0.0002701
PELI1	-2.82	-7.08	0.0003164
REST	-2.82	-7.08	0.0007327
RNF111	-2.82	-7.08	0.0006152
STRBP	-2.82	-7.08	0.0001488
TBC1D23	-2.82	-7.08	7.59E-07
VIP	-2.82	-7.08	1.24E-07
WASHC4	-2.82	-7.08	5.85E-05
YY1	-2.82	-7.08	0.002466
METTL16	-2.83	-7.09	0.0009659
ARID1A	-2.83	-7.10	0.0006882
BET1	-2.83	-7.10	0.0003909
EMC8	-2.83	-7.10	0.0002233
MXD1	-2.83	-7.10	8.60E-06
NDST2	-2.83	-7.10	0.0004602
PLA2R1	-2.83	-7.10	0.0006214
POP1	-2.83	-7.10	1.95E-06
TASP1	-2.83	-7.10	2.87E-05
ZDHHC14	-2.83	-7.10	0.001407
MON2	-2.83	-7.11	2.15E-06
RBM17	-2.83	-7.11	0.000125
SLC38A1	-2.83	-7.11	7.44E-07
CTCFL	-2.83	-7.12	0.0001549
ITPR2	-2.83	-7.12	8.73E-05
PDPK1	-2.83	-7.12	0.001139
FBXO32	-2.83	-7.13	7.14E-07
KIN	-2.83	-7.13	4.07E-05
NPC1	-2.83	-7.13	0.0001984
PCM1	-2.83	-7.13	0.0007745
MED4	-2.84	-7.15	0.002216
TBK1	-2.84	-7.15	0.0006056
PPRC1	-2.84	-7.17	2.85E-07
UBR3	-2.84	-7.17	0.0003369
ATP1A1	-2.84	-7.18	0.0001645
DCAF7	-2.84	-7.18	0.0004643
HS6ST2	-2.84	-7.18	0.0004798
MTUS1	-2.84	-7.18	2.52E-05
KNTC1	-2.85	-7.19	4.40E-06
MED27	-2.85	-7.19	0.001933

MKX	-2.85	-7.19	7.07E-05
TSEN2	-2.85	-7.19	0.0007605
ECD	-2.85	-7.20	4.01E-05
FAM135A	-2.85	-7.20	2.74E-05
AP1S2	-2.85	-7.21	3.26E-05
BNC1	-2.85	-7.21	2.66E-05
FLNB	-2.85	-7.21	4.13E-07
PRMT3	-2.85	-7.21	3.32E-05
EPRS	-2.85	-7.22	3.74E-07
NDNF	-2.85	-7.22	4.78E-05
PIP4P2	-2.85	-7.22	0.001308
XRCC3	-2.85	-7.22	0.0003445
AHR	-2.85	-7.23	4.70E-06
ARHGAP19	-2.85	-7.23	2.40E-07
KBTBD2	-2.85	-7.23	0.0006397
AMMECR1L	-2.86	-7.24	0.001717
MCU	-2.86	-7.24	7.55E-06
PHF6	-2.86	-7.24	0.002581
ALG11	-2.86	-7.25	0.0009213
DPH5	-2.86	-7.25	0.001425
MTHFD2	-2.86	-7.25	1.70E-06
TIA1	-2.86	-7.25	0.0001621
ATP2A2	-2.86	-7.26	1.14E-05
PI4KA	-2.86	-7.26	7.88E-05
THAP12	-2.86	-7.26	8.77E-06
TRIM24	-2.86	-7.26	6.33E-07
ALG8	-2.86	-7.27	0.0001595
NOVA1	-2.86	-7.27	0.0003354
TARS	-2.86	-7.27	3.49E-05
BRPF3	-2.86	-7.28	0.0001352
FEZ2	-2.86	-7.28	0.000289
SLC7A6OS	-2.86	-7.28	0.0005405
HTRA3	-2.87	-7.29	0.0002491
KIAA0408	-2.87	-7.29	3.97E-05
C9H3orf58	-2.87	-7.30	1.85E-08
DOCK9	-2.87	-7.30	2.66E-05
HDAC9	-2.87	-7.30	6.02E-06
ING3	-2.87	-7.30	0.0002887
MFSD2A	-2.87	-7.30	1.01E-05
CH507-9B2.5	-2.87	-7.31	4.37E-08
CRYBG3	-2.87	-7.31	0.0003814
OXR1	-2.87	-7.31	0.001496
SLC24A5	-2.87	-7.31	1.35E-06
PYROXD1	-2.87	-7.32	0.0001707
SLC2A1	-2.87	-7.32	2.08E-05
ANK3	-2.88	-7.34	0.0004088
LOC420915	-2.88	-7.34	3.97E-07
NPAT	-2.88	-7.34	6.28E-06
RECQL	-2.88	-7.34	6.30E-05
SMC3	-2.88	-7.34	6.91E-05

CWC25	-2.88	-7.35	2.55E-05
HTATSF1	-2.88	-7.35	0.0001563
KLF3	-2.88	-7.35	4.01E-05
NRP1	-2.88	-7.35	2.92E-06
CDC37L1	-2.88	-7.36	0.0001071
MTMR3	-2.88	-7.36	0.0007754
SLC30A1	-2.88	-7.36	0.0005064
C7H2ORF69	-2.88	-7.37	0.0001135
FBXO3	-2.88	-7.37	7.03E-05
METAP1	-2.88	-7.37	7.35E-07
PNRC2	-2.88	-7.38	7.86E-05
GARS	-2.89	-7.39	4.76E-05
GRAMD1C	-2.89	-7.39	9.25E-05
GTF2H2	-2.89	-7.39	0.0001062
INTS7	-2.89	-7.39	2.13E-05
KIF2C	-2.89	-7.39	7.82E-06
APCDD1L	-2.89	-7.40	4.30E-06
DSCC1	-2.89	-7.40	1.98E-05
FKBP5	-2.89	-7.40	0.000586
JADE3	-2.89	-7.40	7.20E-05
NOL10	-2.89	-7.40	9.24E-06
TOM1L2	-2.89	-7.40	0.0002807
CENPI	-2.89	-7.41	3.01E-08
DCUN1D1	-2.89	-7.41	0.0001684
MPDZ	-2.89	-7.41	7.23E-05
NDEL1	-2.89	-7.41	0.0002036
RIC1	-2.89	-7.41	0.0008321
SPRY2	-2.89	-7.41	5.38E-06
TICAM1	-2.89	-7.41	0.0009297
TNFAIP8L1	-2.89	-7.41	1.11E-05
TRIM37	-2.89	-7.41	9.97E-06
PCGF6	-2.89	-7.42	0.0003547
C5H14orf4	-2.89	-7.43	0.0001724
DTNBP1	-2.89	-7.43	0.0001335
LACTB2	-2.90	-7.45	0.0006108
MYNN	-2.90	-7.45	0.0006287
NCAPG2	-2.90	-7.45	5.17E-06
TXNRD2	-2.90	-7.45	6.14E-05
ATAD2	-2.90	-7.46	1.70E-05
DGCR2	-2.90	-7.46	4.58E-05
TRMT10C	-2.90	-7.46	0.0001913
ACO1	-2.90	-7.47	1.73E-05
GINS3	-2.90	-7.47	0.001724
RELCH	-2.90	-7.47	5.27E-06
TUBB4B	-2.90	-7.47	0.000112
GOSR1	-2.90	-7.48	0.0009765
LMTK2	-2.90	-7.48	9.41E-05
MSH2	-2.90	-7.48	1.35E-05
SLC38A2	-2.90	-7.48	2.23E-10
ZNF639	-2.90	-7.48	0.00102

PGP	-2.91	-7.50	3.94E-05
CLTC	-2.91	-7.51	1.60E-05
DENND2A	-2.91	-7.51	1.56E-05
APPL2	-2.91	-7.52	1.07E-05
FAM83D	-2.91	-7.52	4.91E-07
HAUS8	-2.91	-7.52	3.03E-05
IVNS1ABP	-2.91	-7.52	4.16E-05
PPP1R3B	-2.91	-7.52	0.0001784
TMEM177	-2.91	-7.52	0.0002227
NOTCH1	-2.91	-7.53	7.88E-06
UBXN2B	-2.91	-7.54	5.13E-05
ARGLU1	-2.92	-7.55	1.47E-05
FNDC3B	-2.92	-7.55	2.56E-07
NECTIN3	-2.92	-7.55	1.02E-05
PLCE1	-2.92	-7.55	5.25E-06
INTS1	-2.92	-7.57	6.58E-06
NUP155	-2.92	-7.57	4.06E-06
TECPR2	-2.92	-7.57	0.0002049
CITED4	-2.92	-7.59	3.85E-06
CNOT10	-2.92	-7.59	7.35E-05
MAP4K3	-2.92	-7.59	0.0002099
SIPA1L1	-2.92	-7.59	6.06E-05
ETAA1	-2.93	-7.60	7.04E-05
FAM76B	-2.93	-7.60	5.08E-06
HTRA1	-2.93	-7.60	5.29E-07
TTC7B	-2.93	-7.62	2.89E-05
CHORDC1	-2.93	-7.63	2.76E-06
KLHL20	-2.93	-7.63	0.0002324
ATP5A1Z	-2.94	-7.65	1.26E-06
BTRC	-2.94	-7.65	5.25E-05
CDC42EP3	-2.94	-7.65	3.04E-05
PEX1	-2.94	-7.65	6.71E-06
SPATA7	-2.94	-7.67	4.28E-06
ADM	-2.94	-7.69	9.36E-06
HNRNPDL	-2.94	-7.69	1.42E-05
PDCD11	-2.94	-7.69	7.85E-06
FLVCR1	-2.95	-7.71	1.25E-05
PLK2	-2.95	-7.71	1.17E-06
PTPN13	-2.95	-7.71	1.69E-06
VCL	-2.95	-7.71	7.56E-09
RAB40B	-2.95	-7.72	0.001347
PRPF4B	-2.95	-7.73	4.32E-05
WDR11	-2.95	-7.73	3.57E-05
YARS2	-2.95	-7.73	1.47E-05
PPHLN1	-2.95	-7.74	5.69E-06
PPP1R8	-2.95	-7.74	5.62E-05
MELK	-2.95	-7.75	2.52E-06
OSBPL6	-2.95	-7.75	1.38E-05
DACT1	-2.96	-7.76	4.15E-08
HERC4	-2.96	-7.76	0.0002036

KLHL26	-2.96	-7.77	2.74E-05
NPR3	-2.96	-7.79	3.03E-08
RHOB	-2.96	-7.79	0.0001052
TOE1	-2.96	-7.79	1.30E-05
ID4	-2.96	-7.81	2.65E-06
PDZRN3	-2.96	-7.81	2.18E-07
PRKCB	-2.96	-7.81	0.0008867
TGIF1	-2.96	-7.81	9.24E-07
SUPT7L	-2.97	-7.82	8.03E-06
CENPL	-2.97	-7.83	2.82E-05
ISM1	-2.97	-7.83	1.83E-07
SRSF5	-2.97	-7.83	0.0001277
FOXN3	-2.97	-7.84	0.0001932
LONP2	-2.97	-7.84	9.24E-05
MFSD14B	-2.97	-7.84	1.41E-05
TRIM59	-2.97	-7.84	0.0002493
DNAAF5	-2.97	-7.85	1.30E-06
E2F8	-2.97	-7.85	5.90E-06
NOC3L	-2.97	-7.85	1.09E-06
NOV	-2.97	-7.85	0.000218
SFRP2	-2.97	-7.85	4.83E-09
ETV5	-2.98	-7.88	6.43E-05
MIR1600	-2.98	-7.89	1.13E-05
LIPG	-2.98	-7.90	3.81E-06
PINK1	-2.98	-7.90	7.33E-06
CLK3	-2.98	-7.91	2.65E-05
INTS4	-2.98	-7.91	0.0001727
LRRC49	-2.98	-7.91	3.26E-05
PCSK5	-2.99	-7.92	4.00E-06
RMI1	-2.99	-7.92	0.0003206
SGO1	-2.99	-7.94	6.95E-08
HMGCS1	-2.99	-7.95	3.30E-06
RGS2	-2.99	-7.95	9.31E-07
DCP1B	-2.99	-7.97	2.75E-05
ESS2	-2.99	-7.97	0.0002158
ICE1	-2.99	-7.97	9.65E-06
SPART	-2.99	-7.97	1.60E-05
TNFRSF1A	-2.99	-7.97	0.0002494
RAB22A	-3.00	-7.98	6.61E-05
ZBED1	-3.00	-7.98	1.50E-05
MYH10	-3.00	-7.99	1.16E-06
LETM2	-3.00	-8.00	9.27E-06
RMND5A	-3.00	-8.00	0.0002009
RTTN	-3.00	-8.00	3.82E-07
ZFYVE26	-3.00	-8.00	1.33E-06
NFYA	-3.00	-8.01	4.71E-06
HMGB2	-3.00	-8.02	4.99E-07
ANKRD13A	-3.01	-8.04	6.12E-06
MTX3	-3.01	-8.06	4.15E-06
RRP7A	-3.01	-8.07	3.11E-05

FDPS	-3.01	-8.08	1.33E-05
HSD17B7	-3.01	-8.08	6.09E-06
ARMC6	-3.02	-8.11	1.19E-05
COQ8B	-3.02	-8.11	5.67E-06
DAB2IP	-3.02	-8.11	1.73E-05
FBXL5	-3.02	-8.11	3.63E-05
HMGCR	-3.02	-8.12	4.58E-09
SGPL1	-3.02	-8.12	0.000101
ACTR5	-3.02	-8.13	1.96E-06
ITGA9	-3.02	-8.13	8.56E-06
VCAN	-3.02	-8.13	2.32E-07
CPNE3	-3.03	-8.14	2.22E-07
FGFBP1	-3.03	-8.15	5.36E-07
PTPN14	-3.03	-8.16	2.50E-06
PDP1	-3.03	-8.18	4.03E-05
FDFT1	-3.03	-8.19	1.58E-07
IARS2	-3.04	-8.20	8.24E-05
CRLF1	-3.04	-8.21	2.72E-06
CSF1R	-3.04	-8.21	1.71E-12
GLT1D1	-3.04	-8.21	1.47E-05
RSF1	-3.04	-8.24	5.99E-07
SOCS4	-3.04	-8.24	2.59E-06
ACAP2	-3.04	-8.25	2.06E-05
IREB2	-3.04	-8.25	1.35E-05
EIF2AK3	-3.05	-8.27	1.89E-09
FCHSD2	-3.05	-8.27	4.14E-07
MED14	-3.05	-8.27	1.02E-05
FZD3	-3.05	-8.28	4.01E-06
SPATA13	-3.05	-8.28	6.32E-06
ATR	-3.05	-8.30	2.54E-08
NSDHL	-3.05	-8.30	4.33E-06
BCAR1	-3.06	-8.32	7.78E-07
BTBD3	-3.06	-8.32	1.53E-05
FGFRL1	-3.06	-8.32	1.56E-05
MAN1A1	-3.06	-8.33	9.90E-06
NOM1	-3.06	-8.35	8.65E-06
SEC16A	-3.06	-8.35	5.67E-06
MYC	-3.07	-8.37	4.14E-06
CEBPB	-3.07	-8.38	5.19E-06
LRCH2	-3.07	-8.38	6.09E-07
SEC22C	-3.07	-8.38	2.96E-05
AP4E1	-3.07	-8.42	1.54E-07
TOPBP1	-3.08	-8.43	2.60E-06
HAGHL	-3.08	-8.44	4.47E-06
NSRP1	-3.09	-8.52	2.97E-05
SYDE2	-3.09	-8.52	1.38E-05
BMP2K	-3.09	-8.53	3.21E-05
ERICH1	-3.10	-8.55	1.06E-06
NUP133	-3.10	-8.55	4.76E-07
STAGL	-3.10	-8.55	1.15E-05

IRF2BP2	-3.10	-8.56	4.44E-06
AKTIP	-3.10	-8.57	3.88E-05
CCDC93	-3.10	-8.57	2.92E-06
ARHGEF10	-3.10	-8.58	2.34E-06
SLC25A13	-3.10	-8.58	6.82E-08
PEX5	-3.10	-8.60	0.0001286
BAMBI	-3.11	-8.61	6.62E-05
CDC20	-3.11	-8.61	2.80E-07
LPL	-3.11	-8.61	9.59E-08
MTOR	-3.11	-8.61	4.84E-06
HJURP	-3.11	-8.65	7.49E-07
NLK	-3.11	-8.65	9.11E-06
URB2	-3.11	-8.65	5.25E-08
EBP	-3.11	-8.66	2.16E-07
DCBLD1	-3.12	-8.67	1.17E-06
MICAL2	-3.12	-8.67	1.71E-08
RFTN1	-3.12	-8.69	2.70E-05
DHCR7	-3.12	-8.71	9.63E-06
CLCN6	-3.13	-8.73	7.75E-06
IDI1	-3.13	-8.73	1.84E-06
FASTKD3	-3.13	-8.74	6.11E-07
SNX30	-3.13	-8.74	1.72E-05
EDEM3	-3.13	-8.75	1.36E-05
WEE1	-3.13	-8.75	2.32E-06
JMJD7	-3.13	-8.77	1.56E-05
NUP93	-3.14	-8.79	2.29E-07
RCC1L	-3.14	-8.79	1.87E-06
SLC12A2	-3.14	-8.79	1.94E-07
FRMD4B	-3.14	-8.82	2.04E-05
MED17	-3.14	-8.83	1.19E-05
FBXL3	-3.14	-8.85	1.61E-05
ANAPC7	-3.15	-8.86	1.81E-06
TMF1	-3.15	-8.86	9.18E-06
DIP2C	-3.15	-8.87	8.41E-07
ANTXR1	-3.15	-8.90	2.39E-06
VPS13C	-3.15	-8.90	2.34E-06
HMOX2	-3.16	-8.91	5.25E-06
IBSP	-3.16	-8.91	3.97E-09
PRR11	-3.16	-8.94	6.74E-08
PTCH1	-3.16	-8.95	7.24E-08
MARCH5	-3.16	-8.97	4.67E-05
ANKS1B	-3.17	-8.99	2.35E-05
EAPP	-3.18	-9.04	7.40E-06
SLC1A4	-3.18	-9.05	7.33E-08
STAM	-3.18	-9.06	8.78E-08
GPR146	-3.18	-9.09	6.41E-08
TP53RK	-3.19	-9.10	2.32E-05
AR	-3.19	-9.12	1.51E-05
SYNE3	-3.20	-9.16	4.11E-06
FBXL21	-3.21	-9.26	2.97E-06

LMNB1	-3.21	-9.26	9.35E-08
DVL1	-3.22	-9.30	2.38E-06
BTBD11	-3.22	-9.33	2.66E-06
ZYG11B	-3.22	-9.33	3.35E-06
METTL14	-3.22	-9.35	1.18E-06
DAPK1	-3.23	-9.36	2.21E-06
RAB3GAP2	-3.23	-9.37	2.02E-08
HEATR5B	-3.23	-9.40	5.84E-07
BARD1	-3.24	-9.43	4.25E-08
KLF2	-3.24	-9.46	5.69E-07
ARL4A	-3.24	-9.48	5.55E-08
TRRAP	-3.25	-9.49	4.65E-09
SQLE	-3.25	-9.54	5.62E-07
TMEM181	-3.25	-9.54	1.06E-05
LDLR	-3.26	-9.58	2.81E-08
ISY1	-3.26	-9.60	1.05E-05
MTMR12	-3.27	-9.64	9.19E-07
DUS3L	-3.27	-9.67	3.20E-07
CDR2	-3.28	-9.69	1.75E-06
BTG1	-3.29	-9.75	6.55E-06
FZD8	-3.29	-9.78	1.70E-08
MIS12	-3.29	-9.80	9.59E-08
ZBTB5	-3.30	-9.83	3.85E-07
AMOTL1	-3.30	-9.86	3.05E-07
CIT	-3.30	-9.86	7.29E-08
AURKA	-3.30	-9.87	2.42E-08
TSHZ3	-3.31	-9.89	7.18E-07
CBS	-3.31	-9.90	1.70E-07
ZBTB8B	-3.31	-9.90	4.43E-07
TEX10	-3.31	-9.92	2.49E-06
SLU7	-3.32	-9.97	2.45E-06
USP6NL	-3.32	-9.98	2.49E-06
EPHA4	-3.32	-10.02	2.66E-08
CYR61	-3.33	-10.06	3.48E-07
PLEKHA5	-3.34	-10.16	2.77E-10
USP32	-3.37	-10.31	4.53E-07
KPNA2	-3.37	-10.33	9.68E-09
LGR4	-3.37	-10.33	1.64E-06
LIG4	-3.38	-10.41	4.10E-07
PLK1	-3.38	-10.41	2.82E-09
PRC1	-3.38	-10.43	6.41E-10
SLC4A11	-3.38	-10.43	8.11E-09
ZCCHC8	-3.39	-10.50	8.23E-09
CIRBP	-3.40	-10.56	3.09E-09
TOP2A	-3.40	-10.58	6.52E-11
CENPC	-3.42	-10.67	1.22E-08
KIF18A	-3.42	-10.67	5.76E-09
NUSAP1	-3.42	-10.67	2.90E-08
TRIM45	-3.42	-10.72	3.72E-08
SEC24B	-3.42	-10.74	2.82E-07

ARHGAP11A	-3.43	-10.77	2.77E-09
TTF2	-3.43	-10.77	2.18E-09
SSH1	-3.45	-10.90	6.47E-08
CCNB3	-3.45	-10.95	1.26E-08
NR2C1	-3.45	-10.95	9.71E-08
FAM98A	-3.47	-11.05	1.04E-07
INCENP	-3.47	-11.05	2.66E-09
NFIL3	-3.47	-11.08	9.64E-09
EP300	-3.50	-11.29	9.39E-08
LOC395676	-3.51	-11.38	1.72E-08
LUC7L3	-3.51	-11.38	2.15E-11
IQSEC1	-3.53	-11.52	4.07E-07
PPP1R3C	-3.53	-11.58	3.91E-12
VLDLR	-3.55	-11.72	9.04E-10
FAM20C	-3.56	-11.80	1.71E-05
STK17B	-3.57	-11.88	1.30E-07
STARD4	-3.57	-11.90	2.19E-09
MYH1D	-3.62	-12.32	7.50E-16
AACS	-3.64	-12.42	2.71E-09
DHCR24	-3.64	-12.45	1.51E-09
LRIG2	-3.65	-12.58	1.88E-08
SPTY2D1	-3.66	-12.64	1.80E-08
HASPIN	-3.69	-12.91	8.65E-11
FASN	-3.71	-13.05	1.34E-10
RERE	-3.72	-13.22	1.39E-09
CTGF	-3.75	-13.41	4.24E-08
AACS	-3.78	-13.78	5.11E-11
ADNP2	-3.81	-14.03	2.07E-09
GTF3C3	-3.82	-14.11	3.27E-10
PKDCCA	-3.88	-14.69	1.36E-09
KIF20A	-3.90	-14.89	1.18E-11
FOXO1	-3.94	-15.31	2.19E-10
FAM72A	-3.96	-15.52	2.80E-11
LIN9	-3.99	-15.94	9.39E-11
ROR1	-4.01	-16.06	7.04E-11
KIF11	-4.03	-16.37	9.45E-14
BUB1B	-4.11	-17.26	9.77E-12
BBS12	-4.49	-22.53	2.48E-13
KIAA1210	-4.53	-23.03	3.66E-12
MKI67	-4.55	-23.48	9.92E-15
CHAC1	-4.79	-27.60	1.90E-15
PRPSAP1	-4.90	-29.87	6.67E-16

Supplementary Table S4: Differentially expressed genes from HPAI data analysis

Gene	Log2 Fold change	Fold change	p-value
MTHFD2	2.29	4.89	4.49E-05
ROPN1L	1.75	3.37	0.0001451
LOC417056	1.73	3.32	8.65E-06
REG1A	1.66	3.15	0.0001049
ZBTB16	1.65	3.13	3.89E-05
MC2R	1.64	3.11	0.0001104
BF2	1.61	3.04	4.88E-05
COL10A1	1.60	3.03	4.13E-05
CXCL14	1.58	3.00	0.0001313
EOGT	1.58	3.00	4.23E-05
ANGPT1	1.53	2.89	5.61E-05
CENPS	1.53	2.89	6.96E-05
NPY	1.52	2.86	3.28E-05
LOC418667	1.51	2.85	0.0001471
CHRNA6	1.50	2.84	0.0001087
COTL1	1.50	2.83	0.000129
AvBD7	1.49	2.80	1.31E-05
RNASE4	1.49	2.80	7.36E-05
GNLY	1.49	2.80	0.0001077
LOC100859715	1.48	2.79	0.0001443
ATP8A1	1.48	2.79	0.0001257
DUSP4	1.47	2.78	3.60E-05
MPEG1	1.47	2.78	7.06E-05
ERFE	1.46	2.76	0.0001382
CPNE4	1.46	2.76	1.30E-06
DDX60	1.46	2.76	9.46E-05
NT5C3B	1.46	2.75	0.0001143
LY96	1.44	2.71	6.40E-05
CCK	1.43	2.70	1.10E-05
HIST1H46	1.43	2.69	0.0001126
NCOA2	1.42	2.68	8.50E-07
DMBT1	1.42	2.67	0.0001478
XPO1	1.42	2.67	9.01E-05
KIF2A	1.42	2.67	6.59E-05
EPSTI1	1.41	2.66	2.26E-05
HERC4L	1.40	2.64	6.37E-05
RNF213	1.40	2.64	3.95E-05
GIMAP8L2	1.40	2.63	5.48E-06
C3H8ORF80	1.40	2.63	7.89E-05
LOC100857928	1.40	2.63	5.09E-05
MINDY3	1.39	2.62	7.73E-05
CMTR1	1.39	2.62	6.28E-05
RSAD2	1.38	2.60	1.71E-05
ETV7	1.38	2.60	7.83E-05
LOC415325	1.38	2.60	2.16E-06
CXorf21	1.37	2.59	4.25E-06
FANCA	1.37	2.59	6.70E-06
IFIT5	1.37	2.58	4.29E-05
CATH1	1.37	2.58	1.17E-06
MADPRT1	1.35	2.55	1.26E-05
INHBB	1.35	2.55	0.0001154
C11H19orf12	1.35	2.55	4.28E-05

COL9A3	1.35	2.55	9.24E-06
GCH1	1.35	2.55	7.82E-06
IRF7	1.34	2.54	4.76E-05
DESI2L	1.34	2.52	2.00E-05
CRISPLD2	1.33	2.51	3.90E-05
MX1	1.33	2.51	6.44E-07
CALHM6	1.33	2.51	6.36E-06
PRDM12	1.32	2.50	0.000144
IFNW1	1.31	2.48	2.12E-05
CCAH221	1.31	2.47	8.29E-08
PMAIP1	1.31	2.47	7.07E-05
IL17REL	1.30	2.46	7.90E-05
SPTBN5	1.30	2.46	1.23E-06
SAMD9L	1.28	2.44	5.71E-06
GVINP1	1.28	2.43	7.28E-07
CMPK2	1.26	2.40	2.53E-07
BATF3	1.26	2.39	1.12E-05
LOC420107	1.26	2.39	6.11E-07
AKR	1.25	2.39	7.72E-07
IFI27L1	1.25	2.38	3.32E-07
SRI	1.25	2.38	5.46E-07
PLACL2	1.25	2.37	5.58E-07
LYGL	1.24	2.36	3.50E-07
CCL4	1.23	2.35	4.29E-07
USP18	1.23	2.35	6.81E-08
CCL19	1.23	2.35	5.59E-08
TOR1BL	1.23	2.35	1.43E-07
SLC17A6	1.22	2.33	0.0001009
OASL	1.22	2.32	1.75E-08
AVD	1.21	2.32	1.99E-10
LOC415756	1.21	2.32	2.35E-07
F3	1.18	2.27	3.05E-06
IL6	1.16	2.23	3.06E-07
IFI6	1.15	2.23	7.06E-08
IL8L2	1.15	2.22	1.38E-06
K123	1.15	2.21	5.84E-08
LOC101750271	1.14	2.21	1.18E-08
SMKR1	-1.55	-2.93	0.0003545
CYP3A5	-1.74	-3.34	0.0002409
FOXJ1	-1.75	-3.37	0.000331
DNAH9	-1.95	-3.85	0.000104
PACRG	-1.97	-3.91	0.0001524
PIGR	-2.03	-4.08	0.0001606
RSPH1	-2.03	-4.09	3.71E-05
DYDC1	-2.06	-4.16	1.10E-05
AK7	-2.08	-4.22	7.56E-05
C11orf88	-2.16	-4.46	4.75E-05
BLB1	-2.16	-4.46	0.0003432
CSTA	-2.19	-4.55	0.0002587
PIH1D3	-2.19	-4.56	0.0001811
TAL1	-2.19	-4.57	6.45E-05
RAB36	-2.20	-4.60	6.10E-05
SNTN	-2.21	-4.64	4.75E-05
CDHR3	-2.25	-4.77	4.51E-05

LRRC6	-2.28	-4.85	0.0001075
ARMC4	-2.33	-5.03	0.000293
ACVR2B	-2.34	-5.07	0.0003331
ADCY8	-2.43	-5.40	0.0001748
CXCR4	-2.44	-5.41	0.0001835
SALL1	-2.44	-5.41	0.000197
MMP13	-2.45	-5.45	0.000142
ENKUR	-2.45	-5.45	4.18E-06
SERPIND1	-2.47	-5.55	0.0001809
KMT5B	-2.48	-5.56	0.0003282
SAMD12	-2.50	-5.64	8.26E-05
RASL11B	-2.51	-5.70	0.0002586
EDN2	-2.52	-5.75	0.00023
MEIS2	-2.53	-5.77	0.0002674
PRTFDC1	-2.53	-5.79	0.0003215
TPH1	-2.54	-5.80	0.000298
PCDH17	-2.59	-6.02	3.26E-05
CGNL1	-2.61	-6.10	9.51E-05
TFPI	-2.62	-6.14	2.57E-05
AP1S3	-2.63	-6.21	0.0001795
CCDC141	-2.66	-6.30	1.96E-05
UBAP2	-2.66	-6.31	0.0002476
SLC9A9	-2.67	-6.37	0.0003191
NPTX2	-2.68	-6.42	0.0003126
DNAH5L	-2.69	-6.45	3.12E-05
SPP1	-2.69	-6.46	9.69E-06
ALKAL2	-2.69	-6.46	3.49E-05
ATP5MF	-2.72	-6.60	0.0001356
RIPPLY2	-2.83	-7.09	6.59E-05
NIPBLL	-2.83	-7.13	8.91E-05
SH3TC1	-2.83	-7.13	2.16E-05
SLC6A2	-2.84	-7.17	4.17E-05
RASGRP3	-2.86	-7.28	8.03E-06
ATP5O	-2.87	-7.32	4.54E-05
BMP2	-2.91	-7.51	0.0001659
UMPS	-2.93	-7.60	1.74E-05
ATP1B3	-3.00	-8.00	0.0001015
NPR3	-3.00	-8.00	8.90E-05
PTGR1	-3.01	-8.05	0.0002977
CHST15	-3.08	-8.47	5.94E-06
OXSR1	-3.16	-8.97	7.75E-05
SMIM3	-3.17	-8.98	0.0003359
PRRG1	-3.23	-9.40	6.60E-06
CLDN1	-3.29	-9.76	0.000122
TNFRSF1B	-3.29	-9.80	5.60E-07
SLC44A1	-3.31	-9.92	3.70E-05
SUCLG2	-3.35	-10.18	6.06E-06
HSP90B1	-3.35	-10.21	0.000226
ZFRL1	-3.35	-10.21	2.28E-05
AGA	-3.37	-10.31	0.0002488
BROX	-3.42	-10.74	1.89E-05
CCNJ	-3.49	-11.23	1.52E-07
USP12	-3.52	-11.46	0.0002076
LOC101750526	-3.54	-11.62	1.81E-07

FBXL4	-3.66	-12.60	3.03E-06
JARID2	-3.67	-12.76	0.0002344
CA4	-3.68	-12.80	3.88E-07
NES	-3.70	-13.01	6.15E-05
ZSWIM6L	-3.76	-13.55	9.66E-05
NLRP1L	-3.79	-13.85	8.50E-06
LOC423277	-3.81	-14.01	7.07E-05
DIEXF	-4.07	-16.83	0.0002332
ORAI1	-4.14	-17.57	0.0001822
RAMP3	-4.15	-17.71	3.85E-06
DISP1	-4.18	-18.11	0.0001241
DUSP1	-4.18	-18.14	0.0001943
KIAA1107	-4.23	-18.70	0.0001746
EEPD1	-4.26	-19.17	5.05E-06
KCNMA1	-4.37	-20.74	0.0001486
RCBTB2	-4.39	-21.00	0.0001654
TLR5	-4.47	-22.15	1.33E-07
AQR	-4.49	-22.48	0.0002056
AASDHPPT	-4.52	-22.98	3.49E-05
MYO10	-4.62	-24.54	1.63E-05
ROCK2	-4.63	-24.82	5.27E-05
SLC4A11	-4.79	-27.73	2.81E-05
LOC431003	-4.82	-28.24	2.93E-06
F2R	-4.87	-29.25	9.71E-05
TAMM41	-4.87	-29.32	0.0001184
NR2C1	-4.89	-29.66	9.85E-05
XRCC3	-4.93	-30.51	0.0001746
NUP98	-4.99	-31.78	0.0002212
BTF3L	-5.13	-35.11	7.70E-07
PARD6G	-5.14	-35.19	0.0002972
HAUS8	-5.16	-35.72	0.0003187
SNX30	-5.18	-36.17	0.0002738
WNT2B	-5.19	-36.62	0.0001501
TMEM26	-5.30	-39.30	2.67E-05
ADRM1	-5.33	-40.31	0.0003117
C4H20ORF194	-5.39	-41.90	1.07E-05
DCBLD1	-5.45	-43.68	5.23E-05
MED27	-5.45	-43.68	0.0002588
TNFAIP8L1	-5.45	-43.68	9.98E-05
CHAC1	-5.56	-47.29	0.0001855
FILIP1L	-5.70	-51.99	2.93E-05
CHDB1	-5.72	-52.56	5.06E-07
HNRNPKL	-5.75	-53.88	6.94E-08
MYH10	-5.77	-54.48	0.0002668
BAMBI	-5.88	-59.09	0.000109
ORMDL3	-5.93	-60.96	0.0002083
KCNQ1	-5.93	-61.14	4.51E-05
CDR2	-5.95	-61.84	0.0001129
COQ8B	-5.96	-62.37	0.000149
GNAI1	-5.99	-63.64	0.0003354
CABIN1	-6.19	-73.24	0.0002594
PANK1	-6.25	-76.14	6.71E-05
LGR4	-6.31	-79.43	0.0002151
EED	-6.34	-80.89	0.0002268

ANKS1B	-6.51	-91.24	0.0002097
LOC100859072	-6.52	-91.81	1.10E-08
FNIP2	-6.56	-94.15	4.91E-06
PPP1R3B	-6.64	-99.67	8.47E-05
CAV2	-6.69	-103.58	1.37E-05
RNF151	-6.77	-109.44	0.0002762
MCCC2L	-6.84	-114.94	4.33E-05
RECQL	-7.00	-128.28	6.82E-06
IVNS1ABP	-7.27	-153.83	3.81E-05
SLC4A7	-7.28	-155.45	8.09E-06
PEX14	-7.30	-157.09	0.000285
HMG20A	-7.48	-178.50	0.0001705
TYRO3	-7.50	-181.09	1.71E-05
ZMYM2	-7.55	-187.76	0.0001878
UHRF1	-7.91	-240.81	5.92E-05
ARHGAP24	-7.97	-251.14	0.0001712
PAPD7	-7.99	-254.04	0.0001755
ST5	-8.13	-280.96	0.0002267
SPIN1W	-8.17	-288.77	4.83E-08
FAM107B	-8.54	-371.86	7.17E-05
NDNF	-8.61	-390.70	0.0001072
CEP192	-8.66	-405.57	0.0002991
METTL14	-8.84	-458.16	6.41E-05
MIER3L	-9.15	-568.67	1.31E-07
DTNBP1	-9.16	-573.70	0.0001237
BET1	-9.45	-701.59	0.0001991
FAM98A	-9.67	-816.46	0.0001907
TXNL1	-9.79	-888.17	1.81E-07
CRIPT	-10.28	-1244.76	0.0002993
ABHD17B	-10.36	-1314.54	3.76E-05
TP53RK	-10.44	-1388.82	3.45E-05
ZYG11B	-10.97	-2011.24	0.0003324
FAM20C	-11.08	-2166.09	0.0002585
BPGM	-11.16	-2284.95	2.77E-06
KIAA1210	-11.18	-2322.04	2.15E-05
PDGFRA	-11.66	-3227.03	8.82E-05
STAM	-11.87	-3737.76	4.26E-05
KLF2	-11.98	-4026.70	1.23E-06
SPRED2	-12.70	-6663.86	6.95E-05
TLK1L	-12.76	-6912.92	2.28E-05
IREB2	-12.98	-8068.22	4.23E-05
GEM	-13.46	-11297.15	2.55E-06
IRF2BP2	-13.49	-11518.76	0.0002428
FOXO1	-14.05	-16904.84	3.75E-05
STK17B	-15.31	-40502.70	1.33E-05
PKDCCA	-17.32	-163122.28	4.36E-07
CTGF	-18.34	-332039.41	2.49E-06
MARCH5	-18.56	-386040.98	4.32E-05
FGFBP1	-18.69	-422174.03	8.98E-06
IQSEC1	-22.72	-6917013.00	1.39E-05
HINTW	-150.44	###################	1.62E-11

Supplementary Table S5: Gene Ontology of up-regulated HPAI DEGs

source	Term name	Adjusted p value	Intersection size
GO:MF[1]	receptor ligand	0.000176	11
GO:MF	signaling receptor	0.000199	11
GO:MF	receptor regulator	0.000352	11
GO:BP[2]	response to external	8.15E-06	26
GO:BP	response to other	2.66E-08	21
GO:BP	response to external	2.81E-08	21
GO:BP	response to biotic	4.73E-08	21
GO:BP	defense response	2.10E-07	21
GO:BP	interspecies	2.76E-06	21
GO:CC[3]	extracellular space	0.000532	21
GO:CC	extracellular region	0.000145	18
KEGG	Influenza A	0.00044	7
KEGG	Cytokine-cytokine	0.033055	6
KEGG	NOD-like receptor	0.030848	5
KEGG	Toll-like receptor	0.004656	5
REAC[4]	Immune System	0.000973	20
REAC	Innate Immune	0.024546	13
TF[5]	Factor: IRF; motif:	0.004008	20
TF	Factor: IRF; motif:	0.002202	16
TF	Factor: IRF; motif:	0.003641	14
TF	Factor: ICSBP; motif:	0.004731	14
TF	Factor: IRF-1; motif:	0.00793	10

1. Gene Ontology Molecular Function

2. Gene Ontology Biological Process

3. Gene Ontology Cellular Component

4. Reactome

5. Transcription factors

Supplementary Table S6: Gene Ontology of down-regulated HPAI DEGs

Source	Term name	Adjusted p value	Intersection size
GO:BP[1]	determination of left/right symmetry	0.049035	7
GO:BP	axoneme assembly	0.024977	6
GO:BP	outer dynein arm assembly	0.016589	4
TF[2]	Factor: E2F; motif: GGCGSG	0.049508	113
TF	Factor: Sp1; motif: NGGGGCGGGGN	0.047709	92

[1]Gene Ontology Biological Process

[2] Transcription factors

Supplementary Table S7: Gene Ontology of up-regulated LPAI DEGs

Source	Term name	Adjusted p value	Intersection size
GO:MF[1]	identical protein binding	0.020594	15
GO:BP[2]	positive regulation of biological process	0.000187	37
GO:BP	positive regulation of cellular process	0.001194	34
GO:BP	gene expression	0.002064	34
GO:BP	positive regulation of metabolic process	0.000212	29
GO:BP	positive regulation of macromolecule metabolic process	0.000179	28
GO:BP	regulation of gene expression	0.019466	28
GO:BP	positive regulation of nitrogen compound metabolic process	0.000545	26
GO:CC[3]	side of membrane	0.030987	9
GO:CC	IkappaB kinase complex	0.015049	2
GO:CC	macrophage migration inhibitory factor receptor complex	0.005031	2
KEGG	Herpes simplex virus 1 infection	1.06E-08	12
KEGG	Cell adhesion molecules	5.19E-05	8
KEGG	MAPK signaling pathway	0.016731	8
KEGG	ErbB signaling pathway	0.000695	6
KEGG	Phagosome	0.013416	6
KEGG	Cellular senescence	0.02002	6
REAC[4]	Immune System	3.67E-06	22
REAC	Innate Immune System	0.008822	13
REAC	Adaptive Immune System	0.005621	11
WP[5]	FAS pathway and Stress induction of HSP regulation	0.000262	5
WP	NLR Proteins	0.040351	2
TF[6]	Factor: HSF2; motif: NGAANNWTCK	0.023439	31

1. Gene Ontology Molecular Function
2. Gene Ontology Biological Process
3. Gene Ontology Cellular Component
4. Reactome
5. Wiki Pathways
6. Transcription factors

Supplementary Table S8: Gene Ontology of down-regulated LPAI DEGs

Source	Term name	Adjusted p value	Intersection size
GO:MF[1]	binding	4.90E-22	1772
GO:MF	protein binding	2.95E-28	1243
GO:MF	ion binding	1.67E-08	768
GO:BP[2]	cellular process	3.19E-07	2040
GO:BP	biological regulation	2.20E-07	1445
GO:BP	metabolic process	0.000179	28
GO:BP	regulation of gene expression	6.51E-05	1438
GO:BP	organic substance metabolic process	2.94E-07	1380
GO:BP	regulation of biological process	3.80E-07	1361
GO:BP	cellular metabolic process	8.63E-10	1353
GO:BP	primary metabolic process	7.21E-07	1315
GO:BP	regulation of cellular process	3.20E-06	1302
GO:CC[3]	intracellular	9.16E-55	1850
GO:CC	organelle	1.59E-41	1670
GO:CC	intracellular organelle	9.85E-47	1645
GO:CC	membrane-bounded organelle	9.28E-45	1509
KEGG	Cell cycle	5.18E-06	44
KEGG	RNA transport	0.00362	44
KEGG	Ubiquitin mediated proteolysis	0.039939	38
KEGG	ECM-receptor interaction	0.001189	28
KEGG	Progesterone-mediated oocyte maturation	0.013183	27
KEGG	Steroid biosynthesis	0.020207	10
REAC[4]	Cell Cycle	4.29E-20	130
REAC	Cell Cycle, Mitotic	4.52E-19	120
REAC	M Phase	1.31E-13	89
REAC	Signaling by Rho GTPases	0.000398	82
REAC	Mitotic Prometaphase	4.11E-10	61
WP[5]	Cholesterol Biosynthesis	0.004512	8
HP[6]	Abnormality of head or neck	0.024855	501
HP	Abnormality of the head	0.045422	496
HP	Abnormal nervous system morphology	0.003602	483
HP	Abnormality of the musculature	0.000272	466
HP	Abnormality of the face	0.009479	460
TF[7]	Factor: Cdx-1; motif: TTTATK	2.02E-08	1721
TF	Factor: Pax-5; motif: RRMSWGANWYCTNRAGCGKRACSRYNSM	0.000343	1662
TF	Factor: Churchill; motif: CGGGNN	0.00844	1631
TF	Factor: HOXA13; motif: ATAAMA	7.97E-06	1582
TF	Factor: E2F; motif: GGCGSC	8.13E-30	1541

1. Gene Ontology Molecular Function
2. Gene Ontology Biological Process
3. Gene Ontology Cellular Component
4. Reactome
5. Wiki Pathways
6. Human phenotype
7. Transcription factors

Supplementary Table S9: Upstream activators of DEGs identified after H5N1 HPAI challenge

Upstream Regulator	Molecule Type	Predicted Activation State	Activation z score	p-value of overlap	Target Molecules in Dataset	Mechanistic Network
IFNG	cytokine	Activated	3.332	0.000000122	ALKAL2,CALHM6,CCL19,CCL4,CCN2,CHAC1,C	94 (21)
IFNB1	cytokine	Activated	2.762	0.00000851	CCL4,CMPK2,CYP3A5,F2R,HLA-A,IFI6,IL6,IRF7,	94 (19)
MSC	transcription regulator	Activated	2.236	0.000487	CCL4,DDX60,EPSTI1,IRF7,SPP1	
SPI1	transcription regulator	Activated	2.529	0.000579	CCL4,CMPK2,CSF2RA,FOXO1,IFI6,IRF7,MX1,O	118 (21)
TGM2	enzyme	Activated	2.63	0.00127	ATP1B3,DDX60,IFI6,IFIT5,IL6,OASL,RNF213,SA	57 (7)
TMPRSS2-ERG	fusion gene/product	Activated	2.236	0.0165	ARHGAP28,CHAC1,CSTA,OASL,SAMD9L	
HDAC4	transcription regulator	Activated	2.195	0.0221	IL6,KLF2,MMP13,MYH10,RSAD2	
NR3C1	ligand-dependent nuclear receptor	Inhibited	-2.107	0.00000319	BMP2,CCL4,COQ8B,CXCR4,CYP3A5,DUSP1,DU	110 (17)
SIRT1	transcription regulator	Inhibited	-2.385	0.0000111	CMPK2,COL10A1,DDX60,FOXO1,HLA-A,IL6,IR	84 (19)
MAPK1	kinase	Inhibited	-2.05	0.0000114	CCL4,CCN2,CSTA,DUSP1,FOXO1,IFI6,IFIT5,IL6,	105 (20)
PTGER4	G-protein coupled receptor	Inhibited	-2.449	0.0000124	CCL4,CMPK2,CXCR4,IL6,IRF7,MMP13,RNF213,	101 (25)
MRTFB	transcription regulator	Inhibited	-2.183	0.000149	CCN2,CXCR4,F2R,FILIP1L,HLA-A,MYH10,PDG	
NKX2-3	transcription regulator	Inhibited	-2.53	0.000245	BMP2,CMPK2,DDX60,GCH1,KLF2,PMAIP1,RNF	
LMNA	other	Inhibited	-2	0.000898	ACKR4,BAMBI,CCN2,DUSP4,FLT1,KCNQ1,NES,	70 (12)
TET2	enzyme	Inhibited	-2.39	0.00127	CMPK2,IL6,KLF2,MMP13,RSAD2,SLC4A11,ZBT	
IL4	cytokine	Inhibited	-2.409	0.00143	ADRM1,BPGM,CCL4,CMPK2,CXCR4,F2R,F3,FL	81 (17)
RNF31	enzyme	Inhibited	-2	0.0016	CCL19,CCL4,CXCR4,IL6,KCNMA1	94 (13)
ESR1	ligand-dependent nuclear receptor	Inhibited	-2.16	0.0018	AP1S3,BMP2,CAV2,CCL4,CLDN1,COTL1,DOCK	105 (17)
Esrra	transcription regulator	Inhibited	-2.236	0.00221	ATP5MF,ATP5PO,BPGM,IL6,KCNQ1,SPP1	
WNT3A	cytokine	Inhibited	-2.027	0.00252	ANGPT1,BMP2,CCN2,IL6,LRRC32,MMP13,NDN	90 (16)
CLEC4G	other	Inhibited	-2	0.00412	BMP2,CCN2,IL6,WNT2B	
MYC	transcription regulator	Inhibited	-2.055	0.00416	ANGPT1,CHST15,DUSP1,DUSP4,EED,F2R,F3,HL	114 (23)
IL10	cytokine	Inhibited	-3.088	0.00636	BMP2,CCL19,CCL4,CXCR4,DUSP1,F3,IL6,LY96,	104 (21)
IL10RA	transmembrane receptor	Inhibited	-2.985	0.0101	BMP2,CALHM6,HLA-A,IL6,IRF7,PDGFRA,RNF2	
STAT6	transcription regulator	Inhibited	-2.203	0.0109	CMPK2,FNIP2,IL6,IRF7,MMP13,MPEG1,OASL,R	
BMP10	growth factor	Inhibited	-2	0.023	ANGPT1,CMPK2,HLA-A,RSAD2	
Tgf beta	group	Inhibited	-2.156	0.0231	BAMBI,CCL4,CCN2,COL10A1,CXCR4,IL6,MMP1	
SATB1	transcription regulator	Inhibited	-2.2	0.0434	DUSP4,EPSTI1,IRF7,KLF2,UHRF1	
RARB	ligand-dependent nuclear receptor	Inhibited	-2	0.14	IL6,REG1A,SPP1,USP18	
RARA	ligand-dependent nuclear receptor	Inhibited	-2	0.24	DUSP1,IL6,NR2C1,RSAD2,SPP1,TNFAIP8L1	
